# Galaxy CLIP-Explorer: a web server for CLIP-Seq data analysis

**DOI:** 10.1093/gigascience/giaa108

**Published:** 2020-11-11

**Authors:** Florian Heyl, Daniel Maticzka, Michael Uhl, Rolf Backofen

**Affiliations:** Bioinformatics Group, Department of Computer Science, University of Freiburg, Georges-Köhler-Allee 106, 79110 Freiburg, Germany; Bioinformatics Group, Department of Computer Science, University of Freiburg, Georges-Köhler-Allee 106, 79110 Freiburg, Germany; Bioinformatics Group, Department of Computer Science, University of Freiburg, Georges-Köhler-Allee 106, 79110 Freiburg, Germany; Bioinformatics Group, Department of Computer Science, University of Freiburg, Georges-Köhler-Allee 106, 79110 Freiburg, Germany; Signalling Research Centres BIOSS and CIBSS, University of Freiburg, Schaenzlestr. 18, 79104 Freiburg, Germany

**Keywords:** CLIP-Seq, data analysis, Galaxy, RNA, protein

## Abstract

**Background:**

Post-transcriptional regulation via RNA-binding proteins plays a fundamental role in every organism, but the regulatory mechanisms lack important understanding. Nevertheless, they can be elucidated by cross-linking immunoprecipitation in combination with high-throughput sequencing (CLIP-Seq). CLIP-Seq answers questions about the functional role of an RNA-binding protein and its targets by determining binding sites on a nucleotide level and associated sequence and structural binding patterns. In recent years the amount of CLIP-Seq data skyrocketed, urging the need for an automatic data analysis that can deal with different experimental set-ups. However, noncanonical data, new protocols, and a huge variety of tools, especially for peak calling, made it difficult to define a standard.

**Findings:**

CLIP-Explorer is a flexible and reproducible data analysis pipeline for iCLIP data that supports for the first time eCLIP, FLASH, and uvCLAP data. Individual steps like peak calling can be changed to adapt to different experimental settings. We validate CLIP-Explorer on eCLIP data, finding similar or nearly identical motifs for various proteins in comparison with other databases. In addition, we detect new sequence motifs for PTBP1 and U2AF2. Finally, we optimize the peak calling with 3 different peak callers on RBFOX2 data, discuss the difficulty of the peak-calling step, and give advice for different experimental set-ups.

**Conclusion:**

CLIP-Explorer finally fills the demand for a flexible CLIP-Seq data analysis pipeline that is applicable to the up-to-date CLIP protocols. The article further shows the limitations of current peak-calling algorithms and the importance of a robust peak detection.

## Findings

### Background

RNA plays a fundamental role in many regulatory processes like splicing and translation. Yet, processes like translation also undergo regulatory steps involving proteins such as elongation factors. These RNA-binding proteins (RBPs) interact with their target RNA and form ribonucleoprotein complexes [1]. Studies have revealed the involvement of RBPs in stages such as splicing, polyadenylation, localization, translation, stability, and degradation [2–5]. So far >1,000 RBPs have been identified in human cells [2,6, 7]. Various RBPs have been linked to neurodegenerative diseases and various types of cancer [2,4, 8, 9]. These observations emphasize the importance of exploring the mechanisms behind the regulatory processes mediated by RBPs.

Cross-linking and immunoprecipitation (CLIP) facilitates the analysis of the interdependence between the proteome and transcriptome *in vivo* [10] by detecting binding sites for RBPs on a genome-wide level. Many CLIP protocols such as PAR-CLIP [11], iCLIP [12], or eCLIP [13] emerged over a short period and new methods are still in development [14]. All methods consist of 3 fundamental steps: cross-linking the RBP of interest to its target RNAs, purification and immunoprecipitation of the resulting complexes, and high-throughput sequencing of the resulting RNAs. Despite these commonalities, protocols like iCLIP or eCLIP perform additional steps to increase the precision of the CLIP-Seq experiment [13, 15–17], which have to be covered by additional analysis tasks. For example, iCLIP introduced random barcodes (unique molecular identifiers [UMIs]) to reduce the number of duplicated reads [12]. Such protocols as eCLIP [13] and uvCLAP [18] adapted this procedure. A deduplication step is therefore imperative for iCLIP, eCLIP, FLASH, and uvCLAP [12,19].

Because of the complexity and variety of CLIP protocols, the computational analysis is still the critical bottleneck, in both time and reproducibility. Individual tools that perform quality control, mapping, peak calling, and motif detection for CLIP-Seq data exist. However, an automatic and complete data analysis pipeline has to deal with a big list of obstacles such as biases that are introduced by the CLIP-Seq protocol and experimental conditions. On top of this, additional problems arise from changing hardware and tool versions, practicality of the user interface, different library formats (e.g., biological replicates or multiplexed data), and different CLIP-Seq data formats for old, recent, or upcoming protocols. Furthermore, each tool for each subtask has different assumptions and parameters that need to be optimized for the underlying protocol [19]. The most challenging task is the binding site identification, where a couple of different peak callers, such as Piranha [1], PEAKachu [20], CLIPper [21], and PureCLIP [22], exist. For example, biological replicates are not supported by some peak callers including Piranha [1]. These obstacles lead to a lack of reproducibility for the CLIP-Seq data analysis.

One possible solution could be 1 big but fixed pipeline that can cope with every possible type of data. This solution has already been tried in the case of PIPE-CLIP [23] and CLIPSeqTools [24]. Nevertheless, it is intractable to cover all possible combinations of different experimental settings, such as the number of replicates, the existence of a control library, and others. Focussing instead on 1 specific type of data is easier to handle, such as analyzing only iCLIP data with iCount [25]. However, neither PIPE-CLIP nor CLIPSeqTools and iCount can be quickly and simply expanded or modified. They lack the option for an extension to cover noncanonical experimental data or new CLIP-Seq data types such as eCLIP, FLASH, or uvCLAP.

We hereby present CLIP-Explorer (https://clipseq.usegalaxy.eu/), a CLIP-Seq pipeline implemented in Galaxy [26]. CLIP-Explorer provides all necessary tools to analyze eCLIP, FLASH, uvCLAP, and iCLIP data. CLIP-Explorer is well documented through an online tutorial in the Galaxy training material [27] and the main domain. Both websites assist the user in understanding the main steps and parameters of the pipeline and the featured tools. The user can then, for example, replace the peak caller or read mapper. Detailed knowledge about the tools is not required. CLIP-Explorer works in a server environment; thus the user does not have to worry about varying hardware or tool versions. The constant maintenance of CLIP-Explorer makes the data analysis easy to reproduce.

We have validated CLIP-Explorer on eCLIP data of DROSHA, HNRNPK, IGF2BP1, KHDRBS1, LIN28B, PTBP1, QKI, SLBP, and U2AF2. We compared the results with a different analysis pipeline and databases, finding great diversity in the number of predicted peaks and motifs found. A more comprehensive analysis including the peak callers Piranha, PureCLIP, and PEAKachu was done for RBFOX2 [13] because it has well-documented targets and motifs. The protein RBFOX2 encoded by the gene *RBM9* is a tissue-specific splicing factor involved in developmental processes [21,28]. Studies have shown RBFOX2’s binding preference for introns close to differentially spliced exons [29, 30]. The conserved sequence motif TGCATG has been shown to be enriched in RBPFOX2’s binding sites [21,29,30]. Concerning the inconsistent results of the peak calling, we propose standard guidelines for the peak calling for different experimental set-ups. We confirm RBFOX2’s binding characteristics from the literature as another validation for CLIP-Explorer.

### CLIP-Explorer: A versatile pipeline for the analysis of CLIP-Seq data

**Figure 1:**
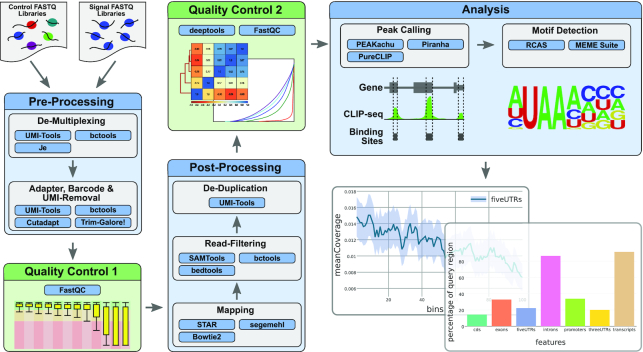
Flow chart of CLIP-Explorer; CLIP-Explorer has 3 major steps. In the pre-processing, the read library is demultiplexed, if necessary, and adapter sequences as well as in-line barcodes and UMIs are removed. CLIP-Explorer uses Je [31], UMI-Tools [32], bctools [33], and Cutadapt [34] for that purpose. A quality control step using FastQC [35] follows the pre-processing. In the post-processing, the reads are aligned with STAR [36], Bowtie [37], or segemehl [38], filtered using SAMtools [39], bedtools [40], and bctools, and deduplicated with UMI-tools. Another quality control, mainly with deeptools [41], checks the batch quality. Finally, CLIP-Explorer identifies differentially enriched regions using either PEAKachu [20], Piranha [1], or PureCLIP [22]. The binding regions are then analyzed with RCAS [42] and MEME-ChIP [43]. fiveUTRs, 5′ untranslated regions; threeUTRs, 3′ untranslated regions. mapping, quality control, peak calling, sequence motifs, structure motifs.

Different experimental settings require different analysis pipelines because pre-processing, mapping, peak calling, and motif detection have to be adapted. For that reason, CLIP-Explorer integrates several pipelines for analyzing different protocols, namely, eCLIP, iCLIP, FLASH, and uvCLAP. Common to all pipelines in CLIP-Explorer is the division into 4 major steps (Fig. 1). In the pre-processing, the read library is demultiplexed and, if necessary, adapter sequences as well as in-line barcodes and UMIs are removed. In the post-processing, the reads are aligned and deduplicated. CLIP-Explorer then identifies differentially enriched regions (peaks) that are further analyzed according to genomic localization and other criteria to investigate the precise function of the protein and properties of its targets. All subtasks are accompanied by quality control steps. The versatility of CLIP-Explorer allows the user to select 3 different peak-calling pipelines for 3 different data specifications. The Methods section covers CLIP-Explorer in more detail. Additional information can be found in the Galaxy training material.

### Recommendations for PEAKachu, Piranha, and PureCLIP

To this day, a benchmarking dataset for CLIP-Seq data analysis does not exist because of missing experimental methods to verify predicted binding sites. It is therefore recommended to test >1 peak caller and >1 parameter set, which is easily possible with CLIP-Explorer.

The newest version of PEAKachu [20] is well suited for an experimental set-up with ≥2 replicates for the CLIP experiment and ≥2 replicates for the control experiment because it uses DESeq2 [44]. It is therefore best to turn on the DESeq2 normalization. If the user has fewer replicates, PEAKachu can still be used, but DESeq2 requires ≥2 replicates for both experiment and control to calculate *P*-values. With <2 replicates, PEAKachu filters the peaks on the basis of the fold change and the median absolute deviation (MAD) multiplier. It is therefore wise to check the peaks with another peak caller or peak-calling pipeline. PEAKachu works best in the adaptive mode with a MAD multiplier of 0, a log_2_ fold change threshold of 2.0, and an adjusted *P*-value threshold of 0.05. PEAKachu needs the parameter of the maximum insert size, identified beforehand by Picard [45]. The estimation of the insert size is only necessary if the user provides paired-end read data. The window mode of PEAKachu is not recommended because it is rather unstable. The MAD multiplier might reduce the number of peaks because it works as a second cut-off. To get the full peak set, it is wise to leave it at zero and filter the peaks by the log_2_ fold change together with the adjusted *P*-value. One key parameter is the minimum block overlap, which has to be tested with the default of 0.5 in the beginning. The user has to increase this parameter if the results show a lot of peaks within a close vicinity. Another critical parameter is the minimum cluster expression fraction and the minimum block expression. These parameters can change the total number of predicted peaks. Leave them in default with 0.01 and 0.1 and adjust the parameters if some interesting binding regions are not covered by PEAKachu’s prediction. PEAKachu can be applied to iCLIP, eCLIP, FLASH, uvCLAP, and even older protocols such as PAR-CLIP.

Piranha [1] can be applied to an experimental set-up with or without control, but it does not support replicates for the CLIP experiment. Each replicate has to be treated separately or further validated with a robust peak detection. However, if the user has only 1 replicate and no control, we recommend using Piranha. If a control is provided, Piranha uses a zero-truncated negative binomial regression by default. Without a control it is wise to stick to a negative binomial. The distance to merge significant bins is one of the most crucial parameters of Piranha, similar to the minimum block overlap of PEAKachu. If the user observes a lot of peaks within a close vicinity, then this parameter has to be increased (e.g., to 10). The bin size of the signal and control is another crucial parameter of Piranha that needs to be optimized. If the bin size is quite big (e.g., 200), then Piranha might miss a few good candidates. If the bin size is very small (e.g., 5), then Piranha makes a lot of false-positive predictions. Piranha can also be applied to iCLIP, eCLIP, FLASH, uvCLAP, and even older protocols such as PAR-CLIP.

PureCLIP [22] can be applied to an experimental set-up with or without control, but it does not support replicates by the time of our analysis. It is therefore best to apply PureCLIP, as well as Piranha, to each replicate separately and find robust peaks by intersection, merging, or calculating an irreducible discovery rate (IDR). Therefore, we recommend using PureCLIP if the user has only 1 replicate for the CLIP and control experiment. PureCLIP already incorporates 2 default parameter sets. One set can be used if the protein is assumed to bind low-complexity motifs, which results in broader and less specific binding sites. PureCLIP predicts not only the binding region but also the cross-linking sites. In our tests, PureCLIP quite often reported very small binding regions, almost identical to the cross-linking sites. We therefore recommend slightly extending the predicted binding sites (e.g., 5–10 bases to the left and right) to cover the whole binding region. Furthermore, if the user provides paired-end reads, the mate containing the cross-linking event has to be provided explicitly. For iCLIP, FLASH, and uvCLAP this corresponds to the first mate, while for eCLIP it is the second mate. PureCLIP was specifically designed for eCLIP and iCLIP [22]. We recommend using the peak caller only for those protocols or other variants such as FLASH or uvCLAP.

It is not easy to find a standard peak-calling algorithm with a standard parameter set. We tried to cover possible cases and recommendations for Piranha, PEAKachu, and PureCLIP, but these tools can change over time, or a new peak caller might outrank them. The user can therefore find permanently updated recommendations and guidelines for CLIP-Seq data analysis on CLIP-Explorer’s main domain.

### Effects of using different peak callers for RBFOX2

**Figure 2:**
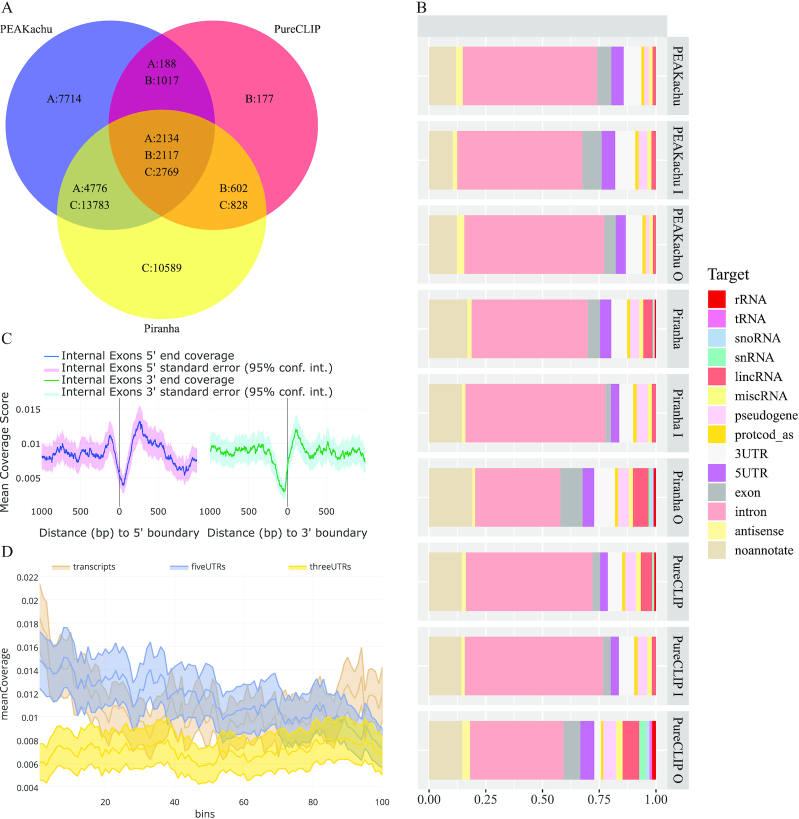
Comparison and analysis of the binding regions detected by PEAKachu (A), Piranha (B), and PureCLIP (C). The results are from the eCLIP data of RBFOX2 [13]. (A) We intersected the binding regions identified by the peak callers with bedtools [40], paying attention to the strand (intersect “-s"), to assess the robustness of each method. (B) We then annotated the binding regions of each peak caller and plotted the fraction of each target. We also analyzed separately the peak set of the individual peak callers (O = peak set 7,714/177/10,589) and the peak set of the intersection with all peak callers (I = peak set 2,134/2,117/2,769). RBFOX2 prevalently binds introns, but also 3′ and 5′ UTRs as well as lincRNAs. The plot was generated with the hg19 script of targetdist [46]. Investigating the mean coverage of these binding regions identified by (C) PEAKachu [20] reveals an occupancy drop around the 5′ and 3′ ends of the exons. (D) Looking further at the mean coverage of the binding regions identified by PEAKachu in the overall transcript as well as for the 5′ and 3′ UTRs. The thickness of the ribbon around the mean coverage indicates the $95\%$ confidence interval (mean ± standard error of the mean × 1.96). Each feature is divided into 100 bins of equal length, whereas features smaller than 100 bp are excluded [42]. lincRNA: long noncoding RNA; rRNA: ribosomal RNA; snRNA: small nuclear RNA; snoRNA: small nucleolar RNA; tRNA: transfer RNA.

We use eCLIP data from DROSHA, HNRNPK, IGF2BP1, KHDRBS1, LIN28B, PTBP1, QKI, RBFOX2, SLBP, and U2AF2 from the study by Van Nostrand et al. [13] to validate CLIP-Explorer (see Methods). We first use RBFOX2 data to check the robustness and quality of the predicted binding sites of CLIP-Explorer because the protein has a well-known binding motif. We intersect the peaks of Piranha, PEAKachu, and PureCLIP with bedtools [40] (see Methods). Piranha detects the highest number of potential binding regions for RBFOX2 (Fig. 2A). Yet, more than one-third of Piranha’s peaks are not included in PEAKachu’s and PureCLIP’s peak set. PureCLIP has the highest fraction of peaks shared with the other 2 peak callers, but it also has the lowest total number of peaks. PEAKachu on the other hand also has a high number of individual peaks, but substantially less than Piranha. To check for the origin of the difference in the number of peaks, we compare all 3 peak callers (PEAKachu, Piranha, PureCLIP), including the CLIPper peaks of the study by Van Nostrand et al. [13] for RBFOX2, regarding potential CLIP-Seq artifacts and biases. Table 1 shows that <$1\%$ of the peaks from all peak callers overlap with 3′ or 5′ untranslated regions (UTRs). PEAKachu has 362 peaks that overlap with repeats ($2.45\%$); however, all peak callers have a rate below $1\%$ of peaks that overlap with intron repeats or any RNA pseudogene region. We also check the peak length and the distance between the peaks of the different peak callers (Supplementary Table S1). PEAKachu has on average larger peaks, with a mean of 131 nucleotides. On the other hand, PureCLIP has the smallest peaks, with a mean of 36 nucleotides. The distribution of Piranha is a constant of 20 nucleotides because the peak length is a parameter the user defines for the tool. Furthermore, the distance between the peaks is almost identical between the peak callers PEAKachu, PureCLIP, and CLIPper (Supplementary Table S1). Only Piranha has slightly more peaks, which are close together.

**Table 1:** Percentage of peaks of the RBFOX2 peak set overlapping with the stated feature

Feature	PEAKachu	Piranha	PureCLIP	CLIPper
3′ UTR	0.42	0.02	0.03	0.09
5′ UTR	0.96	0.05	0.03	0.27
Repeats	2.45	0.01	0.15	0.87
Intron repeats	0.04	0	0	0
ncRNA pseudogenes	0.09	0.38	0.59	0.01
rRNA pseudogenes	0	0.07	0.05	0
tRNA pseudogenes	0	0	0	0

To identify the type of bound genomic regions, we annotate the peaks discovered by the 3 different peak callers. The distributions of binding sites generated by these 3 tools show a similar trend and prevalence for introns as the main target of RBFOX2 (Fig. 2B). Yet, introns are not the only target. Our findings suggest that some binding regions of RBFOX2 lie in 3′ and 5′ UTRs, but this fraction is not as big as for introns. PEAKachu, Piranha, and PureCLIP further detect another chunk of target sites in long noncoding RNAs (lincRNAs), but the portion detected by PEAKachu is smaller, probably because of a low coverage of lincRNAs in CLIP protocols. In addition, we annotated separately the peak set of the individual peak callers (7,714, 177, and 10,589, for PEAKachu, PureCLIP, and Piranha, respectively, in Fig. 2A) and the peak set of the intersection with all peak callers (2,134, 2,117, and 2,769, respectively). The target distribution of Piranha and PureCLIP changes for the individual peak set. The portion of intron coverage shrinks and the portion of exon targets increases, as well as lincRNA, ribosomal RNA (rRNA), small nucleolar RNA, and small nuclear RNA. In contrast, the target distributions of PEAKachu look similar. However, the target distribution of the intersection of all 3 peak callers looks different in comparison with the distribution of all peaks. PEAKachu’s distribution has a smaller intron and a bigger exon and pseudogene portion. On the other hand, the peaks from the intersection set of Piranha and PureCLIP overlap more with introns and less with exons or any small nuclear RNA, small nucleolar RNA, transfer RNA (tRNA), or rRNA.

To verify that RBFOX2 is a splicing factor, we investigate the peak profile of the binding sites by looking at the coverage plot of RBFOX2. PEAKachu depicts a decrease in the binding region coverage around the exon-intron boundaries at the 5′ and 3′ ends (Fig. 2C). The decrease is more intense for the sites found by PEAKachu. Checking the mean coverage of the binding regions (Fig. 2D), the results suggest a binding prevalence of RBFOX2 in the upstream region of the transcripts. Furthermore, RBFOX2 seems to target the beginning of the 5′ UTR. In contrast, the binding coverage is homogeneous for the 3′ UTR. The sequence UGCAUG seems to play an important part for the binding of RBFOX2 because it is among the top 5 motifs detected by the PEAKachu, Piranha, and PureCLIP pipeline (see Table 2). The second motif shows guanine richness, and the third motif, cytosine and uracil richness for all peak callers.

**Table 2:** Top 5 RBFOX2 sequence motifs for each peak caller identified by MEME-ChIP with E-value and the fraction of sequences with that specific motif, based on the RBFOX2 eCLIP data

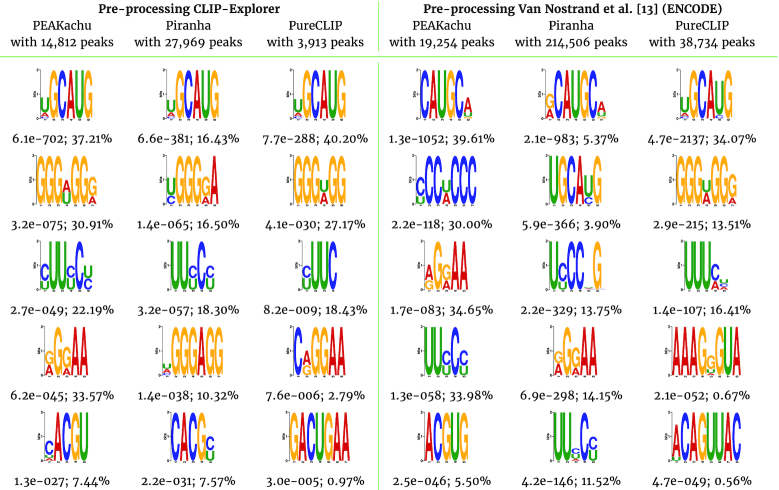

At the end, we check the function of RBFOX2 to clarify the role in human liver cancer cells (Hep G2). Looking at the top 100 genomic regions that have RBFOX2 binding sites, we find Shank2 and Shank3 among the top hits as potential targets. Furthermore, a gene ontology (GO) analysis with RCAS for the targets of RBFOX2 identifies the protein to be relevant for the regulation of RNA splicing (with a Benjamini-Hochberg [BH]-adjusted *P* < 10^−4^), as well as regulation of transcription by RNA polymerase I (BH-adjusted *P* < 10^−3^). In addition, the GO analysis identifies RBFOX2 as involved in the regulation of histone modifications (BH-adjusted *P* < 10^−4^), nucleosome and nucleosomal binding (BH-adjusted *P* < 10^−4^ and 0.04, respectively), and methyl-CpG binding (BH-adjusted *P* < 0.04).

Checking the results for RBFOX2, Piranha found the highest number of peaks. A lot of these peaks, however, might represent false-positive results because Piranha was executed without the information from the control experiments (see Methods). More than one-third of Piranha’s peaks were not included in PEAKachu’s and PureCLIP’s peak sets, which endorses the supposition. The observation is also substantiated by the change of the target distribution of the Piranha peaks that do not intersect with the peaks of the other 2 peak callers. These peaks might cover false-positive results that lie in unspecific regions such as rRNAs. Furthermore, the distance between the peaks is almost identical between the peak callers. Only Piranha has slightly more peaks, which are closer together, indicating a higher number of false-positive results because it calls many peaks (local maxima) in close proximity and does not combine them into a bigger peak (global maximum). In contrast, PureCLIP had the lowest number of predicted peaks and the highest fractions of peaks shared with the other peak callers. This indicates that PureCLIP selects the peaks on the basis of very stringent criteria. However, PureCLIP might therefore also have a high false-negative rate. PureCLIP calls peaks for each replicate separately, which is likely the reason that it misses some good candidates that have been jointly found by PEAKachu. Furthermore, the change of the target distribution for the PureCLIP peaks that do not intersect with the peaks of the other 2 peak callers might show possible false-positive results. As in Piranha’s distribution, peaks might overlap with unspecific regions such as rRNAs. PEAKachu, on the other hand, had the largest peaks. We cannot make a general judgement about a good value for the peak length because each RBP and each binding region can be specific and a benchmarking dataset is missing for CLIP-Seq data. PEAKachu’s target distribution for peaks that do not intersect with the peaks of the other 2 peak callers might suggest some false-positive results, because of the bigger portion of peaks in pseudogenes in that peak set. We also checked for potential CLIP-Seq biases (annotation from Ensembl) to answer the question of the different number of peaks, but we could not find any evidence regarding a significant UTR or repeat region bias for any of the implemented peak callers.

Despite the disparate numbers of peaks between PEAKachu, Piranha, and PureCLIP, the main motif of RBFOX2 with the sequence UGCAUG was identified with CLIP-Explorer for all peak callers used. The motif seems to be very robust to varying peak-calling conditions, which is endorsed by the fact that it can be found even with PureCLIP with a different pre-processing (see Table 2). We tested the peak callers PEAKachu, Piranha, and PureCLIP on the alignment files from the study by Van Nostrand et al. [13]. With CLIP-Explorer all 3 peak callers found the main motif with lower background noise in comparison to the already processed alignment files from ENCODE. Furthermore the motif set from CLIP-Explorer looks more similar between all 3 peak callers (see Table 2). It is therefore possible to find a better ground truth, that is to say, a benchmark set with CLIP-Explorer, because additionally >1 peak caller can be tested with just a few clicks.

We further tried to verify RBFOX2’s known role as splicing factor and that it preferably binds to introns [21, 28–30] to substantiate the credibility and versatility of CLIP-Explorer. The distributions of binding sites generated by PEAKachu, Piranha, and PureCLIP showed a prevalence for introns as the main target of RBFOX2 in accordance with the literature [29,30], which is further supported by the target distribution of the intersected peaks for all 3 peak callers. The decrease of the binding occupancy of RBFOX2 around the exon-intron boundaries can also be seen in other studies [29,30]. The binding coverage of RBFOX2 around the exon-intron boundaries suggests an involvement of RBFOX2 in the regulation of splicing. The GO term analysis corroborates the hypothesis linking RBFOX2 to various splicing and structure-related processes, such as histone modifications. Besides, the Shank gene family members, as a potential target of RBFOX2, play an important part in neuronal functions, where alterations in the encoded proteins may be connected to autism [21]. Shank2 and Shank3 might hereby be regulated by alternative splicing [47,48]. Another study has found the same interdependence of RBFOX2 and the Shank protein family [21].

### Comparison of CLIP-Explorer’s results

We compare the sequence motifs detected by CLIP-Explorer with 2 different databases [49,50], and then with the peaks identified in the study by Van Nostrand et al. [13] (Supplementary Table S2). Here, the CLIPper algorithm [21] was used to identify potential binding regions of the same proteins. For the CLIPper peaks, we predict the sequence motifs with MEME-ChIP in the same way as implemented in CLIP-Explorer.

As a first step, we focus on the sequence motifs that resulted from CLIP-Explorer using PEAKachu for peak calling. The motifs are similar and sometimes nearly identical to the motifs listed in the databases for HNRNPK, KHDRBS1, PTBP1, QKI, RBFOX2, and U2AF2 (Supplementary Table S2). For example, the QKI-motif ACUAA [51] or the known motif UGCAUG of RBFOX2 [21,29,30] can be found in the databases and is also detected by CLIP-Explorer and CLIPper. Some proteins such as DROSHA, LIN28B, and SLBP are not listed in the databases, and the proteins IGF2BP1 and RBFOX2 have only 1 or 2 motifs. CLIP-Explorer identifies new motifs for these proteins. Several of the new motifs detected by CLIP-Explorer are also detected by CLIPper but not covered by the databases [49,50] or any other literature to the best of our knowledge (Supplementary Table S2). For example, we find the new sequence motif CAGGCUGG for PTBP1 and the motif ACAG for U2AF2.

For some proteins, such as DROSHA and HNRNPK, the motifs detected by CLIP-Explorer deviate from the corresponding CLIPper motifs but still show similar sequence compositions (Supplementary Table S2). In addition, CLIPper slightly misses the known motif GGAGA of LIN28B [52].

For a more fine-grained comparison between the PEAKachu pipeline in CLIP-Explorer and the CLIPper pipeline in Van Nostrand et al. [13], we intersect the peaks for each protein with bedtools [40] to check for common binding sites. We require an overlap for bedtools of ≥1 base (see Methods). This comparison reveals a discrepancy between some proteins. Less than 10% of all PEAKachu peaks overlap with the CLIPper peaks of the protein KHDRBS1 (Supplementary Table S3). Quite often, PEAKachu finds more peaks than does CLIPper, except for QKI and U2AF2. For example, CLIPper finds 161 peaks and CLIP-Explorer (PEAKachu) 220 for the protein SLBP. Using PEAKachu, CLIP-Explorer predicts nearly 60 more peaks than the CLIPper pipeline. We suspect that this difference is due to the computational model of CLIPper, which calls peaks for every replicate separately. PEAKachu, on the other hand, calls with all replicates in mind. Therefore, we investigate the difference in the CLIPper peak sets for each replicate. The first replicate encompasses 7,942 CLIPper peaks and the second replicate 1,530 peaks, when we filter for entries with a *P*-value of 0.05 and a log_2_ fold change of 1, which makes a total difference of 6,412 peaks. Intersecting the peaks without an IDR results in 291 peaks, more than PEAKachu, and with an IDR CLIPper has the aforementioned 161 peaks, so less than PEAKachu. Thus, roughly $20\%$ of the CLIPper peaks in replicate 1 are contained in replicate 2. Consequently, the difference in the peak set of PEAKachu (CLIP-Explorer) might come from a different noise estimation between replicates, thus detecting other binding sites. Because of the different number of peaks, we check for potential CLIP-Seq biases (Supplementary Table S4). Both PEAKachu and CLIPper have no peaks in repeat or pseudogene regions, except for 1 PEAKachu peak in an ncRNA pseudogene. PEAKachu also finds more peaks that overlap with ncRNA genes (48 peaks) than CLIPper (17 peaks). We also check the number of peaks overlapping with histone genes because SLBP targets mainly histone RNAs. PEAKachu finds slightly more peaks in histone regions (163 peaks) than CLIPper (135 peaks). Consequently, PEAKachu covers slightly more peaks in histone regions, but also more peaks in ncRNAs genes, which might represent false-positive results because SLBP is known to target histone messenger RNA. The eCLIP pipeline from Van Nostrand et al. [13] already removed repeat regions in a double mapping approach.

**Figure 3:**
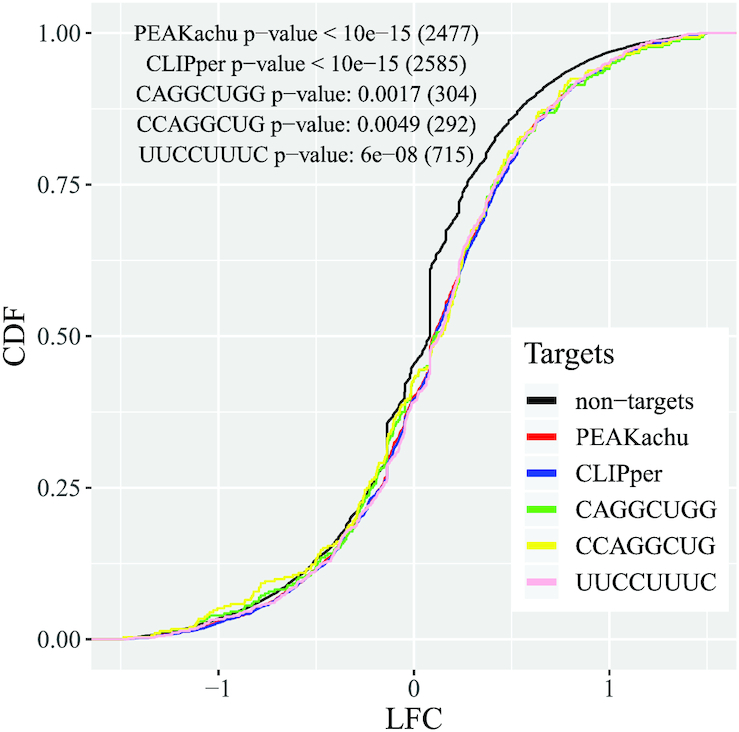
Cumulative density function (CDF) of the log_2_ fold change (LFC) of the genes of hg38 for a knockdown experiment of PTBP1. The identified targets of CLIPper and PEAKachu have nearly identical trends and show a significantly shifted CDF (*P*-values of 1-sided *t*-test in the plot), as well as the targets with the identified motifs CAGGCUGG (CLIP-Explorer), CCAGGCUG (CLIPper), and UUCCUUUC (CLIP-Explorer and CLIPper) with *P*-values of 1-sided Wilcoxon test, in comparison with the nontargets. The number of targets is listed after the *P*-value.

To check the correctness of the identified new motifs such as CAGGCUGG for PTBP1, we took independent RNA-Seq data from ENCODE from a knockdown and control experiment for PTBP1 (see Methods). We intersected the identified binding sites of PTBP1 from CLIP-Explorer (PEAKachu) and CLIPper with the genes of hg38 and calculated the log_2_ fold change of all genes. Fig. 3 clearly shows a significant shift in the cumulative density function (CDF) of the fold changes. This observation was expected because true binding sites should have different RNA rates in a knockdown experiment. Thus, the identified sites from PEAKachu and CLIPper might be real binding sites of PTBP1 because both peak callers show similar CDFs even though the processing of the data was different. Thus we checked the motif CAGGCUGG (from CLIP-Explorer), CCAGGCUG (from CLIPper), and another motif UUCCUUUC (from CLIP-Explorer and CLIPper). All 3 CDFs are significantly shifted. Consequently, CAGGCUGG might not be a false-positive result.

### Potential implications

CLIP-Explorer is a valuable tool for researchers working with CLIP-Seq data because it simplifies and integrates many processing steps in a well-tested and optimized pipeline. CLIP-Explorer provides the user with an extensive overview of the potential function of the RBP and its target RNAs. It can be easily extended or modified and has no installation overhead because it is integrated into Galaxy. CLIP-Explorer is thus the first general and fully automatic pipeline for eCLIP, FLASH, and uvCLAP data, which can also be applied to iCLIP and other types of CLIP-Seq protocols. Besides, it is permanently maintained, and new tools can be implemented or exchanged with existing ones to carry out a highly efficient data analysis.

We analyzed different eCLIP datasets and compared our findings with different databases [49,50] and the results from the study by Van Nostrand et al. [13]. The analysis of different proteins, such as DROSHA, HNRNPK, and in more detail RBFOX2, showed the strength of the flexibility provided by CLIP-Explorer. We could identify similar and even new sequence motifs for PTBP1 with the motif CAGGCUGG, and U2AF2 with ACAG. We also verified the sequence motif UGCAUG for RBFOX2 and found other guanine-, cytosine-, and uracil-rich binding regions. The most significant sequence motif UGCAUG of RBFOX2 was found by Piranha, PEAKachu, and PureCLIP, even though the 3 peak callers predicted 3 substantially different peak sets. On the basis of our results we recommend testing >1 peak-calling algorithm for other RBPs to assess the robustness of the motifs. We showed that different peak-calling tools might result in different peak sets and thus encompass different false-positive and false-negative results. However, CLIP-Explorer's user-friendly interface allows this very easily—the peak caller can be exchanged in an instant. CLIP-Explorer is also essential for a subsequent data investigation such as sequence motif detection or GO term analysis because it reduces noise in the data. CLIP-Explorer enabled us to identify the involvement of RBFOX2 in splicing because of the availability of specific annotation tools such as RCAS [42]. We were able to identify a decrease of RBFOX2 occupancy around the exon-intron boundaries, linked RBFOX2 to the regulation of splicing and DNA structural modifications, and verified other observations from previous studies.

We have recently integrated GraphProt [53], one of the popular tools for RBP binding profile predictions, into our pipeline. We will also include StoatyDive [54] in a later version to refine peak shape clustering in the hope of achieving better noise reduction because it showed promising results for CLIP-Seq data. All in all, CLIP-Explorer is a flexible and easily extendable pipeline, which greatly simplifies CLIP-Seq data analysis on a transcriptome- and genome-wide scale.

## Methods

CLIP-Explorer includes all major processes that are required to analyze CLIP-Seq data. All tools for each step were selected on the basis of review [17,19,55] or benchmark articles (stated below for each tool), or experiences. The analysis involves 3 major steps as shown in Fig. 1, each followed by a specific quality control. In the pre-processing step, the data are demultiplexed into the read libraries stemming from different experiments. If necessary, adapter sequences (using Cutadapt [34,56]) as well as in-line barcodes and UMIs are removed. During that step FastQC [35] performs a standard quality check for the read and library quality.

The post-processing for CLIP experiments is similar to RNA-Seq experiments; i.e., the reads are aligned and filtered. An additional deduplication step is required in the case of recent CLIP-Seq protocols such as iCLIP and eCLIP. The deduplication removes PCR duplication artifacts. CLIP-Explorer performs another quality control during the post-processing using mainly FastQC [35] and deeptools [41].

The final and major part of CLIP-Explorer is the analysis step. CLIP-Explorer searches in that step for sequence and coverage motifs, and potential targets of the investigated RBP. Peak calling and motif detection are the fundamental and most critical processes. The quality and amount of detected binding sites can vary significantly on the basis of the tools used, whereas the tools depend heavily on the experimental set-up. Different tools therefore lead in part to different results. For that reason, CLIP-Explorer provides several peak-calling and motif detection tools to determine the robustness of the results.

### Input and output of CLIP-Explorer

The user needs to provide their experimental data in FASTA or FASTQ format. Nonstandard adapter sequences can be provided by the user; otherwise, CLIP-Explorer automatically detects them. The pipeline is designed for multiplexed or demultiplexed paired-read data and supports replicates and control experiments. Barcode sequences are required in case of demultiplexing. Other additional files are provided by the Galaxy database. CLIP-Explorer can be easily changed, for example, to allow for single-end reads and different tool settings.

The user obtains a MultiQC [57] report for the raw reads, trimming, alignment, and deduplication to assess the quality of the raw data and important processing steps of CLIP-Explorer. MultiQC collects the FastQC reports made during the pre- and post-processing of CLIP-Explorer to inspect the mapping quality, elaborating on the amount of unmapped and multiply mapped reads, the length of mapped reads, and other important characteristics of the read library. Further quality control is provided by deeptools because it can elicit differences in signal and control experiments. A heat map and a fingerprint plot assess the correlation between the signal and control libraries, providing evidence for correct execution of the CLIP experiment. CLIP-Explorer also generates coverage files (bigWig and bedGraph) for the alignments and the cross-linking sites to inspect the peak-calling quality. Most importantly, CLIP-Explorer will produce a bed and gtf file of significantly enriched regions, representing the binding sites of the protein on the transcriptome or genome. A MEME-ChIP [43] report will further analyze the peaks, detecting potential sequence motifs of the protein. A FIMO [43] report then lists reference sequences, which were not covered by the peak calling, but contain the detected sequence motifs. Finally, an RCAS [42] report determines the target distribution of the protein over RNA classes and transcript regions. It also includes a GO term analysis and plots to reveal the coverage of the protein binding around splice junctions, along the transcripts and along various other regions. CLIP-Explorer can also generate a list of robust peaks (shared among all input files). This feature is useful for peak callers that do not support replicated data such as Piranha.

### Mapping and deduplication

We integrated STAR [36] into CLIP-Explorer to map reads against the genome based on the good performance and usability of STAR for RNA-Seq data [58–60]. STAR is an annotation- and splice-aware aligner, which is important for transcriptomic data. Thus, we used extra information about the transcriptome. The data of RBFOX2 were mapped against hg19 to better reproduce the literature results; all other proteins such as DROSHA and HNRNPK were mapped against hg38. STAR was executed with the “two pass mode" turned on and in the end-to-end alignment scheme. CLIP-Explorer further checks for incomplete pairs and ambiguously mapped and low-quality reads. CLIP-Explorer also includes a deduplication step to lower the false-positive rate for the identification of binding regions. PCR duplicates are often collapsed into 1 representative [2,19]. CLIP-Explorer identifies potential PCR duplicates with the help of UMI-tools [32]. Duplicated reads are identified after the alignment step, searching for reads with identical genomic positions (begin and end) and orientation. Yet, sequencing errors can also occur in the UMIs. UMI-tools clusters the reads on the basis of their UMI to handle these sequencing errors. Thus, we merged sequences with a high node count and a small Hamming distance between unique UMIs [32].

### Identification of enriched regions and sequence motifs

Searching for enriched regions and motifs is the most challenging task because of the differences in gene expression between CLIP experiments and background controls. Hence, high false-negative rates are a common result in the detection of differentially enriched regions (peaks) [19]. CLIP-Explorer allows the user to choose among 3 different peak callers, namely, PEAKachu, PureCLIP, and Piranha, which we picked on the basis of their performance, availability (i.e., open source and conda package), and support of different CLIP-Seq datasets with replicates or controls [1,20,22, 61–64].

PEAKachu was executed in adaptive mode with a MAD multiplier of 0, a log_2_ fold change threshold of 2.0, a BH-adjusted *P*-value threshold of 0.05, and a maximum insert size of 200, identified beforehand by Picard. All other parameters were set to their default values. For Piranha we used a negative binomial distribution with a bin size of 20 and a 0.05 *P*-value threshold. We omitted the control data for Piranha to test for possible experiments without control datasets. PureCLIP was trained on chromosome 1, 2, and 3 of hg38 (for RBFOX2 we used hg19, respectively) and executed with “-bc 0" as the default option. The resulting binding regions of PEAKachu, Piranha, and PureCLIP were then extended by 20 nucleotides because many peak callers often call peaks that stop before the motif itself. The resulting regions were analyzed with RCAS [42] to determine the target distribution over genomic regions and possible binding patterns of RBFOX2. The peaks were also analyzed with the MEME Suite [43] tool package (MEME-ChIP) to find sequence motifs in the peaks. We used MEME-ChIP because of its versatility and performance for known motifs [65,66]. Because of the missing ground truth for CLIP-Seq data, benchmarking for motif-finding tools remains to be carried out. MEME-ChIP was set to find 0 or 1 occurrence of the motif sites per sequence (ZOOPS model).

### Intersecting peaks

We used the intersect module of bedtools [40] to assess the occurrence of the predicted peaks between CLIPper and CLIP-Explorer with PEAKachu, and between the 3 different CLIP-Explorer pipelines with Piranha, PureCLIP, and PEAKachu. We set bedtools intersect with the option “-s" to search for intersections on the same strand and kept the default value for “-f," resulting in a minimum overlap of 1 base for overlapping regions to be reported. Further, we used the flag “-u" to consider only unique overlaps.

### Analyzed data

We used eCLIP data from DROSHA (ENCSR653HQC), HNRNPK (ENCSR828ZID), IGF2BP1 (ENCSR744GEU), KHDRBS1 (ENCSR628IDK), LIN28B (ENCSR861GYE), PTBP1 (ENCSR981WKN), QKI (ENCSR570WLM), RBFOX2 (ENCSR987FTF), SLBP (ENCSR483NOP), and U2AF2 (ENCSR202BFN) from the study by Van Nostrand et al. [13] to validate CLIP-Explorer. The data, which originated from human liver cancer cells (Hep G2) and immortalized myelogenous leukemia cells (K562), comprised 2 CLIP-Seq replicates and 1 control library for each RBP.

RBFOX2 was analyzed by CLIP-Explorer with PEAKachu, PureCLIP, and Piranha based on hg19. We took the peaks from the CLIPper pipeline (hg19 peaks ENCFF154DRN) and analyzed them with MEME-ChIP.

All other proteins were analyzed by CLIP-Explorer with PEAKachu on hg38. For the CLIPper pipeline we used the peaks from the IDR (signal normalization) from hg38 and analyzed them with MEME-ChIP. We investigated SLBP more thoroughly and used a more stringent parameter set for PEAKachu (i.e., no default) to find more robust peaks between replicates because CLIPper does the same with an IDR calculation. We set the fold change to 4, the MAD to 1.0, the minimum block overlap to 0.5, minimum cluster expression fraction to 0.001, and the minimum block expression to 0.6. This parameter set was optimal for the SLBP data, but it can be suboptimal for other proteins or CLIP-Seq data. To better compare the 2 tools, we also filtered for peaks that do not overlap with repeat regions and that have an annotated feature because CLIPper (i.e., the CLIPper pipeline) implements similar filter steps.

We also analyzed data from a short hairpin RNA knockdown experiment against PTBP1 in Hep G2 cells followed by RNA-Seq (ENCSR064DXG), including a control short hairpin RNA against no target (ENCSR603TCV). Both data samples are from ENCODE and encompass 2 replicates for each experiment. We took the alignments that were mapped with STAR against the genome (hg38). We calculated the coverage for each gene in hg38 with htseq-count [67]. We then obtained the log_2_ fold change for each gene from DESeq2 [44], taking both the knockdown and the control experiment into account. We then intersected the identified sites from PEAKachu and CLIPper for PTBP1 with the genes of hg38 and plotted the CDF of the log_2_ fold changes.

## Availability of Supporting Source Code and Requirements

Project name: CLIP-Explorer

Project home page: https://clipseq.usegalaxy.eu/

Operating system(s): Galaxy

Training material: https://galaxyproject.github.io/training-material/topics/transcriptomics/tutorials/clipseq/tutorial.html

biotools:CLIP-Explorer

RRID:SCR_018128

## Availability of Supporting Data and Materials

CLIP-Explorer provides a small dataset for a test run, which can be found in the training material and on the CLIP-Explorer website. The complete eCLIP data used in this article, such as RBFOX2 and PTBP1, are listed in the supplementary material of the study by Van Nostrand et al. [13].

## Additional Files

**Supplementary Table S1**.Comparison of the peak length and the distance between the peaks between PEAKachu, Piranha, PureCLIP, and CLIPper for the RBFOX2 data.

**Supplementary Table S2**. Top 5 DREME sequence motifs of MEME-ChIP [43] of different proteins. CLIP-Explorer’s sequence logos of different proteins from the binding regions that were identified by PEAKachu. Furthermore, sequence motifs of MEME-ChIP from the binding regions that were identified by the CLIPper algorithm [21]. The sequence motifs of CLIP-Explorer and CLIPper originated from eCLIP data [13]. To compare the sequence logos other motifs were collected from different databases [49, 50].

**Supplementary Table S3**. Peak intersections between PEAKachu [20] of CLIP-Explorer and CLIPper from the study by Van Nostrand et al. [13]. A list of Venn diagrams showing the overlap between PEAKachu [20] (CLIP-Explorer) and CLIPper peaks [13].

**Supplementary Table S4**. Number of peaks of the SLBP peak set of PEAKachu and CLIPper overlapping with the stated features. P-genes: pseudogenes.

## Abbreviations

BH: Benjamini-Hochberg; bp: base pairs; CLIP-Seq: cross-linking immunoprecipitation in combination with high-throughput sequencing; CDF: cumulative density function; GO: gene ontology; IDR: irreducible discovery rate; lncRNA: long noncoding RNA; MAD: median absolute deviation; RBP: RNA-binding protein; RCAS: RNA-centric annotation system; RNA-Seq: RNA sequencing; rRNA: ribosomal RNA; tRNA: transfer RNA; UMI: unique molecular identifier; UTR: untranslated region.

## Competing Interests

The authors declare that they have no competing interests.

## Funding

This study was funded by the Deutsche Forschungsgemeinschaft (DFG, German Research Foundation) grant 322977937/GRK2344 2017 MeInBio – BioInMe Research Training Group, Germany’s Excellence Strategy (CIBSS – EXC-2189 – Project ID 390939984), DFG grant BA2168/11-2 SPP 1738, DFG grant TRR 167/1 2027 NeuroMac, and by the Collaborative Research Centre 992 Medical Epigenetics (DFG grant SFB 992/2 2016).

## Authors' Contributions

F.H. and D.M. performed the computational analysis. R.B. and D.M. initiated the project and supervised the research. F.H. and R.B. wrote the manuscript with input from other authors. M.U. integrated GraphProt, PureCLIP, and RCAS into CLIP-Explorer. All authors read and approved the final manuscript.

## Supplementary Material

giaa108_GIGA-D-19-00287_Original_Submission

giaa108_GIGA-D-19-00287_Revision_1

giaa108_GIGA-D-19-00287_Revision_2

giaa108_GIGA-D-19-00287_Revision_3

giaa108_Response_to_Reviewer_Comments_Original_Submission

giaa108_Response_to_Reviewer_Comments_Revision_1

giaa108_Response_to_Reviewer_Comments_Revision_2

giaa108_Reviewer_1_Report_Original_Submission Silvia Bottini -- 11/19/2019 Reviewed

giaa108_Reviewer_1_Report_Revision_1 Silvia Bottini -- 3/18/2020 Reviewed

giaa108_Reviewer_1_Report_Revision_2 Silvia Bottini -- 7/30/2020 Reviewed

giaa108_Reviewer_2_Report_Original_Submission Eric Van Nostrand -- 11/22/2019 Reviewed

giaa108_Reviewer_2_Report_Revision_1 Eric Van Nostrand -- 3/19/2020 Reviewed

giaa108_Reviewer_2_Report_Revision_2 Eric Van Nostrand -- 7/13/2020 Reviewed

giaa108_Supplemental_Files
