# Supplementary material for: Galaxy CLIP-Explorer: a web server for CLIP-Seq data analysis
Source: Gigascience. 2020 Nov 11;9(11):giaa108. doi: 10.1093/gigascience/giaa108 (PMC7657819; doi:10.1093/gigascience/giaa108)
Supplement: giaa108_GIGA-D-19-00287_Original_Submission [file giaa108_giga-d-19-00287_original_submission.pdf]

GigaScience

Galaxy CLIP-Explorer: a web server for CLIP-Seq data analysis

--Manuscript Draft--

**Manuscript Number:**	GIGA-D-19-00287
**Full Title:**	Galaxy CLIP-Explorer: a web server for CLIP-Seq data analysis
**Article Type:**	Technical Note
**Funding Information:**	Deutsche Forschungsgemeinschaft (GRK 2344/1 2017 MeInBio) — Mr. Florian Heyl<br>Deutsche Forschungsgemeinschaft (BA2168/11-2 SPP 1738) — Mr. Michael Uhl<br>Deutsche Forschungsgemeinschaft (TRR 167/1 2027 NeuroMac) — Dr. Rolf Backofen<br>Deutsche Forschungsgemeinschaft (SFB 992/2 2016) — Dr. Rolf Backofen

Abstract:

Background

Post-transcriptional regulation via RNA-binding proteins (RBP) plays a fundamental role in every organism, but the regulatory mechanisms lack important understanding. Nevertheless, they can be fathomed by crosslinking immunoprecipitation in combination with high-throughput sequencing (CLIP-Seq). CLIP-Seq answers questions about the functional role of an RBP and its targets by determining binding sites on a nucleotide level and associated sequence and structural binding patterns. In recent years the amount of CLIP-seq data skyrocketed, urging the need for an automatic data analysis that can deal with different experimental setups. However, noncanonical data, new protocols, and a huge variety of tools, especially for peak calling, made it difficult to define a standard.

Findings

CLIP-Explorer is a flexible, and reproducible data analysis pipeline for iCLIP data that supports for the first time eCLIP, FLASH, and uvCLAP data. Individual steps like peak calling can be changed to adapt to different experimental settings. We validate CLIP-Explorer on eCLIP data, finding similar or nearly identical motifs for various proteins in comparison with other databases. In addition, we detect new sequence motifs for PTBP1, LIN28B, U2AF2, DROSHA, QKI, SLBP, and KHDRBS1. Finally, we optimized the peak calling with three different peakcallers on RBFOX2 data, discuss the difficulty of the peak calling step and give advice for different experimental setups.

Conclusion

CLIP-Explorer finally fills the demand for a flexible CLIP-Seq data analysis pipeline that is applicable to the up-to-date CLIP protocols. The paper further shows the limitations of current peak calling algorithms and the importance of a robust peak detection.

**Corresponding Author:**	Florian Heyl<br><br>GERMANY
**Corresponding Author Secondary Information:**	
**Corresponding Author's Institution:**	
**Corresponding Author's Secondary Institution:**	
**First Author:**	Florian Heyl
**First Author Secondary Information:**	
**Order of Authors:**	Florian Heyl<br>Daniel Maticzka<br>Michael Uhl<br>Rolf Backofen

Powered by Editorial Manager® and ProduXion Manager® from Aries Systems Corporation

Order of Authors Secondary Information:	
Additional Information:	
Question	Response
Are you submitting this manuscript to a special series or article collection?	No
**Experimental design and statistics**<br><br>Full details of the experimental design and statistical methods used should be given in the Methods section, as detailed in our Minimum Standards Reporting Checklist. Information essential to interpreting the data presented should be made available in the figure legends.<br><br>Have you included all the information requested in your manuscript?	Yes
**Resources**<br><br>A description of all resources used, including antibodies, cell lines, animals and software tools, with enough information to allow them to be uniquely identified, should be included in the Methods section. Authors are strongly encouraged to cite Research Resource Identifiers (RRIDs) for antibodies, model organisms and tools, where possible.<br><br>Have you included the information requested as detailed in our Minimum Standards Reporting Checklist?	Yes
**Availability of data and materials**<br><br>All datasets and code on which the conclusions of the paper rely must be either included in your submission or deposited in publicly available repositories (where available and ethically appropriate), referencing such data using a unique identifier in the references and in the "Availability of Data and Materials" section of your manuscript.	Yes

Have you have met the above requirement as detailed in our Minimum Standards Reporting Checklist?	

Manuscript
Click here to download Manuscript main.pdf

OXFORD (GIGA)^n SCIENCE

GigaScience, 2017, 1–10

doi: xx.xxxx/xxxx
Manuscript in Preparation
Technical Note

TECHNICAL NOTE

Galaxy CLIP-Explorer: a web server for CLIP-Seq data analysis

Florian Heyl[1,*], Daniel Maticzka[1], Michael Uhl[1] and Rolf Backofen[1,2,*]

[1]Bioinformatics Group, Department of Computer Science, University of Freiburg, Georges-Köhler-Allee 106, 79110 Freiburg, Germany and [2]Signalling Research Centres BIOSS and CIBSS, University of Freiburg, Schaenzlestr. 18, 79104 Freiburg, Germany

[*]heylf@informatik.uni-freiburg.de, backofen@informatik.uni-freiburg.de

Abstract

Background Post-transcriptional regulation via RNA-binding proteins (RBP) plays a fundamental role in every organism, but the regulatory mechanisms lack important understanding. Nevertheless, they can be fathomed by crosslinking immunoprecipitation in combination with high-throughput sequencing (CLIP-Seq). CLIP-Seq answers questions about the functional role of an RBP and its targets by determining binding sites on a nucleotide level and associated sequence and structural binding patterns. In recent years the amount of CLIP-seq data skyrocketed, urging the need for an automatic data analysis that can deal with different experimental setups. However, noncanonical data, new protocols, and a huge variety of tools, especially for peak calling, made it difficult to define a standard.
Findings CLIP-Explorer is a flexible, and reproducible data analysis pipeline for iCLIP data that supports for the first time eCLIP, FLASH, and uvCLAP data. Individual steps like peak calling can be changed to adapt to different experimental settings. We validate CLIP-Explorer on eCLIP data, finding similar or nearly identical motifs for various proteins in comparison with other databases. In addition, we detect new sequence motifs for PTBP1, LIN28B, U2AF2, DROSHA, QKI, SLBP, and KHDRBS1. Finally, we optimized the peak calling with three different peakcallers on RBFOX2 data, discuss the difficulty of the peak calling step and give advice for different experimental setups.
Conclusion CLIP-Explorer finally fills the demand for a flexible CLIP-Seq data analysis pipeline that is applicable to the up-to-date CLIP protocols. The paper further shows the limitations of current peak calling algorithms and the importance of a robust peak detection.

Key words: CLIP-Seq; Data Analysis; Galaxy; RNA; Protein

Findings

Background

RNA plays a fundamental role in many regulatory processes like splicing or translation. Yet, processes like translation also undergo regulatory steps involving proteins such as elongation factors. These RBPs (RNA-binding proteins) interact with their target RNA and form ribonucleoprotein complexes [1]. Studies have revealed the involvement of RBPs in stages like splicing, polyadenylation, localization, translation, stability, and degradation [2, 3, 4, 5]. So far more than a thousand RBPs have been identified in human cells [2, 6, 7]. Various RBPs have been linked to neurodegenerative diseases and various types of cancer [2, 4, 8, 9]. These observations emphasize the importance to explore the mechanisms behind the regulatory processes mediated by RBPs.

Crosslinking and immunoprecipitation (CLIP) facilitates the analysis of the interdependence between the proteome and transcriptome *in vivo* [10] by detecting binding sites for RBPs on a genome-wide level. Many CLIP protocols such as PAR-CLIP [11], iCLIP [12], or eCLIP [13] emerged over a short period of time and new methods are still in development [14]. All methods consist of three fundamental steps: crosslinking the

Compiled on: August 7, 2019.
Draft manuscript prepared by the author.

RBP of interest to its target RNAs, purification and immunoprecipitation of the resulting complexes, and high-throughput sequencing of the resulting RNAs. Despite these commonalities, protocols such as iCLIP or eCLIP perform additional steps to increase the precision of the CLIP-Seq experiment [13, 15, 16, 17], which have to be covered by additional analysis tasks. For example, iCLIP introduced random barcodes (unique molecular identifiers, short UMIs) to reduce the number of duplicated reads [12]. Protocols like eCLIP [13] and uvCLAP [18] adapted this procedure. A deduplication step is therefore imperative for iCLIP, eCLIP, FLASH, and uvCLAP [12, 19].

Because of the complexity and variety of CLIP protocols, the computational analysis is still the critical bottleneck, both in time and reproducibility. Individual tools that perform quality control, mapping, peak calling, and motif detection for CLIP-Seq data exist. However, an automatic and complete data analysis pipeline has to deal with a big list of obstacles such as biases that are introduced by the CLIP-Seq protocol and experimental conditions. On top of this, additional problems arise from changing hardware and tool versions, practicality of the user interface, different library formats (e.g., biological replicates or multiplexed data), and different CLIP-Seq data formats for old, recent, or upcoming protocols. Furthermore, each tool for each subtask has different assumptions and parameters that need to be optimized for the underlying protocol [19]. The most challenging task is the binding site identification, where a couple of different peakcallers, such as Piranha [1], PEAKachu [20], CLIPper [21], and PureCLIP [22], exist. For example, biological replicates are not supported by some peakcallers like Piranha [1]. These obstacles lead to a lack of reproducibility for the CLIP-Seq data analysis.

One possible solution could be one big, but fixed pipeline that can cope with every possible type of data. This solution was already tried in the case of PIPE-CLIP [23] or CLIPSeqTools [24]. Nevertheless, it is intractable to cover all possible combinations of different experimental settings, such as the number of replicates, the existence of a control library and others. Focussing instead on one specific type of data is easier to handle, like analyzing only iCLIP data with iCount [25]. However, neither PIPE-CLIP, nor CLIPSeqTools and iCount can be quickly and simply expanded or modified. They lack the option for an extension to cover noncanonical experimental data or new CLIP-Seq data types such as eCLIP, FLASH, or uvCLAP.

We hereby present CLIP-Explorer (https://clipseq.usegalaxy.eu/), a CLIP-Seq pipeline implemented in Galaxy [26]. CLIP-Explorer provides all necessary tools to analyze eCLIP, FLASH, uvCLAP and iCLIP data. CLIP-Explorer is well documented through an online tutorial in the Galaxy training material (https://galaxyproject.github.io/training-material/topics/transcriptomics/tutorials/clipseq/tutorial.html) and the main domain. Both websites assist the user to understand the main steps and parameters of the pipeline and the featured tools. The user can then, for example, replace the peakcaller or read mapper. It is not required to have detailed knowledge about the tools. CLIP-Explorer works in a server environment, thus the user does not have to worry about varying hardware or tool versions. A constant maintenance of CLIP-Explorer makes the data analysis easy to reproduce.

We have validated CLIP-Explorer on eCLIP data of DROSHA, HNRNPK, IGF2BP1, KHDRBS1, LIN28B, PTBP1, QKI, SLBP and U2AF2. We compared the results with a different analysis pipeline and databases, finding great diversity in the number of predicted peaks and found motifs. A more comprehensive analysis including the peakcallers Piranha, PureCLIP, and PEAKachu was done for RBFOX2 [13] as it has well documented targets and motifs. The protein RBFOX2 encoded by the gene RBM9 is a tissue-specific splicing factor involved in developmental processes [21, 27]. Studies have shown RBFOX2's binding preference for introns close to differentially spliced exons [28, 29]. The conserved sequence motif *TGCATG* has been shown to be enriched in RBPFOX2's binding sites [21, 28, 29]. Concerning the inconsistent results of the peak calling, we propose standard guidelines for the peak calling for different experimental setups. We confirm RBFOX2's binding characteristics from the literature as another validation for CLIP-Explorer.

CLIP-Explorer: A Versatile Pipeline for the Analysis of CLIP-seq Data

Different experimental settings require different analysis pipelines, since preprocessing, mapping, peak calling and motif detection have to be adapted. For that reason, CLIP-Explorer integrates several pipelines for analyzing different protocols, namely eCLIP, iCLIP, FLASH and uvCLAP. Common to all pipelines in CLIP-Explorer is the division into four major steps (Figure 1). In the preprocessing, the read library is demultiplexed and, if necessary, adapter sequences as well as in-line barcodes and UMIs are removed. In the postprocessing, the reads are aligned and deduplicated. CLIP-Explorer then identifies differentially enriched regions (peaks) that are further analyzed according to genomic localization and other criteria to investigate the precise function of the protein and properties of its targets. All subtasks are accompanied by quality control steps. The versatility of CLIP-Explorer allows the user to select three different peak calling pipelines for three different data specifications. The methods section covers CLIP-Explorer in more detail. Additional information can be found in the Galaxy training material.

The eCLIP data that we use to validate CLIP-Explorer comprises two CLIP-seq replicates and one control library for each RBP [13], which is supported by the PEAKachu peakcaller model. For that reason, we use the PEAKachu pipeline implemented in CLIP-Explorer to analyze the data. We use eCLIP data from DROSHA (ENCSR653HQC), HNRNPK (ENCSR828ZID), IGF2BP1 (ENCSR744GEU), KHDRBS1 (ENCSR628IDK), LIN28B (ENCSR861GYE), PTBP1 (ENCSR981WKN), QKI (ENCSR570WLM), RBFOX2 (ENCSR987FTF), SLBP (ENCSR483NOP), and U2AF2 (ENCSR202BFN) from the study by Nostrand et al. [13]. The data originating from human liver cancer cells (Hep G2) and immortalized myelogenous leukemia cells (K562). We first compare the sequence motifs detected by CLIP-Explorer with two different databases [43, 44], and then with the peaks identified in the study by Nostrand et al. [13] (Supplements: Table 1). Here, the CLIPper algorithm [21] was used to identify potential binding regions of the same proteins. For the CLIPper peaks, we predict the sequence motifs with MEME-ChIP in the same way as implemented in CLIP-Explorer. However, CLIP-Explorer maps the reads to hg38, except for RBFOX2 (hg19), whereas the pipeline of the study by Nostrand et al. [13] used hg19. We therefore convert the peak coordinates of the CLIPper algorithm from hg19 to hg38 with CrossMap [45].

Comparison of CLIP-Explorer's Results

As a first step, we scrutinize the quality of the sequence motifs that resulted from CLIP-Explorer using PEAKachu for peak calling. The motifs are similar and sometimes nearly identical to the motifs listed in the databases for HNRNPK, KHDRBS1, PTBP1, QKI, RBFOX2, and U2AF2 (Supplements: Table 1). For example, the QKI-motif *ACUAA* can be found in the databases and is also detected by CLIP-Explorer. Some proteins such as DROSHA, LIN28B, and SLBP are not listed in the databases, and the proteins IGF2BP1 and RBFOX2 have only one or two mo-

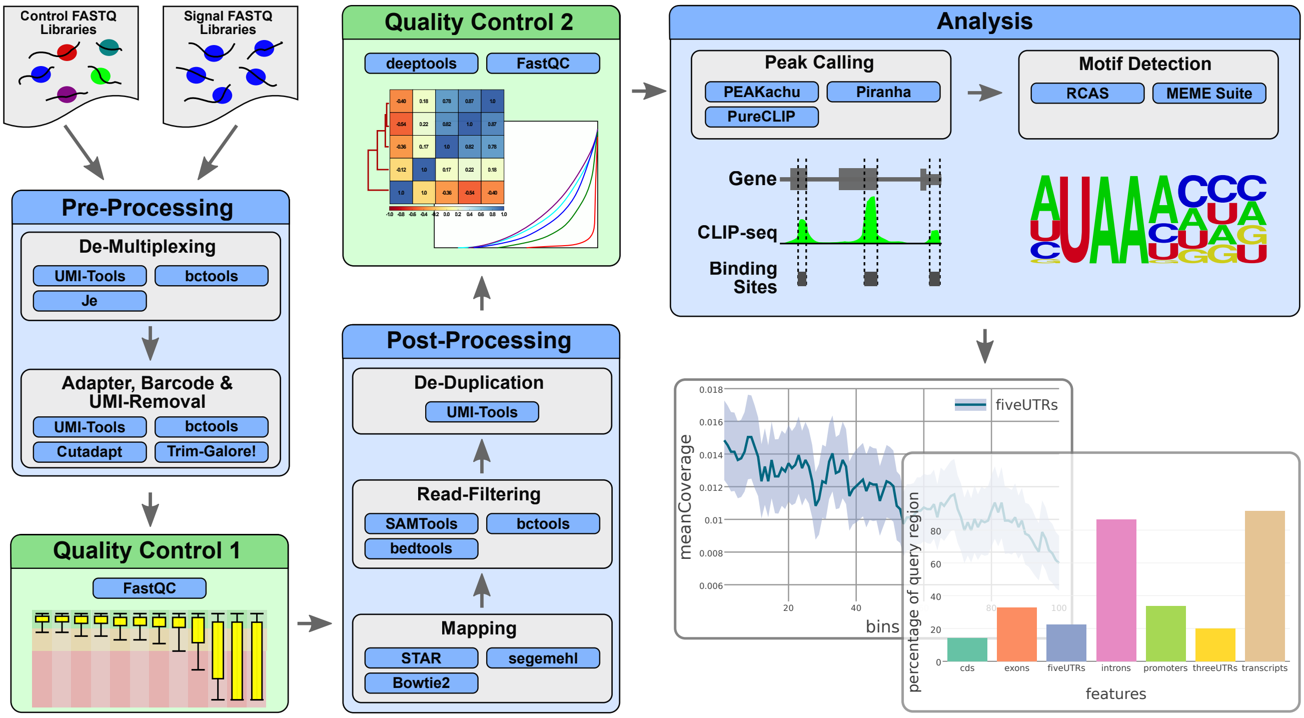

Figure 1. Flowchart of CLIP-Explorer; CLIP-Explorer has three major steps. In the preprocessing, the read library is demultiplexed, if necessary, and adapter sequences as well as in-line barcodes and UMIs are removed. CLIP-Explorer uses Je [30], UMI-Tools [31], bctools [32], and Cutadapt [33] for that purpose. A quality control step using FastQC [34] follows the preprocessing. In the postprocessing, the reads are aligned with STAR [35], Bowtie [36] or segemehl [37], filtered using SAMtools [38], bedtools [39], and bctools, and deduplicated with UMI-tools. Another quality control, mainly with deeptools [40], checks the batch quality. Finally, CLIP-Explorer identifies differentially enriched regions using either PEAKachu [20], Piranha [1], or PureCLIP [22]. The binding regions are then analyzed with RCAS [41] and MEME-ChIP [42].

tifs. CLIP-Explorer identifies new motifs for these proteins. Several of the new motifs detected by CLIP-Explorer are also detected by CLIPper but not covered by the databases [43, 44] or any other literature to the best of our knowledge (Supplements: Table 1). In more detail, we find the new sequence motif *CAGGCUGG* for PTBP1, whereas LIN28B has the additional sequence motif *CAGCCUG* or the short motif *CUCA*. Finally, we find additional binding motifs for U2AF2 (*ACAG*) and QKI (*AGGCU*).

For some proteins, the motifs detected by CLIP-Explorer had a larger deviation from the corresponding CLIPper motifs, but still show similar sequence compositions (Supplements: Table 1). DROSHA, for example, shows a prevalence for cytosine and uracil-rich regions. HNRNPK, on the other hand, has uracil and adenosine-rich binding sites. In addition, CLIPper sometimes misses important motifs in comparison to CLIP-Explorer. CLIPper does not find the known motif *UGCAUG* of RBFOX2 [21, 28, 29] and *GGAGA* of LIN28B [47] (Supplements: Table 1). CLIPper also slightly misses the uracil and adenosine richness of KHDRBS1's and QKI's binding regions, and the uracil richness of U2AF2. Furthermore, the CLIPper peaks for SLBP are not enough to predict sequence motifs with MEME-ChIP. Finding motifs for SLBP is only possible with CLIP-Explorer. Besides, the CLIPper peaks are too scant to find five significant motifs for KHDRBS1, thus only three motifs of MEME-ChIP (DREME [42]) are described (Supplements: Table 1).

For a more fine-grained comparison between the PEAKachu pipeline in CLIP-Explorer and the CLIPper pipeline in Nostrand et al. [13], we intersect the peaks for each protein with bedtools [39] to check for common binding sites. We use a strict overlap for bedtools of at least one base (see Methods). This comparison reveals a huge discrepancy between all proteins. Just one to three percent of all PEAKachu peaks overlap with CLIPper's set of peaks (Supplements: Table 2). Furthermore, PEAKachu finds more peaks in comparison to CLIPper. When we combine the peaks of CLIPper and CLIP-Explorer (PEAKachu) for each protein, the portion of peaks from CLIP-Explorer is bigger than the portion from CLIPper, except for QKI and U2AF2: DROSHA 57%, HNRNPK 65%, IGF2BP1 74%, KHDRBS1 95%, LIN28B 56%, PTBP1 51%, QKI 43%, RBFOX2 65%, SLBP 85%, and U2AF2 46%. For example, CLIPper finds 158 peaks and CLIP-Explorer (PEAKachu) 1052 for the protein SLBP. CLIP-Explorer predicts almost seven times more peaks than the CLIPper pipeline. We suspect that this difference is due to the computational model of CLIPper, which calls peaks for every replicate separately. Thus, we check the number of predicted peaks of the CLIPper pipeline for each replicate. The first replicate encompasses 9194 peaks, whereas the second replicate has 11686 peaks, which makes a total difference of 2492 peaks. Intersecting the peaks, without an irreducible discovery rate (IDR), of the two replicates with bedtools (see Methods) results in 1136 peaks.

Effect of Using Different Peakcallers for RBFOX2

To investigate the effect of using different peakcallers, we apply PEAKachu [20], PureCLIP [22], and Piranha [1] to optimize the prediction of the binding regions of RBFOX2. The eCLIP data of RBFOX2 [13] comprises two biological replicates and one size-matched input control [13] obtained from human liver cancer cells (Hep G2).

To check the robustness and quality of the predicted binding sites of CLIP-Explorer, we intersect the peaks of Piranha, PEAKachu, and PureCLIP with bedtools [39] (see Methods). Piranha detects the highest number of potential binding regions for RBFOX2 (Figure 2a). Yet, less more than one third of Piranha's peaks are not included in PEAKachu's and PureCLIP's peak pool. PureCLIP has the highest fraction of peaks shared with the other two peakcallers, but it also has the lowest to-

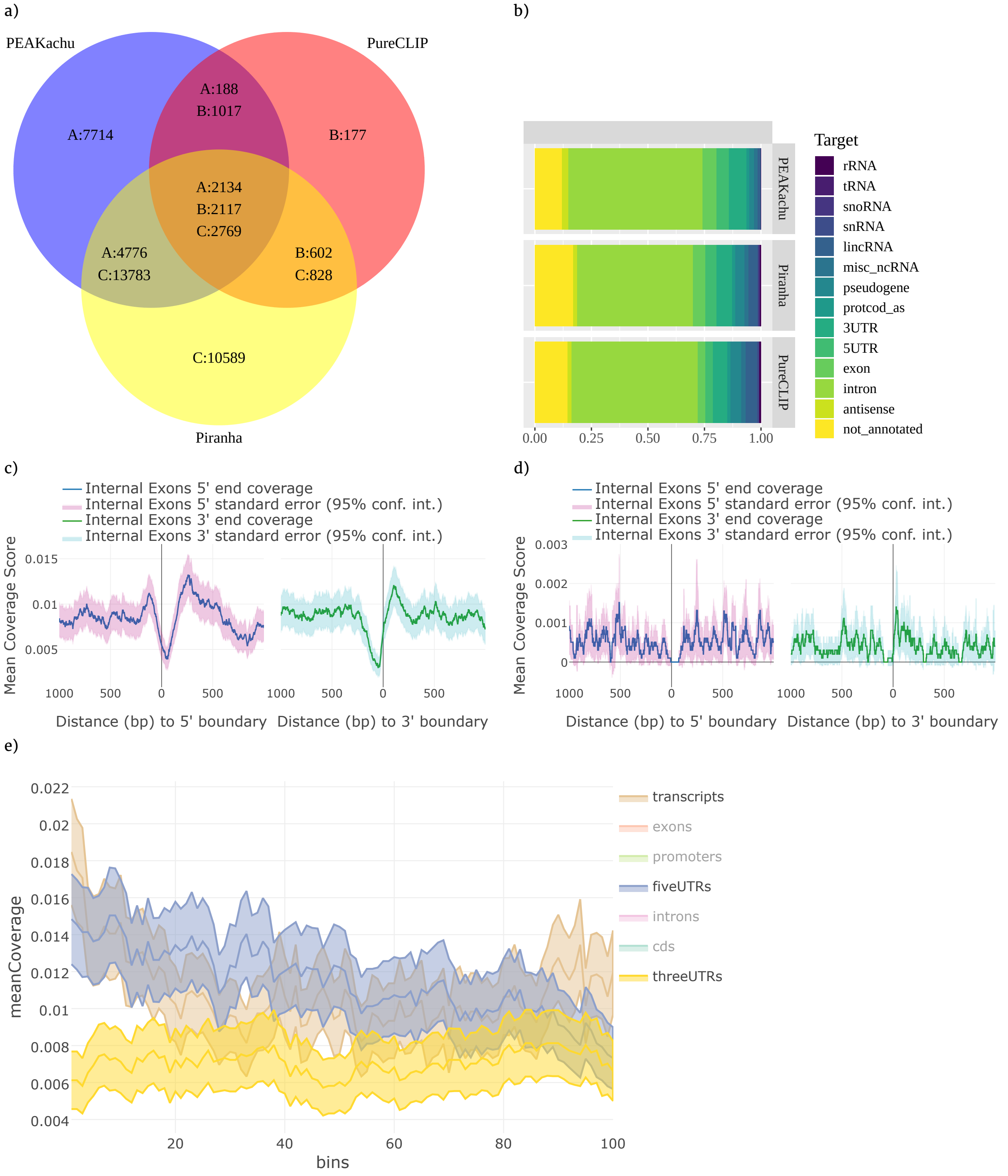

Figure 2. Comparison and analysis of the binding regions detected by PEAKachu, Piranha, and PureCLIP. The results are from the eCLIP data of RBFOX2 [13]. **(a)** We intersected the binding regions identified by the peakcallers with bedtools [39], paying attention to the strand (intersect -s), to appraise the robustness of each method. **(b)** We then annotated the binding regions of each peakcaller and plotted the fraction of each target. RBFOX2 prevalently binds introns, but also 3' and 5' UTRs as well as lincRNAs. The plot was generated with the hg19 script of targetdist [46]. Investigating the mean coverage of these binding regions identified by **(c)** PEAKachu [20] and **(d)** PureCLIP reveals an occupancy drop around the 5' and 3' ends of the exons. **(e)** Looking further at the mean coverage of the binding regions identified by PEAKachu in the overall transcript as well as for the 5' and 3' UTRs, in general a higher occupancy can be seen at the 5' end of the total transcript and 5' UTR. The thickness of the ribbon around the mean coverage indicates the 95% confidence interval (mean +- standard error of the mean times 1.96). Each feature is divided into 100 bins of equal length, whereas features smaller than 100 bp are excluded [41].

Table 1. Top five RBFOX2 sequence motifs for each peakcaller identified by MEME-ChIP, based on the RBFOX2 eCLIP data.

Peakcaller and Number of Peaks	Motif	E-value	Frac. of Seq. with Motif [%]
PEAKachu with 14,812 peaks	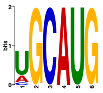	6.1e-702	37.21
	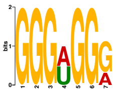	3.2e-075	30.91
	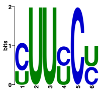	2.7e-049	22.19
	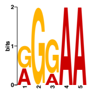	6.2e-045	33.57
	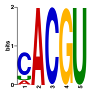	1.3e-027	7.44
Piranha with 27,969 peaks	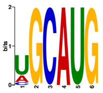	6.6e-381	16.43
	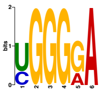	1.4e-065	16.50
	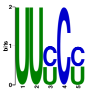	3.2e-057	18.30
	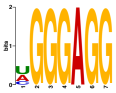	1.4e-038	10.32
	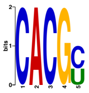	2.2e-031	7.57
PureCLIP with 3,913 peaks	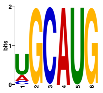	7.7e-288	40.20
	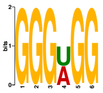	4.1e-030	27.17
	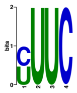	8.2e-009	18.43
	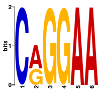	7.6e-006	2.79
	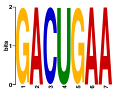	3.0e-005	0.97

tal number of peaks. PEAKachu on the other hand also has a high number of individual peaks, but significantly less than Piranha.

To identify the type of bound genomic regions, we annotate the peaks discovered by the three different peakcallers. The distributions of binding sites generated by these three tools

show a similar trend and prevalence for introns as the main target of RBFOX2 (Figure 2b). Yet, introns are not the only target. Our findings suggest that some binding regions of RBFOX2 lie in 3' and 5' UTRs, but this fraction is not as big as for introns. PEAKachu, Piranha and PureCLIP further detect another chunk of target sites in lincRNAs, but the portion detected by PEAKachu is smaller, probably because of a low coverage of lincRNAs in CLIP protocols.

To verify that RBFOX2 is a splicing factor, we investigate the peak profile of the binding sites by looking at the coverage plot of RBFOX2. Both PureCLIP and PEAKachu depict a drop in the binding region coverage around the exon-intron-boundaries at the 5' and 3' ends (Figure 2c and 2d). The drop is more intense for the sites found by PEAKachu. Checking the mean coverage of the binding regions called by PEAKachu (Figure 2e), the results suggest a binding prevalence of RBFOX2 in the upstream region of the transcripts. Furthermore, RBFOX2 seems to target the beginning of the 5' UTR. In contrast, the binding coverage is homogeneous for the 3' UTR. The sequence *UGCAUG* seems to play an important part for the binding of RBFOX2 as it is among the top five motifs detected by the PEAKachu, Piranha and PureCLIP pipeline (see Table 1). The second motif shows guanine richness and the third motif cytosine and uracil richness for all peakcallers.

At the end, we check the function of RBFOX2 to clarify the role in human liver cancer cells (Hep G2). Looking at the top hundred genomic regions that have RBFOX2 binding sites, we find Shank2 and Shank3 among the top hits as potential targets. Furthermore, a gene ontology (GO) analysis with RCAS for the targets of RBFOX2 identifies the protein to be relevant for the regulation of RNA splicing (with a Benjamini-Hochberg (BH) adjusted p-value of $< 10^{-4}$), as well as regulation of transcription by RNA polymerase I (BH adjusted p-value $< 10^{-3}$). In addition, the GO analysis identifies RBFOX2 to be involved in the regulation of histone modifications (BH adjusted p-value $< 10^{-4}$), nucleosome and nucleosomal binding (BH adjusted p-value $< 10^{-4}$ and 0.04, respectively) and methyl-CpG binding (BH adjusted p-value < 0.04).

Peak calling Is the Most Sensitive Analysis Step

Our results revealed that different peakcallers deliver greatly varying sets of binding sites. We analyzed the discrepancy between the pipeline of Nostrand et al. [13] with the CLIPper algorithm [21] and CLIP-Explorer in more detail, using detected motifs and preferred genomic binding locations as quality criteria. Our analysis indicates that the differences between CLIPper and PEAKachu (CLIP-Explorer) might result from the fact that CLIPper was called for each CLIP replicate separately, followed by a robust peak detection between the two peak sets. PEAKachu, on the other hand, was called with all replicates in mind. Consequently, PEAKachu (CLIP-Explorer) might profit from a better noise estimation between replicates and thus detects less false negatives. The results for SLBP endorse the conjecture, which showed a big difference in the number of peaks between CLIPper and CLIP-Explorer with PEAKachu. We found that roughly only 10% of the CLIPper peaks were contained in both replicates. This variation between replicates is in agreement with the literature [48].

To provide a wide range of peakcallers, we implemented and optimized three different peakcallers in CLIP-Explorer, namely Piranha, PEAKachu, and PureCLIP. Checking the results for RBFOX2, Piranha found the highest number of peaks. A lot of these peaks, however, might represent false positives since Piranha was executed without the information from the control experiments (see Methods). More than one third of Piranha's peaks are not included in PEAKachu's and PureCLIP's peak pool, which endorses the supposition. In contrast, PureCLIP had the lowest number of predicted peaks and the highest fractions of peaks shared with the other peakcallers. This indicates that PureCLIP selects the peaks based on very stringent criteria. However, PureCLIP might also have a high false negative rate, a problem likely shared with CLIPper. PureCLIP and CLIPper both call peaks for each replicate separately, which is likely the reason for missing some good candidates that are jointly found by PEAKachu and Piranha.

Despite the disparate number of peaks between PEAKachu, Piranha, and PureCLIP, the main motif of RBFOX2 with the sequence *UGCAUG* was identified with CLIP-Explorer for all used peakcallers. The motif seems to be very robust to varying peak calling conditions. The cytosine, uracil and guanine richness of the other motifs, found by all peakcallers, might be a property of the protocol or a representation of repetitive elements. CLIPper and CLIP-Explorer also had common motifs despite their different numbers of predicted binding regions. It is therefore possible to find a better ground truth, that is to say, a benchmark set with CLIP-Explorer, since more than one peakcaller can be tested with just a few clicks. A simple exchange with a different peakcaller is often not supported by other pipelines such as iCount [25].

Verifying Known Facts of RBFOX2

We further tried to verify RBFOX2's known role as splicing factor and that it preferably binds to introns [21, 27, 28, 29] to substantiate the credibility of CLIP-Explorer. The distributions of binding sites generated by PEAKachu, Piranha, and PureCLIP showed a prevalence for introns as the main target of RBFOX2 in accordance with the literature [28, 29]. Since Piranha might have too many false positives, we excluded Piranha from the subsequent binding coverage comparison. The drop of the binding occupancy of RBFOX2 around the exon-intron boundaries can also be seen in other studies [28, 29]. The binding coverage of RBFOX2 around the exon-intron-boundaries suggests an involvement of RBFOX2 in the regulation of splicing. The GO term analysis corroborates the hypothesis linking RBFOX2 to various splicing and structure-related processes, such as histone modifications. Besides, the Shank gene family members, as a potential target of RBFOX2, play an important part in neuronal functions, where alterations in the encoded proteins may be connected to autism [21]. Shank 2 and Shank 3 might hereby be regulated by alternative splicing [49, 50]. Another study has found the same interdependence of RBFOX2 and the Shank protein family [21].

Recommendations for PEAKachu, Piranha, and PureCLIP

To this day, a benchmarking dataset for CLIP-Seq data analysis does not exist because of missing experimental methods to verify predicted binding sites. It is therefore recommended to test more than one peakcaller and to test more than one parameter set, which is easily possible with CLIP-Explorer.

The newest version of PEAKachu [20] is well-suited for an experimental setup with at least two replicates for the CLIP experiment and at least two replicates for the control experiment, because it uses DESeq2 [51]. It is therefore best to turn on the DESeq2 normalization. If the user has less replicates, the user can still use PEAKachu, but DESeq2 requires at least two replicates for both experiment and control to calculate p-values. With less than two replicates, PEAKachu filters the peaks based on the fold change and the mad (median absolute deviation) multiplier. It is therefore wise to check the peaks with another peakcaller or peak calling pipeline. PEAKachu works best in the

adaptive mode with a mad multiplier of 0.0, a log2 fold change threshold of 2.0, and an adjusted p-value threshold of 0.05. PEAKachu needs the parameter of the maximum insert size, identified beforehand by Picard [52]. The estimation of the insert size is only necessary if the user provides paired-end read data. The window mode of PEAKachu is not recommended, because it is rather unstable. The mad multiplier might reduce the number of peaks, as it works as a second cutoff. To get the full peak pool, it is wise to leave it at zero and filter the peaks by the log2 fold change together with the adjusted p-value. One key parameter is the minimum block overlap that has to be tested with the default of 0.5 in the beginning. The user has to increase this parameter if the results show a lot of peaks in a close vicinity. Another critical parameter is the minimum cluster expression fraction and the minimum block expression. These parameters can change the total number of predicted peaks. Leave them in default with 0.01 and 0.1 and adjust the parameters if some interesting binding regions are not covered by PEAKachu's prediction. PEAKachu can be applied to iCLIP, eCLIP, FLASH, uvCLAP and even older protocols like PAR-CLIP.

Piranha [1] can be applied to an experimental setup with or without control, but it does not support replicates for the CLIP experiment. Each replicate has to be treated separately or further validated with a robust peak detection. However, if the user has only one replicate and no control, we recommend to use Piranha. If a control is provided, Piranha uses a zero-truncated negative binomial regression by default. Without a control it is wise to stick to a negative binomial. The distance to merge significant bins is one of the most crucial parameters of Piranha, similar to the minimum block overlap of PEAKachu. If the user observes a lot of peaks in a close vicinity, then this parameter has to be increased (e.g, 10). The bin size of the signal and control is another crucial parameter of Piranha that needs to be optimized. If the bin size is quite big (e.g., 200), then Piranha might miss a few good candidates. If the bin size is very small (e.g., 5), then Piranha predicts a lot of false positives. Piranha can also be applied to iCLIP, eCLIP, FLASH, uvCLAP, and even older protocols like PAR-CLIP.

PureCLIP [22] can be applied to an experimental setup with or without control, but it does not support replicates by the time of our analysis. It is therefore best to apply PureCLIP, as well as Piranha, to each replicate separately and find robust peaks by intersection, merging or calculating an IDR. Therefore, we recommend to use PureCLIP if the user has only one replicate for the CLIP and control experiment. PureCLIP already incorporates two default parameters sets. One set can be used if the protein is assumed to bind low complex motifs, which results in more broader and unspecific binding sites. PureCLIP predicts not only the binding region, but also the crosslinking sites. In our tests, PureCLIP quite often reported very small binding regions, almost identical to the crosslinking sites. It is therefore recommended to slightly extend the predicted binding sites (e.g, five to ten bases to the left and right) to cover the whole binding region. Furthermore, if the user provides paired-end reads, the mate containing the crosslinking event has to be provided explicitly. For iCLIP, FLASH, and uvCLAP this corresponds to the first mate, while for eCLIP it is the second mate. PureCLIP was specifically designed for eCLIP and iCLIP [22]. We recommend to use the peakcaller only for those protocols or other variants such as FLASH or uvCLAP.

It is not easy to find a standard peak calling algorithm with a standard parameter set. We tried to cover possible cases and recommendations for Piranha, PEAKachu, and PureCLIP, but these tools can change over time, or a new peakcaller might outrank them. The user can therefore find permanently updated recommendations and guidelines for a CLIP-Seq data analysis on CLIP-Explorer's main domain.

Potential implications

CLIP-Explorer is a valuable tool for researchers working with CLIP-Seq data as it simplifies and integrates many processing steps in a well-tested and optimized pipeline. CLIP-Explorer provides the user with an extensive overview of the potential function of the RBP and its target RNAs. It can be easily extended or modified, and has no installation overhead as it is integrated in Galaxy. CLIP-Explorer is thus the first general and fully automatic pipeline for eCLIP, FLASH, and uvCLAP data, which can also be applied to iCLIP and other types of CLIP-Seq protocols. Besides, it is permanently maintained, and new tools can be implemented or exchanged with existing ones to warrant a highly efficient data analysis.

We analyzed different eCLIP datasets and compared our findings with different databases [43, 44] and the results from the study by Nostrand et al. [13]. The analysis of different proteins, such as DROSHA, HNRNPK, and in more detail RBFOX2, showed the strength of the flexibility provided by CLIP-Explorer. We could identify similar and even new sequence motifs for PTBP1 with the motif *CAGGCUGG*, LIN28B with *CAGCCUG* and *CUCA*, U2AF2 with *ACAG*, DROSHA with *GGAGG*, QKI with *AGGCU*, and SLBP and KHDRBS1 with a handful of new motifs, which were found only by CLIP-Explorer. We also verified the sequence motif *UGCAUG* for RBFOX2 and found other guanine, cytosine and uracil-rich binding regions. The most significant sequence motif *UGCAUG* of RBFOX2 was found by Piranha, PEAKachu, and PureCLIP, even though the three peakcallers predicted three substantially different peak sets. Based on our results we recommend to test more than one peak calling algorithm for other RBPs to assess the robustness of the motifs. CLIP-Explorer allows this very easily, because of its user-friendly interface. An exchange of the peakcaller can be done in an instant. Furthermore, CLIP-Explorer enabled us to identify the involvement of RBFOX2 in splicing because of the availability of specific annotation tools like RCAS [41]. We also investigated the binding coverage of RBFOX2 over all transcripts with CLIP-Explorer. We showed a decreased RBFOX2 occupancy around the exon-intron boundaries, and a prevalence for the upstream region of the total transcripts and the beginning of the 5' UTRs. In addition, a GO term analysis linked RBFOX2 to the regulation of splicing and DNA structural modifications. We also found Shank2 and Shank3 as possible targets. Both proteins undergo alternative splicing and play an important part in neuronal development [49, 50]. All in all, CLIP-Explorer is a flexible and easily extendable pipeline, which greatly simplifies CLIP-Seq data analysis on a transcriptome- and genome-wide scale.

Methods

CLIP-Explorer includes all major processes that are required to analyze CLIP-seq data. The analysis involves three major steps as shown in Figure 1, each followed by a specific quality control. In the preprocessing step, the data is demultiplexed into the read libraries stemming from different experiments. If necessary, adapter sequences as well as in-line barcodes and UMIs are removed. FastQC [34] performs during that step a standard quality check for the read and library quality.

The postprocessing for CLIP experiments is similar to RNA-seq experiments, that is, the reads are aligned and filtered. An additional deduplication step is required in the case of recent CLIP-Seq protocols such as iCLIP and eCLIP. The deduplication removes PCR duplication artifacts. CLIP-Explorer performs another quality control during the postprocessing using mainly FastQC [34], and deeptools [40].

The final and major part of CLIP-Explorer is the analysis

step. CLIP-Explorer searches in that step for sequence and coverage motifs, and potential targets of the investigated RBP. Peak calling and motif detection are the fundamental and most critical processes. The quality and amount of detected binding sites can vary significantly based on the used tools, whereas the tools depend heavily on the experimental setup. Different tools therefore lead in part to different results. For that reason, CLIP-Explorer provides several peak calling and motif detection tools to fathom the robustness of the results.

Input and Output of CLIP-Explorer

The user needs to provide their experimental data in FASTA or FASTQ format. Nonstandard adapter sequences can be provided by the user. Otherwise, CLIP-Explorer automatically detects them. The pipeline is designed for multiplexed or demultiplexed paired read data and supports replicates and control experiments. Barcode sequences are required in case of demultiplexing. Other additional files are provided by the Galaxy database. CLIP-Explorer can be easily changed, for example, to allow for single end reads and different tool settings.

The user obtains a MultiQC [53] report for the raw, trimming, alignment, and deduplication quality to assess the quality of the raw data and important processing steps of CLIP-Explorer. MultiQC collects the FastQC reports made during the pre- and postprocessing of CLIP-Explorer to inspect the mapping quality, elaborating on the amount of unmapped and multiply mapped reads, the length of mapped reads, and other important characteristics of the read library. Further quality control is provided by deeptools, as it can elicit differences in signal and control experiments. A heatmap and a fingerprint plot assess the correlation between the signal and control libraries providing evidence for a correct execution of the CLIP experiment. CLIP-Explorer also generates coverage files (bigWig and bedGraph) for the alignments and the crosslinking sites to inspect the peak calling quality. Most importantly, CLIP-Explorer will produce a bed and gtf file of significantly enriched regions, representing the binding sites of the protein on the transcriptome or genome. A MEME-ChIP [42] report will further analyze the peaks, detecting potential sequence motifs of the protein. A FIMO [42] report then lists reference sequences, which where not covered by the peak calling, but contain the detected sequence motifs. Finally, a RCAS (RNA centric annotation system) [41] report determines the target distribution of the protein over RNA classes and transcript regions. It also includes a GO term analysis and plots to fathom the coverage of the protein binding around splice junctions, along the transcripts and along various other regions. CLIP-Explorer can also generate a list of robust peaks (shared between all input files). This feature is useful for peakcallers that do not support replicated data such as Piranha.

Mapping and Deduplication

We integrated STAR [35] into CLIP-Explorer to map reads against the genome, using extra information about the transcriptome. The data of RBFOX2 was mapped against hg19 to better reproduce the literature results, all other proteins such as DROSHA and HNRNPK were mapped against hg38. STAR was executed with the *two pass mode* turned on and in the end-to-end alignment scheme. CLIP-Explorer further checks for incomplete pairs, ambiguously mapped and low quality reads. CLIP-Explorer also includes a deduplication step to lower the false positive rate for the identification of binding regions. PCR duplicates are often collapsed into one representative [2, 19]. CLIP-Explorer identifies potential PCR duplicates with the help of UMI-tools [31]. Duplicated reads are identified after the alignment step, searching for reads with identical genomic positions (begin and end) and orientation. Yet, sequencing errors can also occur in the UMIs. UMI-tools clusters the reads based on their UMI to handle these sequencing errors. Thus, we merged sequences with a high node count and a small Hamming distance between unique UMIs [31].

Identification of Enriched Regions and Sequence Motifs

Searching for enriched regions and motifs is the most challenging task because of the differences in the gene expression between CLIP experiments and background controls. Hence, high false negative rates are a common result in the detection of differentially enriched regions (peaks) [19]. CLIP-Explorer allows to choose between three different peakcallers, namely PEAKachu [20], PureCLIP [22], and Piranha [1]. PEAKachu was executed in adaptive mode with a mad multiplier of 0.0, a log2 fold change threshold of 2.0, an adjusted p-value (Benjamini-Hochberg procedure) threshold of 0.05 and a maximum insert size of 200, identified beforehand by Picard. All other parameters are set to their default values. We used for Piranha a negative binomial distribution with a bin size of 20 and a 0.05 p-value threshold. We did not include the control data for Piranha to test for possible experiments without control datasets. PureCLIP was trained on chromosome one, two, and three of hg38 (for RBFOX2 we used hg19, respectively), and executed with *-bc 0* as the default option. The resulting binding regions of PEAKachu, Piranha, and PureCLIP were analyzed with RCAS [41] to determine the target distribution over genomic regions and possible binding patterns of RBFOX2. The peaks were also analyzed with the MEME Suite [42] tool package (MEME-ChIP) to find sequence motifs in the peaks. MEME-ChIP was set to find zero or one occurrence of the motif sites per sequence (zoops model).

Intersecting Peaks

We used the intersect module of bedtools [39] to assess the occurrence of the predicted peaks between CLIPper and CLIP-Explorer with PEAKachu, and between the three different CLIP-Explorer pipelines with Piranha, PureCLIP, and PEAKachu. We set bedtools intersect with the option *-s* to search for intersections on the same strand and kept the default value for *-f*, resulting in a minimum overlap of one base for overlapping regions to be reported. Further, we used the flag *-u* to consider only unique overlaps.

Availability of supporting source code and requirements

Project name: CLIP-Explorer
Project home page: https://clipseq.usegalaxy.eu/
Operating system(s): Galaxy
Training material: https://galaxyproject.github.io/training-material/topics/transcriptomics/tutorials/clipseq/tutorial.html

Availability of supporting data and materials

CLIP-Explorer provides a small dataset for a test run, which can be found in the training material and on the CLIP-Explorer website. The whole eCLIP data used in this paper, such as RBFOX2 or PTBP1, is listed in the supplementary of the study by Nostrand et al. [13].

Additional files

Supplementary Table 1. Top five DREME sequence motifs of MEME-ChIP [42] of Different Proteins. CLIP-Explorer's sequence logos of different proteins from the binding regions that were identified by PEAKachu. Furthermore, sequence motifs of MEME-ChIP from the binding regions that were identified by the CLIPper algorithm [21]. The sequence motifs of CLIP-Explorer and CLIPper originated from eCLIP data [13]. To compare the sequence logos other motifs were collected from different databases [43, 44].

Supplementary Table 2. Peak intersections between PEAKachu [20] of CLIP-Explorer and CLIPper from the the study by Nostrand et al. [13]. A list of Venn diagrams showing the overlap between PEAKachu [20] (CLIP-Explorer) and CLIPper peaks [13].

Declarations

List of abbreviations

CLIP-Seq: Crosslinking immunoprecipitation in combination with high-throughput sequencing; RBP: RNA-binding proteins.

Ethical Approval

Not applicable

Consent for publication

Not applicable

Competing Interests

The authors declare that they have no competing interests.

Funding

This study was supported by the German Research Foundation (DFG) under Germany's Excellence Strategy (CIBSS – EXC-2189 – Project ID 390939984), DFG grant BA2168/11-2 SPP 1738, DFG grant TRR 167/1 2027 NeuroMac, DFG grant GRK 2344/1 2017 MeInBio - BioInMe Research Training Group, and by the Collaborative Research Centre 992 Medical Epigenetics (DFG grant SFB 992/2 2016).

Author's Contributions

F.H. and D.M. performed the computational analysis. R.B., and D.M. initialized the project, and supervised the research. F.H. and R.B. wrote the manuscript with inputs from other authors. All authors read and approved the final manuscript.

Acknowledgements

We are grateful to the members of the Galaxy team Freiburg (http://www.bioinf.uni-freiburg.de/Galaxy/) and to Torsten Houwaart for his support.

References

1. Uren PJ, Bahrami-Samani E, Burns SC, Qiao M, Karginov FV, Hodges E, et al. Site identification in high-throughput RNA-protein interaction data. Bioinformatics 2012;28(23):3013–3020.
2. Chakrabarti AM, Haberman N, Praznik A, Luscombe NM, Ule J. Data Science Issues in Understanding Protein-RNA Interactions. bioRxiv 2017;.
3. Hentze MW, Castello A, Schwarzl T, Preiss T. A brave new world of RNA-binding proteins. Nature Reviews Molecular Cell Biology 2018;19:327–341.
4. Baltz AG, Munschauer M, Schwanhäusser B, Vasile A, Murakawa Y, Schueler M, et al. The mRNA-bound proteome and its global occupancy profile on protein-coding transcripts. Molecular cell 2012;46(5):674–690.
5. Castello A, Fischer B, Eichelbaum K, Horos R, Beckmann BM, Strein C, et al. Insights into RNA Biology from an Atlas of Mammalian mRNA-Binding Proteins. Cell 2012;149(6):1393–1406.
6. Gerstberger S, Hafner M, Tuschl T. A census of human RNA-binding proteins. Nature Reviews Genetics 2014;15(12):829–845.
7. Beckmann BM, Castello A, Medenbach J. The expanding universe of ribonucleoproteins: of novel RNA-binding proteins and unconventional interactions. Pflügers Archiv-European Journal of Physiology 2016;468(6):1029–1040.
8. Pereira B, Billaud M, Almeida R. RNA-Binding Proteins in Cancer: Old Players and New Actors. Trends in cancer 2017;3(7):506–528.
9. Nussbacher JK, Batra R, Lagier-Tourenne C, Yeo GW. RNA-binding proteins in neurodegeneration: Seq and you shall receive. Trends in neurosciences 2015;38(4):226–236.
10. Jankowsky E, Harris ME. Specificity and nonspecificity in RNA-protein interactions. Nature reviews Molecular cell biology 2015;16(9):533–544.
11. Hafner M, Landthaler M, Burger L, Khorshid M, Hausser J, Berninger P, et al. Transcriptome-wide Identification of RNA-Binding Protein and MicroRNA Target Sites by PAR-CLIP. Cell 2010;141(1):129–141.
12. Huppertz I, Attig J, D'Ambrogio A, Easton LE, Sibley CR, Sugimoto Y, et al. iCLIP: Protein-RNA interactions at nucleotide resolution. Methods 2014;65(3):274–287.
13. Van Nostrand EL, Pratt GA, Shishkin AA, Gelboin-Burkhart C, Fang MY, Sundararaman B, et al. Robust transcriptome-wide discovery of RNA-binding protein binding sites with enhanced CLIP (eCLIP). Nature methods 2016;13(6):508.
14. Lee FC, Ule J. Advances in CLIP technologies for studies of protein-RNA interactions. Molecular cell 2018;69(3):354–369.
15. Sugimoto Y, König J, Hussain S, Zupan B, Curk T, Frye M, et al. Analysis of CLIP and iCLIP methods for nucleotide-resolution studies of protein-RNA interactions. Genome biology 2012;13(8):R67.
16. König J, Zarnack K, Rot G, Curk T, Kayikci M, Zupan B, et al. iCLIP reveals the function of hnRNP particles in splicing at individual nucleotide resolution. Nature structural & molecular biology 2010;17(7):909–915.
17. Wheeler EC, Van Nostrand EL, Yeo GW. Advances and challenges in the detection of transcriptome-wide protein-RNA interactions. Wiley Interdisciplinary Reviews: RNA 2018;9(1):e1436.
18. Maticzka D, Ilik IA, Aktas T, Backofen R, Akhtar A. uvCLAP is a fast and non-radioactive method to identify in vivo targets of RNA-binding proteins. Nature communications 2018;9(1):1142.
19. Uhl M, Houwaart T, Corrado G, Wright PR, Backofen R. Computational analysis of CLIP-seq data. Methods

2017;118:60–72.
20. Bischler T, Maticzka D, Förstner KU, Wright PR, PEAKachu;. https://github.com/tbischler/PEAKachu.
21. Lovci MT, Ghanem D, Marr H, Arnold J, Gee S, Parra M, et al. Rbfox proteins regulate alternative mRNA splicing through evolutionarily conserved RNA bridges. Nature structural & molecular biology 2013;20:1434.
22. Krakau S, Richard H, Marsico A. PureCLIP: capturing target-specific protein–RNA interaction footprints from single-nucleotide CLIP-seq data. Genome biology 2017;18(1):240.
23. Chen B, Yun J, Kim MS, Mendell JT, Xie Y. PIPE-CLIP: a comprehensive online tool for CLIP-seq data analysis. Genome biology 2014;15(1):R18.
24. Maragkakis M, Alexiou P, Nakaya T, Mourelatos Z. CLIPSeqTools—a novel bioinformatics CLIP-seq analysis suite. RNA 2016;22(1):1–9.
25. Curk T, Rot G, Gorup u, Zmrzlikar J, Konig J, Sugimoto Y, et al. iCount: protein-RNA interaction iCLIP data analysis 2016;.
26. Afgan E, Baker D, Batut B, van den Beek M, Bouvier D, Čech M, et al. The Galaxy platform for accessible, reproducible and collaborative biomedical analyses: 2018 update. Nucleic acids research 2018;46(W1):W537–W544.
27. Gehman LT, Meera P, Stoilov P, Shiue L, O'Brien JE, Meisler MH, et al. The splicing regulator Rbfox2 is required for both cerebellar development and mature motor function. Genes & development 2012;.
28. Yeo GW, Coufal NG, Liang TY, Peng GE, Fu XD, Gage FH. An RNA code for the FOX2 splicing regulator revealed by mapping RNA-protein interactions in stem cells. Nature Structural and Molecular Biology 2009;16(2):130.
29. Singh RK, Xia Z, Bland CS, Kalsotra A, Scavuzzo MA, Curk T, et al. Rbfox2-Coordinated Alternative Splicing of Mef2d and Rock2 Controls Myoblast Fusion during Myogenesis. Molecular cell 2014;55(4):592–603.
30. Girardot C, Scholtalbers J, Sauer S, Su SY, Furlong EE. Je, a versatile suite to handle multiplexed NGS libraries with unique molecular identifiers. BMC bioinformatics 2016;17(1):419.
31. Smith TS, Heger A, Sudbery I. UMI-tools: Modelling sequencing errors in Unique Molecular Identifiers to improve quantification accuracy. Genome research 2017;.
32. Maticzka D, bctools;. https://github.com/dmaticzka/bctools.
33. Martin M. Cutadapt removes adapter sequences from high-throughput sequencing reads. EMBnet journal 2011;17(1):pp–10.
34. Andrews S, et al. FastQC: a quality control tool for high throughput sequence data 2010;.
35. Dobin A, Davis CA, Schlesinger F, Drenkow J, Zaleski C, Jha S, et al. STAR: ultrafast universal RNA-seq aligner. Bioinformatics 2013;29(1):15–21.
36. Langmead B, Salzberg SL. Fast gapped-read alignment with Bowtie 2. Nature methods 2012;9(4):357.
37. Hoffmann S, Otto C, Kurtz S, Sharma CM, Khaitovich P, Vogel J, et al. Fast mapping of short sequences with mismatches, insertions and deletions using index structures. PLoS computational biology 2009;5(9):e1000502.
38. Wysoker A, Fennell T, Ruan J, Homer N, Marth G, Abecasis G, et al. The Sequence alignment/map (SAM) format and SAMtools. Bioinformatics 2009;25:2078–2079.
39. Quinlan AR, Hall IM. BEDTools: a flexible suite of utilities for comparing genomic features. Bioinformatics 2010;26(6):841–842.
40. Ramírez F, Ryan DP, Grüning B, Bhardwaj V, Kilpert F, Richter AS, et al. deepTools2: a next generation web server for deep-sequencing data analysis. Nucleic acids research 2016;44(W1):W160–W165.
41. Uyar B, Yusuf D, Wurmus R, Rajewsky N, Ohler U, Akalin A. RCAS: an RNA centric annotation system for transcriptome-wide regions of interest. Nucleic acids research 2017;45(10):e91–e91.
42. Bailey TL, Boden M, Buske FA, Frith M, Grant CE, Clementi L, et al. MEME SUITE: tools for motif discovery and searching. Nucleic acids research 2009;37(suppl_2):W202–W208.
43. Giudice G, Sánchez-Cabo F, Torroja C, Lara-Pezzi E. ATtRACT – a database of RNA-binding proteins and associated motifs. Database 2016;2016.
44. Ray D, Kazan H, Cook KB, Weirauch MT, Najafabadi HS, Li X, et al. A compendium of RNA-binding motifs for decoding gene regulation. Nature 2013;499(7457):172.
45. Zhao H, Sun Z, Wang J, Huang H, Kocher JP, Wang L. CrossMap: a versatile tool for coordinate conversion between genome assemblies. Bioinformatics 2013;30(7):1006–1007.
46. Maticzka D, targetdist; 2016. https://github.com/dmaticzka/targetdist.git.
47. Wilbert ML, Huelga SC, Kapeli K, Stark TJ, Liang TY, Chen SX, et al. LIN28 binds messenger RNAs at GGAGA motifs and regulates splicing factor abundance. Molecular cell 2012;48(2):195–206.
48. Jungkamp AC, Stoeckius M, Mecenas D, Grün D, Mastrobuoni G, Kempa S, et al. In vivo and transcriptome-wide identification of RNA binding protein target sites;.
49. Guilmatre A, Huguet G, Delorme R, Bourgeron T. The emerging role of SHANK genes in neuropsychiatric disorders. Developmental neurobiology 2014;74(2):113–122.
50. Leblond CS, Heinrich J, Delorme R, Proepper C, Betancur C, Huguet G, et al. Genetic and functional analyses of SHANK2 mutations suggest a multiple hit model of autism spectrum disorders. PLoS genetics 2012;8(2):e1002521.
51. Love MI, Huber W, Anders S. Moderated estimation of fold change and dispersion for RNA-seq data with DESeq2. Genome biology 2014;15(12):550.
52. broadinstitute, Picard;. http://broadinstitute.github.io/picard.
53. Ewels P, Magnusson M, Lundin S, Käller M. MultiQC: summarize analysis results for multiple tools and samples in a single report. Bioinformatics 2016;32(19):3047–3048.

Supplements_Table_1

Click here to access/download
Supplementary Material
Supplements_1.pdf

Supplements_Table_2

Click here to access/download
Supplementary Material
Supplements_2.pdf

style

Click here to access/download
Supplementary Material
oup-contemporary.cls

bib

Click here to access/download
Supplementary Material
paper-refs.bib

bib style

Click here to access/download
Supplementary Material
vancouver-authoryear.bst

Manuscript tex

Click here to access/download
Supplementary Material
main.tex
