# Supplementary material for: Galaxy CLIP-Explorer: a web server for CLIP-Seq data analysis
Source: Gigascience. 2020 Nov 11;9(11):giaa108. doi: 10.1093/gigascience/giaa108 (PMC7657819; doi:10.1093/gigascience/giaa108)
Supplement: giaa108_GIGA-D-19-00287_Revision_1 [file giaa108_giga-d-19-00287_revision_1.pdf]

GigaScience

Galaxy CLIP-Explorer: a web server for CLIP-Seq data analysis

--Manuscript Draft--

**Manuscript Number:**	GIGA-D-19-00287R1
**Full Title:**	Galaxy CLIP-Explorer: a web server for CLIP-Seq data analysis
**Article Type:**	Review
**Funding Information:**	Deutsche Forschungsgemeinschaft (322977937/GRK2344 2017 MeInBio -- BioInMe) — Mr. Florian Heyl<br>Deutsche Forschungsgemeinschaft (BA2168/11-2 SPP 1738) — Mr. Michael Uhl<br>Deutsche Forschungsgemeinschaft (TRR 167/1 2027 NeuroMac) — Dr. Rolf Backofen<br>Deutsche Forschungsgemeinschaft (SFB 992/2 2016) — Dr. Rolf Backofen
**Abstract:**	Background:<br><br>Post-transcriptional regulation via RNA-binding proteins (RBP) plays a fundamental role in every organism, but the regulatory mechanisms lack important understanding. Nevertheless, they can be fathomed by crosslinking immunoprecipitation in combination with high-throughput sequencing (CLIP-Seq). CLIP-Seq answers questions about the functional role of an RBP and its targets by determining binding sites on a nucleotide level and associated sequence and structural binding patterns. In recent years the amount of CLIP-Seq data skyrocketed, urging the need for an automatic data analysis that can deal with different experimental setups. However, noncanonical data, new protocols, and a huge variety of tools, especially for peak calling, made it difficult to define a standard.<br><br>Findings:<br><br>CLIP-Explorer is a flexible, and reproducible data analysis pipeline for iCLIP data that supports for the first time eCLIP, FLASH, and uvCLAP data. Individual steps like peak calling can be changed to adapt to different experimental settings. We validate CLIP-Explorer on eCLIP data, finding similar or nearly identical motifs for various proteins in comparison with other databases. In addition, we detect new sequence motifs for PTBP1, and U2AF2. Finally, we optimize the peak calling with three different peakcallers on RBFOX2 data, discuss the difficulty of the peak calling step and give advice for different experimental setups.<br><br>Conclusion:<br><br>CLIP-Explorer finally fills the demand for a flexible CLIP-Seq data analysis pipeline that is applicable to the up-to-date CLIP protocols. The paper further shows the limitations of current peak calling algorithms and the importance of a robust peak detection.
**Corresponding Author:**	Florian Heyl<br><br>GERMANY
**Corresponding Author Secondary Information:**	
**Corresponding Author's Institution:**	
**Corresponding Author's Secondary Institution:**	
**First Author:**	Florian Heyl
**First Author Secondary Information:**	
**Order of Authors:**	Florian Heyl

Powered by Editorial Manager® and ProduXion Manager® from Aries Systems Corporation

	Daniel Maticzka
	Michael Uhl
	Rolf Backofen
**Order of Authors Secondary Information:**	
**Response to Reviewers:**	\section{Reviewer \#1}<br><br>The authors proposed a new web server for CLIP-seq data analysis. Although the work is sound and interesting, I have few suggestions/concerns that might improve the work.<br><br>Major revisions:<br>The authors propose specific tools for each step of the pipeline, however they missed to explain why they made this choice. The authors missed to cite recent CLIP-seq tools benchmark and to put their work in relationship with these previous related papers.<br><br>\begin{answer}<br>The use of Cutadapt for adapter trimming is nearly a standard for NGS data. Trim Galore is another tool, but it is also using Cutadapt in the background, and the adapter trimming cannot be turned off, which might be useful if the user has data where the adapters were already removed. Other tools like Trimmomatic are also supported by Galaxy and can be used instead of Cutadapt. We assume that a comprehensive adapter trimming method benchmark has not been done yet since the only real difference between them is the runtime, because adapter trimming is a step which can be easily checked. However, we put a short statement into the method section, where we cite a paper comparing the runtime of different adapter trimming methods, where Cutadapt outperformed the rest.<br><br>The use of UMI-tools is based on the fact that it is the only tool that can select UMIs taking possible sequencing errors into account, which might occur in the UMIs.<br><br>We put a statement into the methods section (Mapping and Deduplication) for the mapping with STAR. We integrated STAR into CLIP-Explorer because of the good performance and usability of STAR for RNA-Seq data. STAR is an annotation and splice aware aligner, which is important for transcriptomics data. We cited three benchmark papers, where STAR was compared with other read mappers for RNA-Seq data.<br><br>The use of MEME-ChIP for sequence motif finding results from its versatility and performance for known motifs. It is a combinatorial approach of an enumeration method (DREME) and a probabilistic model (MEME). Furthermore, it encompasses a module for local enrichments in the sequence motifs (CentriMo) and a module for the detection of secondary sequence motifs (SpaMo). Because of a missing ground truth (missing experimental methods to verify predicted motifs) for CLIP-Seq data, a benchmarking for motif finding tools is still missing. We put a statement into the methods section (Identification of Enriched Regions and Sequence Motifs).<br><br>The use of RCAS is also based on the versatility of the tool and the performance with RBFOX2 and other proteins. The tool simply collects the information obtained from the peak regions and runs a broad analysis of certain features that can then be used to imply biological functions. It is the only tool so far that can do such an extensive analysis for RNA-Seq related data.<br><br>The rest of quality control tools are either standard in any NGS data analysis (e.g., FastQC) or are short scripts to collect important features of CLIP-Seq experiments (e.g, bctools).<br><br>We also state now in the method section a selection of review articles for the analysis of CLIP-Seq data.<br>\end{answer}<br><br>Furthermore the benchmark of peak calling tools should be extended, including exact metrics to evaluate the comparisons of the performances of the selected tools.

	\begin{answer}<br>We do think that a statement that a peakcaller is better or worse is not appropriate because of a missing ground truth. We show and included now more metrics for the reader and potential user to give an idea about the characteristics of the different peakcallers, thus the user can make a sophisticated choice for their type of data. For that reason, we put a new Table and new results into the supplements of the paper (Supplementary Table 3 and 4).<br><br>The new Table compares all four peakcallers (PEAKachu, Piranha, PureCLIP, and CLIPper) regarding potential CLIP-Seq artifacts and biases for the RBFOX2 example data of the paper. The table shows the percentage of peaks that have the stated feature. The table describes and tries to answer several questions. (1) How many peaks do overlap with other peaks of the same peakcaller, i.e., how many peaks deliver unique information? (2,3) How many peaks come from the plus and how many peaks come from the minus strand, i.e., are there any potential mispriming events in the read libraries or does the peakcaller have a strand bias? (4,5) How many peaks overlap with 3'UTRs and how many peaks overlap with 5'UTRs, i.e., is there a protocol bias (e.g., restriction enzyme bias) or peakcaller problem that leads to an unusual amount of peaks in these regions? We tested further the specificity of the peakcallers, since the number of peaks between the different tools vary. We therefore checked (6) how many peaks overlap with repeat regions, (7) especially with intron repeats, and how many peaks overlap with pseudogenes of (8) ncRNA, (9) rRNA, and (10) tRNA, like reviewer 2 has suggested. The results are written in section "Effects of Using Different Peakcallers for RBFOX2".<br><br>We have done almost the same thing for the protein SLBP (Supplementary Table 4), because of the suggestion of reviewer 2. We also checked the number of peaks overlapping with histone genes and their UTRs, because SLBP targets mainly histone RNAs. The results are written in the section "Comparison of CLIP-Explorer's Results".<br><br>We also checked the peak length and the distance between the peaks of the different peakcallers for the RBFOX2 data (Supplementary Figure 3). We put the results in the section "Effects of Using Different Peakcallers for RBFOX2". We cannot make a general judgement about a good value for the peak length, because each RBP and each binding region can be specific and a benchmarking dataset is missing for CLIP-Seq data. Furthermore, the distances between the peaks are almost identical between the peakcallers PEAKachu, PureCLIp and CLIPper. Only Piranha has slightly more peaks that are closer together, indicating that Piranha might have many false positives because it calls many peaks (local maxima) in close proximity and does not combine them into a bigger peak (global maximum).<br>\end{answer}<br><br>Another key question that the authors should answer is how the pre-processing affect the peak caller tools? CLIP-explorer use 3 peak caller tools to call peaks, what would change running these stand alone tools compared to running them within CLIP-explorer?<br><br>\begin{answer}<br>We checked the behavior of the different peakcallers (PEAKachu, Piranha, and PureCLIP) based on a different pre-processing. We took the hg19 alignments of RBFOX2 (ENCFF154BQS, ENCFF994WPX, ENCFF590UCY) from ENCODE of the pipeline from the Nostrand et al. paper and ran the peakcallers on that data. We put the results in section "Effects of Using Different Peakcallers for RBFOX2".<br><br>The full main motif of RBFOX2 with the sequence \textit{UGCAUG} was identified only by PureCLIP. With CLIP-Explorer all three peakcallers found the main motif with lower background noise. Furthermore the motif set from CLIP-Explorer looks more similar between all three peakcallers.<br><br>\end{answer}<br><br>The choice of the multiple datasets used in the manuscript should be clearly stated in the methods or results section. Possibly a summary table would help the reader to keep track of the different analysis and dataset used.

Powered by Editorial Manager® and ProduXion Manager® from Aries Systems Corporation

\begin{answer}
We made a short overview paragraph in the methods section called "Analyzed Data".
\end{answer}

Overall the manuscript require a reorganization of the different sections and it is often redundant and repetitive. For instance the peak callers comparison should be mentioned in the first place in order to select the "best" tool to use in afterwards within the galaxy framework. Then how the pre-processing affect the peak calling step, in order to show (or not) the advantages to use the peak callers within CLIP-explorer respect to the stand alone version. Finally the results of the application of CLIP-explorer on several dataset.

\begin{answer}
We have rearranged and pruned the paper.
\end{answer}

Another concern regards the flexibility of CLIP-explorer: the authors state in the manuscript the CLIP-seq analysis tools change over time and new tools that outrank the chosen ones may happen. How the authors would face this challenge? How flexible is CLIP-explorer to include new tools, change parameters etc?

\begin{answer}
The flexibility of CLIP-Explorer can be seen and already tested with Galaxy. A tutorial or description of Galaxy would be beyond the scope of the paper (see https://galaxyproject.github.io/training-material/). However, tools and tool versions can be easily exchanged in CLIP-Explorer (see https://galaxyproject.github.io/training-material/topics/introduction/tutorials/galaxy-intro-101/tutorial.html for a tutorial about Galaxy's workflows). It is a simple click to remove and a new click to add a new tool. The new tool can be connected to the workflow with simple drag and drop actions. All important tool parameters are well described and the user can change them without the knowledge of any programming language or the use of the terminal. Figuratively, the user can use bioinformatic tools in an environment that is more suited for a reproducible and user-friendly data analysis.
\end{answer}

Minor revision:

Table 1 is not easy readable and takes a lot of space. Perhaps it would be better having the peak callers on the columns instead of the rows, this would allow easier comparisons of motifs.

\begin{answer}
The layout of Table 1 was improved to make it more readable and concise. It also includes now the peakcaller results for a different data pre-processing.
\end{answer}

The paragraph "verifying known facts of RBFOX2" goes beyond the aims of the paper and lead no strong conclusions to support the original question.

\begin{answer}
We moved the text into section "Effects of Using Different Peakcallers for RBFOX2" and adapted the text slightly to fit the content better. We think it is still relevant to show the versatility and credibility of CLIP-Explorer. We understand that the paper is not supposed to show any highly biological relevance to RBFOX2, but we reckon as a proof of concept, that is to say, we find the same observation as stated in previous studies, showing what CLIP-Explorer is capable of.
\end{answer}

Powered by Editorial Manager® and ProduXion Manager® from Aries Systems Corporation

\section{Reviewer \#2}

The manuscript by Heyl et al describe a data analysis pipeline for processing CLIP-seq data that enables simplified comparison between peak callers and analysis options using the Galaxy server and structure. The development of improved (and standardized) CLIP-seq peak calling and analysis tools is of great utility to the CLIP field, and CLIP-explorer seems like it has appealing characteristics as a Galaxy implementation that will extend usability to non-experts (and the extensive tutorial and documentation provided in the associated Galaxy pipeline are well-done). As Gigascience has open reviews, I can note here that I am one of the primary contributors to the ENCODE eCLIP resource.

I have two main areas of concern with the current manuscript:

First, with respect to the analysis of CLIPper peaks provided by the ENCODE consortium:

$>>$In addition, CLIPper sometimes misses important motifs in comparison to CLIP-Explorer. CLIPper does not find the known motif UGCAUG of RBFOX2 [21, 28, 29] and GGAGA of LIN28B [47] (Supplements: Table 1).

With respect to the authors, I think there may be some confusion in the analysis here, as this is in direct contradiction to the original eCLIP paper (PMID 27018577; Supplementary Figures 3F discussing an RBFOX2 293T dataset) and my experience with the ENCODE data (and the RBFOX2 datasets in particular).

I do not see in the Methods section any discussion of significance (p-values and/or fold-enrichment in IP versus input) - the eCLIP paper discussed that (CLIPper-identified) peaks that were not significantly enriched in IP versus input did not contain the RBFOX2 motif and should not be included in downstream analysis, but I believe (in many sections in the manuscript) that the authors use all CLIPper-identified clusters rather than only the subset that are significantly enriched (based on the \# of peaks listed in the text).

I also am not clear if motif analysis was performed on the CLIPper peaks only, or whether this analysis also included flanking regions (this is not mentioned in the text, though in the associated galaxy tutorial there is a step that adds 20nt to each side of peaks prior to motif analysis, but I am unclear if this was performed for CLIPper peaks as well).

Although I am not as familiar with the MEME-ChIP software the authors use, when I run that software on the CLIPper peaks from the RBFOX2 dataset (specifically ENCFF639MYI.bed , which is the RBFOX2 HepG2 replicate 1 peak bed file):

MEME-ChIP -o /outputdirectory/ -dna -seed 123 -filter-thresh 0.05 -norc -meme-mod zoops -meme-minw 5 -meme-maxw 20 -meme-nmotifs 20 -dreme-e 0.05 -dreme-m 5 ENCFF639MYI.bed.fa

I agree that there is no visible RBFOX2 motif enriched (attached image)

However, as the authors note, many peak callers (including CLIPper) often call peaks that terminate prior to the motif itself (due to reverse transcription termination within the motif, which causes the read density to drop within or 3' of the motif). If the CLIPper peaks are extended by 10nt on the 5' end only, the canonical RBFOX motif then becomes more clearly enriched (attached image)

More critically, however, if this is done using IP versus input enrichment cutoffs recommended in the ENCODE papers (log2(foldenrichment) $>=$ 3 and -log10(p-value) $>=$ 3), the RBFOX2 motif is recovered even without this 10nt extension (attached image)

And becomes more clearly enriched with the 10nt extension (attached image)

Particularly as the authors are comparing peak callers that include an input normalization step, it seems more appropriate to use the input-normalized CLIPper peaks for comparative analysis here.

\begin{answer}
For the analysis in the manuscript, we took the peaks from the IDR (signal normalization), for example, RBFOX2 with ENCFF154DRN (hg19). However indeed, we performed the motif analysis on the CLIPper peaks only without the inclusion of the flanking regions. We assumed that this was already done to the CLIPper peaks. Therefore, we repeated the analysis including a 10nt extension. Furthermore, we found a bug in the CrossMap tool (for hg19 to hg38 or vice versa genomic location conversion) and fixed it. We can therefore verify the observations of reviewer 2 and made the appropriate changes.

Thus, the Supplements 1 and 2 and the results in section "Comparison of CLIP-Explorer Results" have changed.
\end{answer}

My other concern with the manuscript as written is that a lot of emphasis describes differences (or similarities) in CLIP-explorer output versus other peak callers, but the discovery of additional peaks and novel features (motifs, binding patterns, etc) are often assumed as positive and unique insights rather than as potential evidence of false-positive signal. As the main purpose of this manuscript is not a deep comparison of peak callers but rather the implementation of the CLIP-Explorer pipeline, I think it would be more appropriate to present both of these options except where some independent validation can be described.

\begin{answer}
Thank you very much for this point. It is true that we did not aim at a deep comparison, but tried to sensitize for possible differences in the results analyzing CLIP-Seq data with different peakcallers. We therefore state now clearly and more than once in the paper, because of a missing benchmarking set (ground truth) it is not guaranteed that each peakcaller works impeccable, that is to say, all peakcallers (PEAKachu, Piranha, PureCLIP, CLIPper) might have false positives and false negatives. We mention this in section "Recommendations for PEAKachu, Piranha, and PureCLIP", stating: "To this day, a benchmarking dataset for CLIP-Seq data analysis does not exist because of missing experimental methods to verify predicted binding sites. It is therefore recommended to test more than one peakcaller and to test more than one parameter set, which is easily possible with CLIP-Explorer.", and again in section "Effects of Using Different Peakcallers for RBFOX2", where we say: "It is therefore possible to find a better ground truth, that is to say, a benchmark set with CLIP-Explorer, since more than one peakcaller can be tested with just a few clicks. A simple exchange with a different peakcaller is often not supported by other pipelines such as iCount."; and finally in the "Potential implications": "Based on our results we recommend to test more than one peak calling algorithm for other RBPs to assess the robustness of the motifs. CLIP-Explorer allows this very easily, because of its user-friendly interface. An exchange of the peakcaller can be done in an instant.".

We also now included more independent validations to stress out potential false positives in the peak sets of PEAKachu, Piranha, and PureCLIP and make clear that it is not easy to say which peakcaller performs "better". We stress this out in the discussion of the results of RBFOX2 in section "Effects of Using Different Peakcallers for RBFOX2" and in the discussion of the results for the RBP SLBP in section "Comparison of CLIP-Explorer's Results".
\end{answer}

For example,
$>>$"Several of the new motifs detected by CLIP-Explorer are also detected by CLIPper but not covered by the databases [43, 44] or any other literature to the best of our knowledge (Supplements: Table 1). In more detail, we find the new sequence motif CAGGCUGG for PTBP1, whereas LIN28B has the additional sequence motif CAGCCUG or the short motif CUCA. Finally, we find additional binding motifs for U2AF2 (ACAG) and QKI (AGGCU)."

Powered by Editorial Manager® and ProduXion Manager® from Aries Systems Corporation

The reliability of these motifs would be increased if the authors can present independent evidence (affected genes from knockdown RNA-seq, weaker enrichment in in vitro binding assays, etc) for at least one of these motifs to validate that these are not an unexplored source of false-positives. This is particularly true for cases where the RBP has a well-characterized binding modality (e.g., SLBP binds a highly conserved hairpin structure, and so the fact that CLIP-Explorer identifies significantly more peaks which enable identification of significant primary sequence motifs raises the question of whether these represent true binding motifs)

\begin{answer}
We checked the motif of PTBP1, as recommended, using independent data from ENCODE from a knockdown and control experiment. A new figure (Figure 3) shows the CDF of the log2 fold changes of the genes of hg38. The results are described as a validation in the section "Comparison of CLIP-Explorer’s Results" and the analysis is described in the method section "Analyzed Data". We could show that both PEAKachu and CLIPper peaks (binding sites) are probably true binding sites, as indicated by a significant shift of the CDF of the log2 fold changes in comparison to the non-targeted genes. The targets with the motif CAGGCUGG (from CLIP-Explorer) and with the motif CCAGGCUG (from CLIPper) show a significant shift as well, however not as prominent as the whole target ensemble of CLIP-Explorer and CLIPper. We also checked a different motif (UUCCUUUC), which was also identified by CLIP-Explorer and CLIPper. The motif seemed more significant then the motif CAGGCUGG. However, it also had more targets (715) than the motif CAGGCUGG (304) and CCAGGCUG (292). We can only speculate why the targets of the motif CAGGCUGG are not more influenced by the knockdown. We have not done the knockdown experiment and cannot make any statements about the quality or the results of the experiment. However, we can clearly state that all targets with the three motifs are significantly affected.
\end{answer}

Similarly:
$>>$For example, CLIPper finds 158 peaks and CLIP-Explorer (PEAKachu) 1052 for the protein SLBP. CLIP-Explorer predicts almost seven times more peaks than the CLIPper pipeline. We suspect that this difference is due to the computational model of CLIPper, which calls peaks for every replicate separately. Thus, we check the number of predicted peaks of the CLIPper pipeline for each replicate. The first replicate encompasses 9194 peaks, whereas the second replicate has 11686 peaks, which makes a total difference of 2492 peaks. Intersecting the peaks, without an irreducible discovery rate (IDR), of the two replicates with bedtools (see Methods) results in 1136 peaks.

The authors seem to imply in this section that the increased number of peaks correlates with quality; but as SLBP is well-characterized to exclusively bind to a hairpin structure within histone 3'UTRs (and there are only ~150 histone genes, which may include pseudogenes) it is unclear whether >1000 peaks is reasonable for SLBP. It would be helpful to include further analysis here to query whether these additional peaks represent true peaks that make sense biologically, or rather signal a potential source of false-positive signal. Are these additional peaks at (or near) histone 3'UTRs, or are they randomly distributed across other genes (or particularly at abundant noncoding RNAs or other locations that are common artifacts in many CLIP experiments)?

(It should be noted here that I believe the '9194 and 11686' numbers reflect CLIPper peaks without including a fold-enrichment or p-value cutoff for IP versus input, whereas the IDR peaks provided by the ENCODE project include these cutoffs as part of the IDR processing pipeline; so the larger numbers (clusters identified by CLIPper prior to input normalization) are not comparable to other peak callers that include input normalization.)

\begin{answer}
We did not intend to make any implications or quality assessment with this paragraph. It was just the description of the observed differences between peak sets of the two pipelines, that is to say, more importantly between the two peakcallers PEAKachu and CLIPper. Thus, we extended the analysis to find out what might cause the difference in peak numbers without making any quality assessments. Furthermore, we slightly

changed the wording from the previous paper to not mislead the reader into thinking that either PEAKachu or CLIPper "performs better".
\end{answer}

Along these lines, for the comparisons between peak callers, I would be more interested in deeper analysis of the distinct peaks identified rather than all peaks. For Figure 2, for example - I would be particularly interested to see the distribution in Fig 2b shown not for all peaks, but for the 7714/177/10589 peak caller-specific peaks shown in Fig 2a, as I think those would be most informative as to which peak caller is 'best'. Do the authors see fundamental differences in the peak-caller-specific peaks (i.e., does Piranha or PEAKachu tend to over-call peaks in repetitive elements in introns, to split broader peaks into many neighboring short peak calls, etc)?

\begin{answer}
We took the recommendation an adapted figure 2b. We investigated the different target distributions for the individual peakcallers (O; 7714/177/10589) and the peak set of the intersection with all peakcallers (I; 2134/2117/2769). We describe and discuss the results in section "Effects of Using Different Peakcallers for RBFOX2" of the paper, as we think this is an interesting question for the reader.

As stated now in the paper:
The target distribution of Piranha and PureCLIP changes for the individual peak set. The portion of intron coverage shrinks and the portion of exon targets increases as well as lincRNA, rRNA, snoRNA, and snRNA. In contrast, the target distributions of PEAKachu looks similar. However, the target distribution of the intersection of all three peakcaller looks different in comparison to the distribution of all peaks. PEAKachu's distribution has a smaller intron and a bigger exon and pseudogene portion. On the other hand, the peaks from the intersection set of Piranha and PureCLIP overlap more with introns and less with exons or any snRNA, snoRNA, tRNA, or rRNA.

We conclude from these observations:
a) The higher false positive rate in the Piranha peak set, since the peaks might cover false positives that lie in unspecific regions such as rRNAs.
b) False positives in the PureCLIP peak set, even though it might be more robust than Piranha, because of the change of the target distribution for the PureCLIP peaks that do not intersect with the peaks of the other two peakcallers. Like Piranha's distribution, peaks might overlap with unspecific regions such as rRNAs.
c) False positives in the PEAKachu peak set. As the paper states now: "PEAKachu's target distribution for peaks that do not intersect with the peaks of the other two peakcallers might also suggest some false positives, because of the bigger portion of peaks in pseudogenes in that peak set."

As also mentioned for reviewer 1, because of a missing ground truth, we validated the peakcaller now more on biological facts than technical features (e.g., false positives or accuracy). For that reason, we put a new Table and new results into the supplements of the paper (Supplementary Table 3 and 4).
\end{answer}

This is a particularly important question as the methods indicates the authors use a one-step mapping to the human genome, rather than the two-step approach the ENCODE pipeline used for eCLIP analysis (first removing repetitive elements by mapping against a database of RepBase and other elements, then followed by standard genomic mapping with STAR that requires unique mapping). I wonder if this is a major contributor to the large differences in number of identified peaks - are many of these peak caller-specific peaks at rRNA/tRNA/ncRNA pseudogenes throughout the genome?

\begin{answer}
We refer at this point to the aforementioned changes which also include a deeper analysis of the SLBP peaks based on rRNA/tRNA/ncRNA pseudogenes.
\end{answer}

Minor comments:

Powered by Editorial Manager® and ProduXion Manager® from Aries Systems Corporation

	Fig. 2b would be more informative with a different color scheme, as it's hard to identify quickly which color is which<br><br>\begin{answer}<br>We changed the color scheme and we hope it is more distinguishable now.<br>\end{answer}<br><br>$>>$However, CLIP-Explorer maps the reads to hg38, except for RBFOX2 (hg19), whereas the pipeline of the study by Nostrand et al. [13] used hg19. We therefore convert the peak coordinates of the CLIPper algorithm from hg19 to hg38 with CrossMap [45].<br>Although I think this is likely fine for the analysis described here, it should be noted that the ENCODE DCC has available pipeline output for both hg19 and hg38 genome versions (e.g. ENCFF390PJW for replicate 1 peak calls for RBFOX2 HepG2 in hg38)<br><br>\begin{answer}<br>For the sake of reproducibility and correctness, we used now directly the CLIPper peaks of hg38 from ENCODE, except for RBFOX2, where we used the hg19 peaks. We have changed the paragraph in section "CLIP-Explorer: A Versatile Pipeline for the Analysis of CLIP-seq Data" (removing the statement about CrossMap). The results have changed because of it. It had some meaningful impact because of a bug in CrossMap, thanks to your comment. That is to say, the motifs of CLIPper are different (see Supplements 1) and the peak intersections with PEAKachu and CLIPper (see Supplements 2).<br>\end{answer}<br><br>\section{Remark}<br><br>\begin{answer}<br>We highlighted the major changes in the paper red. Major changes involve the recommendation and questions of the reviewer. Other changed such as rearrangements of whole paragraphs and changes of figures are not highlighted and need to be checked again. However, they are mentioned in the answers of the reviewers.<br><br>Figures and Supplements that changed: Figure 2, Supplements 1 and 2.<br><br>We thank reviewer 1 and 2 for their ideas, recommendations and questions that improved the manuscript. We thank both reviewers for their time and wish them both a good day.<br>\end{answer}
**Additional Information:**	
**Question**	**Response**
Are you submitting this manuscript to a special series or article collection?	No
**Experimental design and statistics**<br><br>Full details of the experimental design and statistical methods used should be given in the Methods section, as detailed in our Minimum Standards Reporting Checklist. Information essential to interpreting the data presented should be made available in the figure legends.	Yes

Powered by Editorial Manager® and ProduXion Manager® from Aries Systems Corporation

Have you included all the information requested in your manuscript?	
**Resources**<br><br>A description of all resources used, including antibodies, cell lines, animals and software tools, with enough information to allow them to be uniquely identified, should be included in the Methods section. Authors are strongly encouraged to cite Research Resource Identifiers (RRIDs) for antibodies, model organisms and tools, where possible.<br><br>Have you included the information requested as detailed in our Minimum Standards Reporting Checklist?	Yes
**Availability of data and materials**<br><br>All datasets and code on which the conclusions of the paper rely must be either included in your submission or deposited in publicly available repositories (where available and ethically appropriate), referencing such data using a unique identifier in the references and in the "Availability of Data and Materials" section of your manuscript.<br><br>Have you have met the above requirement as detailed in our Minimum Standards Reporting Checklist?	Yes

Powered by Editorial Manager® and ProduXion Manager® from Aries Systems Corporation

Manuscript Click here to access/download;Manuscript;main.pdf

GigaScience, 2017, 1–11

doi: xx.xxxx/xxxx
Manuscript in Preparation
Technical Note

TECHNICAL NOTE

Galaxy CLIP-Explorer: a web server for CLIP-Seq data analysis

Florian Heyl[1,*], Daniel Maticzka[1], Michael Uhl[1] and Rolf Backofen[1,2,*]

[1]Bioinformatics Group, Department of Computer Science, University of Freiburg, Georges-Köhler-Allee 106, 79110 Freiburg, Germany and [2]Signalling Research Centres BIOSS and CIBSS, University of Freiburg, Schaenzlestr. 18, 79104 Freiburg, Germany

[*]heylf@informatik.uni-freiburg.de, backofen@informatik.uni-freiburg.de

Abstract

Background Post-transcriptional regulation via RNA-binding proteins (RBP) plays a fundamental role in every organism, but the regulatory mechanisms lack important understanding. Nevertheless, they can be fathomed by crosslinking immunoprecipitation in combination with high-throughput sequencing (CLIP-Seq). CLIP-Seq answers questions about the functional role of an RBP and its targets by determining binding sites on a nucleotide level and associated sequence and structural binding patterns. In recent years the amount of CLIP-Seq data skyrocketed, urging the need for an automatic data analysis that can deal with different experimental setups. However, noncanonical data, new protocols, and a huge variety of tools, especially for peak calling, made it difficult to define a standard.
Findings CLIP-Explorer is a flexible, and reproducible data analysis pipeline for iCLIP data that supports for the first time eCLIP, FLASH, and uvCLAP data. Individual steps like peak calling can be changed to adapt to different experimental settings. We validate CLIP-Explorer on eCLIP data, finding similar or nearly identical motifs for various proteins in comparison with other databases. In addition, we detect new sequence motifs for PTBP1, and U2AF2. Finally, we optimize the peak calling with three different peakcallers on RBFOX2 data, discuss the difficulty of the peak calling step and give advice for different experimental setups.
Conclusion CLIP-Explorer finally fills the demand for a flexible CLIP-Seq data analysis pipeline that is applicable to the up-to-date CLIP protocols. The paper further shows the limitations of current peak calling algorithms and the importance of a robust peak detection.

Key words: CLIP-Seq; Data Analysis; Galaxy; RNA; Protein

Findings

Background

RNA plays a fundamental role in many regulatory processes like splicing or translation. Yet, processes like translation also undergo regulatory steps involving proteins such as elongation factors. These RBPs (RNA-binding proteins) interact with their target RNA and form ribonucleoprotein complexes [1]. Studies have revealed the involvement of RBPs in stages like splicing, polyadenylation, localization, translation, stability, and degradation [2, 3, 4, 5]. So far more than a thousand RBPs have been identified in human cells [2, 6, 7]. Various RBPs have been linked to neurodegenerative diseases and various types of cancer [2, 4, 8, 9]. These observations emphasize the importance to explore the mechanisms behind the regulatory processes mediated by RBPs.

Crosslinking and immunoprecipitation (CLIP) facilitates the analysis of the interdependence between the proteome and transcriptome *in vivo* [10] by detecting binding sites for RBPs on a genome-wide level. Many CLIP protocols such as PAR-CLIP [11], iCLIP [12], or eCLIP [13] emerged over a short period of time and new methods are still in development [14]. All methods consist of three fundamental steps: crosslinking the

Compiled on: March 1, 2020.
Draft manuscript prepared by the author.

RBP of interest to its target RNAs, purification and immunoprecipitation of the resulting complexes, and high-throughput sequencing of the resulting RNAs. Despite these commonalities, protocols such as iCLIP or eCLIP perform additional steps to increase the precision of the CLIP-Seq experiment [13, 15, 16, 17], which have to be covered by additional analysis tasks. For example, iCLIP introduced random barcodes (unique molecular identifiers, short UMIs) to reduce the number of duplicated reads [12]. Protocols like eCLIP [13] and uvCLAP [18] adapted this procedure. A deduplication step is therefore imperative for iCLIP, eCLIP, FLASH, and uvCLAP [12, 19].

Because of the complexity and variety of CLIP protocols, the computational analysis is still the critical bottleneck, both in time and reproducibility. Individual tools that perform quality control, mapping, peak calling, and motif detection for CLIP-Seq data exist. However, an automatic and complete data analysis pipeline has to deal with a big list of obstacles such as biases that are introduced by the CLIP-Seq protocol and experimental conditions. On top of this, additional problems arise from changing hardware and tool versions, practicality of the user interface, different library formats (e.g., biological replicates or multiplexed data), and different CLIP-Seq data formats for old, recent, or upcoming protocols. Furthermore, each tool for each subtask has different assumptions and parameters that need to be optimized for the underlying protocol [19]. The most challenging task is the binding site identification, where a couple of different peakcallers, such as Piranha [1], PEAKachu [20], CLIPper [21], and PureCLIP [22], exist. For example, biological replicates are not supported by some peakcallers like Piranha [1]. These obstacles lead to a lack of reproducibility for the CLIP-Seq data analysis.

One possible solution could be one big, but fixed pipeline that can cope with every possible type of data. This solution was already tried in the case of PIPE-CLIP [23] or CLIPSeqTools [24]. Nevertheless, it is intractable to cover all possible combinations of different experimental settings, such as the number of replicates, the existence of a control library and others. Focussing instead on one specific type of data is easier to handle, like analyzing only iCLIP data with iCount [25]. However, neither PIPE-CLIP, nor CLIPSeqTools and iCount can be quickly and simply expanded or modified. They lack the option for an extension to cover noncanonical experimental data or new CLIP-Seq data types such as eCLIP, FLASH, or uvCLAP.

We hereby present CLIP-Explorer (https://clipseq.usegalaxy.eu/), a CLIP-Seq pipeline implemented in Galaxy [26]. CLIP-Explorer provides all necessary tools to analyze eCLIP, FLASH, uvCLAP and iCLIP data. CLIP-Explorer is well documented through an online tutorial in the Galaxy training material (https://galaxyproject.github.io/training-material/topics/transcriptomics/tutorials/clipseq/tutorial.html) and the main domain. Both websites assist the user to understand the main steps and parameters of the pipeline and the featured tools. The user can then, for example, replace the peakcaller or read mapper. It is not required to have detailed knowledge about the tools. CLIP-Explorer works in a server environment, thus the user does not have to worry about varying hardware or tool versions. A constant maintenance of CLIP-Explorer makes the data analysis easy to reproduce.

We have validated CLIP-Explorer on eCLIP data of DROSHA, HNRNPK, IGF2BP1, KHDRBS1, LIN28B, PTBP1, QKI, SLBP and U2AF2. We compared the results with a different analysis pipeline and databases, finding great diversity in the number of predicted peaks and found motifs. A more comprehensive analysis including the peakcallers Piranha, PureCLIP, and PEAKachu was done for RBFOX2 [13] as it has well documented targets and motifs. The protein RBFOX2 encoded by the gene RBM9 is a tissue-specific splicing factor involved in developmental processes [21, 27]. Studies have shown RBFOX2's binding preference for introns close to differentially spliced exons [28, 29]. The conserved sequence motif *TGCATG* has been shown to be enriched in RBPFOX2's binding sites [21, 28, 29]. Concerning the inconsistent results of the peak calling, we propose standard guidelines for the peak calling for different experimental setups. We confirm RBFOX2's binding characteristics from the literature as another validation for CLIP-Explorer.

CLIP-Explorer: A Versatile Pipeline for the Analysis of CLIP-Seq Data

Different experimental settings require different analysis pipelines, since pre-processing, mapping, peak calling and motif detection have to be adapted. For that reason, CLIP-Explorer integrates several pipelines for analyzing different protocols, namely eCLIP, iCLIP, FLASH and uvCLAP. Common to all pipelines in CLIP-Explorer is the division into four major steps (Figure 1). In the pre-processing, the read library is demultiplexed and, if necessary, adapter sequences as well as inline barcodes and UMIs are removed. In the post-processing, the reads are aligned and deduplicated. CLIP-Explorer then identifies differentially enriched regions (peaks) that are further analyzed according to genomic localization and other criteria to investigate the precise function of the protein and properties of its targets. All subtasks are accompanied by quality control steps. The versatility of CLIP-Explorer allows the user to select three different peak calling pipelines for three different data specifications. The methods section covers CLIP-Explorer in more detail. Additional information can be found in the Galaxy training material.

Recommendations for PEAKachu, Piranha, and PureCLIP

To this day, a benchmarking dataset for CLIP-Seq data analysis does not exist because of missing experimental methods to verify predicted binding sites. It is therefore recommended to test more than one peakcaller and to test more than one parameter set, which is easily possible with CLIP-Explorer.

The newest version of PEAKachu [20] is well-suited for an experimental setup with at least two replicates for the CLIP experiment and at least two replicates for the control experiment, because it uses DESeq2 [43]. It is therefore best to turn on the DESeq2 normalization. If the user has less replicates, the user can still use PEAKachu, but DESeq2 requires at least two replicates for both experiment and control to calculate p-values. With less than two replicates, PEAKachu filters the peaks based on the fold change and the mad (median absolute deviation) multiplier. It is therefore wise to check the peaks with another peakcaller or peak calling pipeline. PEAKachu works best in the adaptive mode with a mad multiplier of 0.0, a log2 fold change threshold of 2.0, and an adjusted p-value threshold of 0.05. PEAKachu needs the parameter of the maximum insert size, identified beforehand by Picard [44]. The estimation of the insert size is only necessary if the user provides paired-end read data. The window mode of PEAKachu is not recommended, because it is rather unstable. The mad multiplier might reduce the number of peaks, as it works as a second cutoff. To get the full peak set, it is wise to leave it at zero and filter the peaks by the log2 fold change together with the adjusted p-value. One key parameter is the minimum block overlap that has to be tested with the default of 0.5 in the beginning. The user has to increase this parameter if the results show a lot of peaks in a close vicinity. Another critical parameter is the minimum cluster expression fraction and the minimum block expression. These parameters can change the total number of

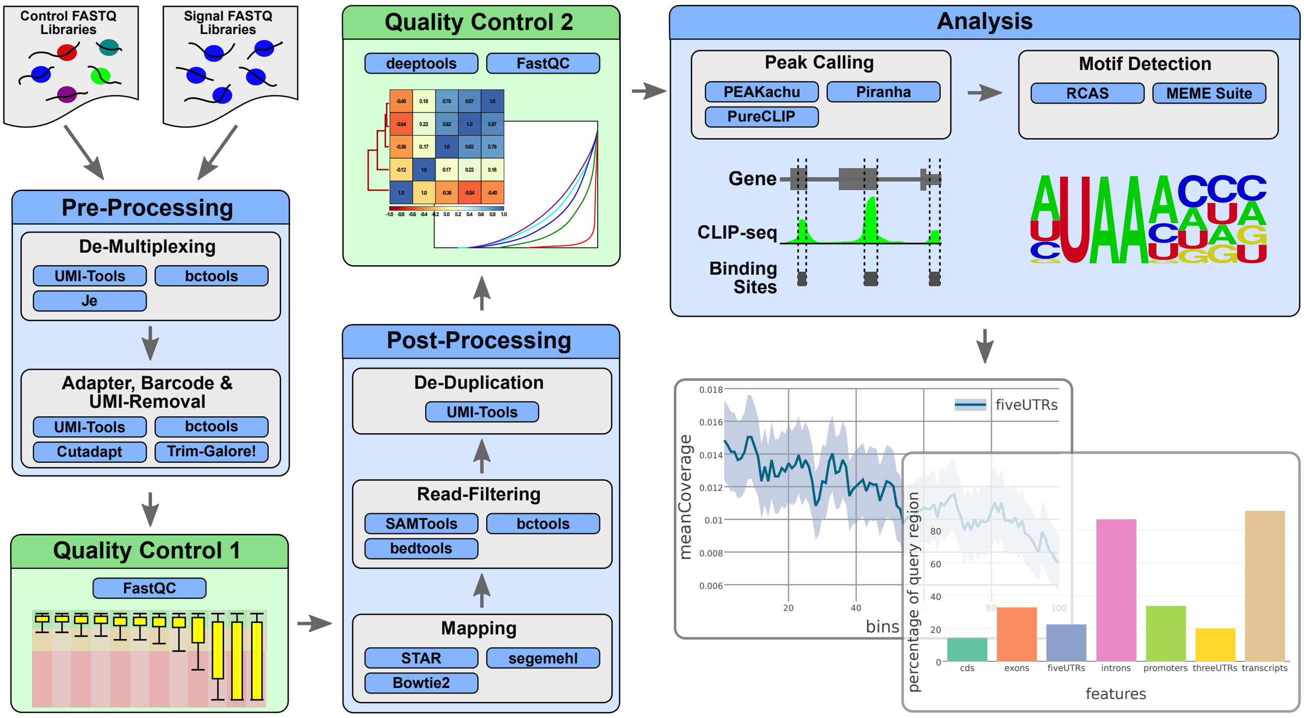

Figure 1. Flowchart of CLIP-Explorer; CLIP-Explorer has three major steps. In the pre-processing, the read library is demultiplexed, if necessary, and adapter sequences as well as in-line barcodes and UMIs are removed. CLIP-Explorer uses Je [30], UMI-Tools [31], bctools [32], and Cutadapt [33] for that purpose. A quality control step using FastQC [34] follows the pre-processing. In the post-processing, the reads are aligned with STAR [35], Bowtie [36] or segemehl [37], filtered using SAMtools [38], bedtools [39], and bctools, and deduplicated with UMI-tools. Another quality control, mainly with deeptools [40], checks the batch quality. Finally, CLIP-Explorer identifies differentially enriched regions using either PEAKachu [20], Piranha [1], or PureCLIP [22]. The binding regions are then analyzed with RCAS [41] and MEME-ChIP [42].

predicted peaks. Leave them in default with 0.01 and 0.1 and adjust the parameters if some interesting binding regions are not covered by PEAKachu's prediction. PEAKachu can be applied to iCLIP, eCLIP, FLASH, uvCLAP and even older protocols like PAR-CLIP.

Piranha [1] can be applied to an experimental setup with or without control, but it does not support replicates for the CLIP experiment. Each replicate has to be treated separately or further validated with a robust peak detection. However, if the user has only one replicate and no control, we recommend to use Piranha. If a control is provided, Piranha uses a zero-truncated negative binomial regression by default. Without a control it is wise to stick to a negative binomial. The distance to merge significant bins is one of the most crucial parameters of Piranha, similar to the minimum block overlap of PEAKachu. If the user observes a lot of peaks in a close vicinity, then this parameter has to be increased (e.g, 10). The bin size of the signal and control is another crucial parameter of Piranha that needs to be optimized. If the bin size is quite big (e.g., 200), then Piranha might miss a few good candidates. If the bin size is very small (e.g., 5), then Piranha predicts a lot of false positives. Piranha can also be applied to iCLIP, eCLIP, FLASH, uvCLAP, and even older protocols like PAR-CLIP.

PureCLIP [22] can be applied to an experimental setup with or without control, but it does not support replicates by the time of our analysis. It is therefore best to apply PureCLIP, as well as Piranha, to each replicate separately and find robust peaks by intersection, merging or calculating an IDR. Therefore, we recommend to use PureCLIP if the user has only one replicate for the CLIP and control experiment. PureCLIP already incorporates two default parameters sets. One set can be used if the protein is assumed to bind low complex motifs, which results in more broader and unspecific binding sites. PureCLIP predicts not only the binding region, but also the crosslinking sites. In our tests, PureCLIP quite often reported very small binding regions, almost identical to the crosslinking sites. It is therefore recommended to slightly extend the predicted binding sites (e.g, five to ten bases to the left and right) to cover the whole binding region. Furthermore, if the user provides paired-end reads, the mate containing the crosslinking event has to be provided explicitly. For iCLIP, FLASH, and uvCLAP this corresponds to the first mate, while for eCLIP it is the second mate. PureCLIP was specifically designed for eCLIP and iCLIP [22]. We recommend to use the peakcaller only for those protocols or other variants such as FLASH or uvCLAP.

It is not easy to find a standard peak calling algorithm with a standard parameter set. We tried to cover possible cases and recommendations for Piranha, PEAKachu, and PureCLIP, but these tools can change over time, or a new peakcaller might outrank them. The user can therefore find permanently updated recommendations and guidelines for a CLIP-Seq data analysis on CLIP-Explorer's main domain.

Effects of Using Different Peakcallers for RBFOX2

We use eCLIP data from DROSHA, HNRNPK, IGF2BP1, KHDRBS1, LIN28B, PTPB1, QKI, RBFOX2, SLBP, and U2AF2 from the study by Nostrand et al. [13] to validate CLIP-Explorer (see Methods). We first use the data of RBFOX2 to check the robustness and quality of the predicted binding sites of CLIP-Explorer, since the protein has a well known binding motif. We intersect the peaks of Piranha, PEAKachu, and PureCLIP with bedtools [39] (see Methods). Piranha detects the highest number of potential binding regions for RBFOX2 (Figure 2a). Yet, less more than one third of Piranha's peaks are not included in PEAKachu's and PureCLIP's peak set. PureCLIP has the highest fraction of peaks shared with the other two peakcallers, but it also has the lowest total number of peaks. PEAKachu on the other hand also has a high number of individual peaks, but significantly less

than Piranha. To check for the origin of the difference in the number of peaks, we compare all three peakcallers (PEAKachu, Piranha, PureCLIP), including the CLIPper peaks of the study by Nostrand et al. [13] for RBFOX2, regarding potential CLIP-Seq artifacts and biases. The table 1 shows that PEAKachu has the least amount of peaks overlapping with other PEAKachu peaks, whereas Piranha has only 59.83% that were non-overlapping with other Piranha peaks. In addition, almost 50% of the peaks from all peakcallers come from the plus or minus strand and the overlap with 3' or 5' UTRs is below 1%. PEAKachu has 362 peaks that overlapped with repeats (2.45%), however all peakcallers have a rate below 1% of peaks that overlap with intron repeats or any RNA pseudogene region. We also check the peak length and the distance between the peaks of the different peakcallers (Supplements: Table 3). PEAKachu has on average larger peaks with 131 nucleotides. On the other hand, PureCLIP

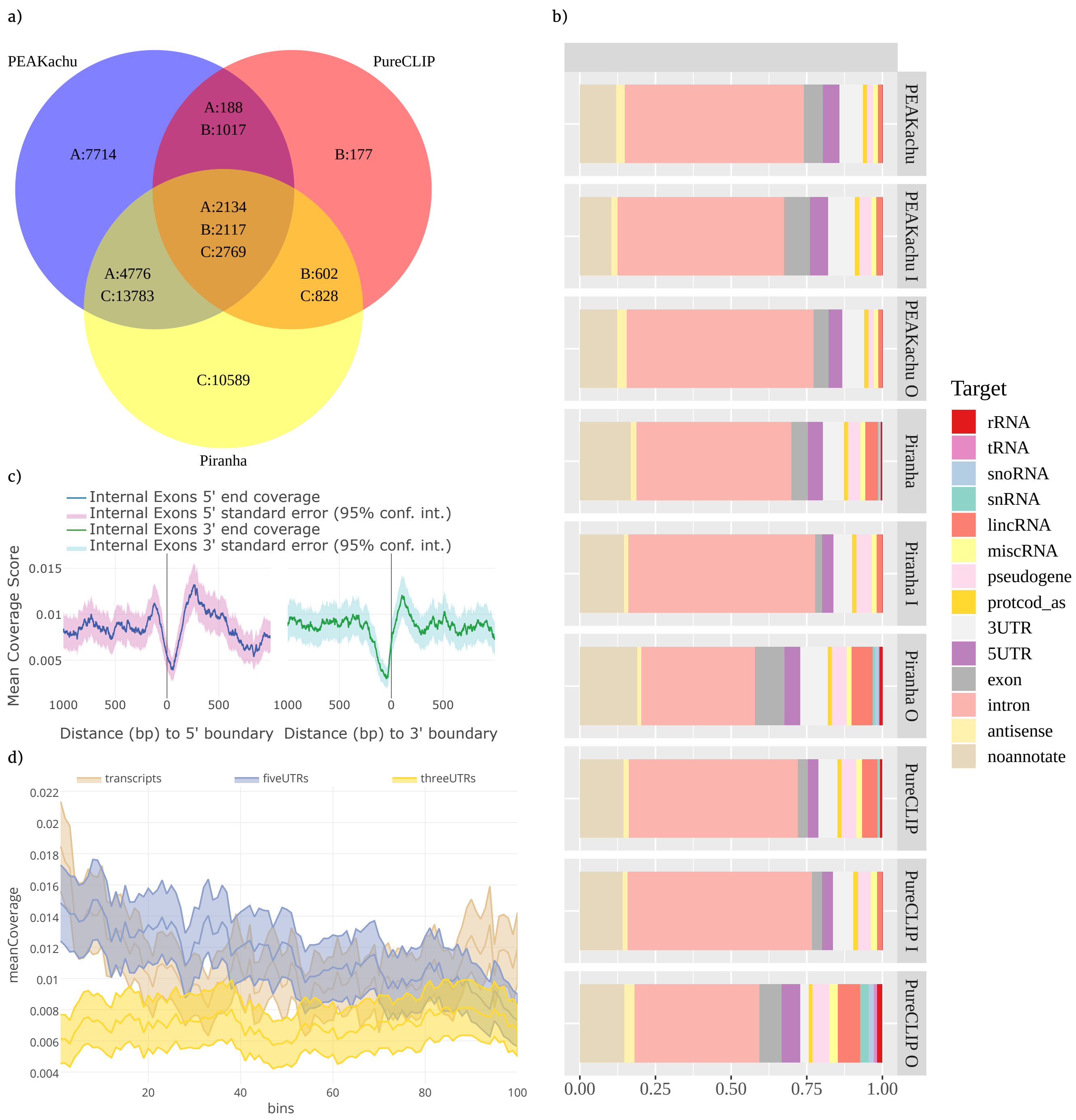

Figure 2. Comparison and analysis of the binding regions detected by PEAKachu, Piranha, and PureCLIP. The results are from the eCLIP data of RBFOX2 [13]. **(a)** We intersected the binding regions identified by the peakcallers with bedtools [39], paying attention to the strand (intersect -s), to assess the robustness of each method. **(b)** We then annotated the binding regions of each peakcaller and plotted the fraction of each target. We also analyzed separately the peak set of the individual peakcallers (O = peak set 7714/177/10589) and the peak set of the intersection with all peakcallers (I = peak set 2134/2117/2769). RBFOX2 prevalently binds introns, but also 3' and 5' UTRs as well as lincRNAs. The plot was generated with the hg19 script of targetdist [45]. Investigating the mean coverage of these binding regions identified by **(c)** PEAKachu [20] reveals an occupancy drop around the 5' and 3' ends of the exons. **(d)** Looking further at the mean coverage of the binding regions identified by PEAKachu in the overall transcript as well as for the 5' and 3' UTRs. The thickness of the ribbon around the mean coverage indicates the 95% confidence interval (mean +- standard error of the mean times 1.96). Each feature is divided into 100 bins of equal length, whereas features smaller than 100 bp are excluded [41].

Table 1. Percentage of peaks of the RBFOX2 peak set overlapping with the stated feature (P-genes = Pseudogenes).

Feature	PEAKachu	Piranha	PureCLIP	CLIPper
Own Peaks	0.36	40.17	21.73	17.56
Plus Strand	52.08	55.82	51.63	52.82
Minus Strand	47.92	44.18	48.37	47.18
3'UTR	0.42	0.02	0.03	0.09
5'UTR	0.96	0.05	0.03	0.27
Repeats	2.45	0.01	0.15	0.87
Intron Repeats	0.04	0.00	0.00	0.00
ncRNA P-genes	0.09	0.38	0.59	0.01
rRNA P-genes	0.00	0.07	0.05	0.00
tRNA P-genes	0.00	0.00	0.00	0.00

has the smallest peaks with an average of 36 nucleotides. The distribution of Piranha is a constant of 20 nucleotides because the peak length is a parameter the user defines for the tool. Furthermore, the distance between the peaks is almost identical between the peakcallers PEAKachu, PureCLIP and CLIPper (Supplements: Table 3). Only Piranha has slightly more peaks, which are close together.

To identify the type of bound genomic regions, we annotate the peaks discovered by the three different peakcallers. The distributions of binding sites generated by these three tools show a similar trend and prevalence for introns as the main target of RBFOX2 (Figure 2b). Yet, introns are not the only target. Our findings suggest that some binding regions of RBFOX2 lie in 3' and 5' UTRs, but this fraction is not as big as for introns. PEAKachu, Piranha, and PureCLIP further detect another chunk of target sites in lincRNAs, but the portion detected by PEAKachu is smaller, probably because of a low coverage of lincRNAs in CLIP protocols. In addition, we annotated separately the peak set of the individual peakcallers (7714/177/10589, from Figure 2a) and the peak set of the intersection with all peakcallers (2134/2117/2769). The target distribution of Piranha and PureCLIP changes for the individual peak set. The portion of intron coverage shrinks and the portion exon targets increases as well as lincRNA, rRNA, snoRNA, and snRNA. In contrast, the target distributions of PEAKachu looks similar. However, the target distribution of the intersection of all three peakcallers looks different in comparison to the distribution of all peaks. PEAKachu's distribution has a smaller intron and a bigger exon and pseudogene portion. On the other hand, the peaks from the intersection set of Piranha and PureCLIP overlap more with introns and less with exons or any snRNA, snoRNA, tRNA or rRNA.

To verify that RBFOX2 is a splicing factor, we investigate the peak profile of the binding sites by looking at the coverage plot of RBFOX2. PEAKachu depict a drop in the binding region coverage around the exon-intron-boundaries at the 5' and 3' ends (Figure 2c). The drop is more intense for the sites found by PEAKachu. Checking the mean coverage of the binding regions (Figure 2d), the results suggest a binding prevalence of RBFOX2 in the upstream region of the transcripts. Furthermore, RBFOX2 seems to target the beginning of the 5' UTR. In contrast, the binding coverage is homogeneous for the 3' UTR. The sequence *UGCAUG* seems to play an important part for the binding of RBFOX2 as it is among the top five motifs detected by the PEAKachu, Piranha and PureCLIP pipeline (see Table 2). The second motif shows guanine richness and the third motif cytosine and uracil richness for all peakcallers.

At the end, we check the function of RBFOX2 to clarify the role in human liver cancer cells (Hep G2). Looking at the top one hundred genomic regions that have RBFOX2 binding sites, we find Shank2 and Shank3 among the top hits as potential targets. Furthermore, a gene ontology (GO) analysis with RCAS for the targets of RBFOX2 identifies the protein to be relevant for the regulation of RNA splicing (with a Benjamini-Hochberg (BH) adjusted p-value of $< 10^{-4}$), as well as regulation of transcription by RNA polymerase I (BH adjusted p-value $< 10^{-3}$). In addition, the GO analysis identifies RBFOX2 to be involved in the regulation of histone modifications (BH adjusted p-value $< 10^{-4}$), nucleosome and nucleosomal binding (BH adjusted p-value $< 10^{-4}$ and 0.04, respectively) and methyl-CpG binding (BH adjusted p-value < 0.04).

Checking the results for RBFOX2, Piranha found the highest number of peaks. A lot of these peaks, however, might represent false positives since Piranha was executed without the information from the control experiments (see Methods). More than one third of Piranha's peaks were not included in PEAKachu's and PureCLIP's peak set, which endorses the supposition. The observation is also substantiated by the change of the target distribution of the Piranha peaks that do not intersect with the peaks of the other two peakcallers. These peaks might cover false positives that lie in unspecific regions such as rRNAs. In addition almost 40% of Piranha's peaks were overlapping with other Piranha peaks, that is to say, from a total amount of 27,969 peaks the set had 11,236 peaks that cover similar regions. Furthermore, the distance between the peaks is almost identical between the peakcallers. Only Piranha has slightly more peaks, which are closer together indicating a higher number of false positives because it calls many peaks (local maxima) in close proximity and does not combine them into a bigger peak (global maximum). In contrast, PureCLIP had the lowest number of predicted peaks and the highest fractions of peaks shared with the other peakcallers. This indicates that PureCLIP selects the peaks based on very stringent criteria. However, PureCLIP might therefore also have a high false negative rate. PureCLIP calls peaks for each replicate separately, which is likely the reason for missing some good candidates that have been jointly found by PEAKachu. Furthermore, the change of the target distribution for the PureCLIP peaks that do not intersect with the peaks of the other two peakcallers might show possible false positives. Like Piranha's distribution, peaks might overlap with unspecific regions such as rRNAs. PEAKachu, on the other hand, had the highest fraction of peaks that do not overlap with other PEAKachu peaks, because of a larger peak length. We cannot make a general judgement about a good value for the peak length, because each RBP and each binding region can be specific and a benchmarking dataset is missing for CLIP-Seq data. PEAKachu's target distribution for peaks that do not intersect with the peaks of the other two peakcallers might suggest some false positives, because of the bigger portion of peaks in pseudogenes in that peak set. We also checked for potential CLIP-Seq biases to answer the question of the different number in peaks, but we could not find any evidence regarding a significant strand, UTR or repeat bias for any of the implemented peakcallers.

Despite the disparate numbers of peaks between PEAKachu, Piranha, and PureCLIP, the main motif of RBFOX2 with the sequence *UGCAUG* was identified with CLIP-Explorer for all used peakcallers. The motif seems to be very robust to varying peak calling conditions, which is endorsed by the fact that it can be even found with PureCLIP with a different pre-processing (see Table 2). We tested the peakcallers PEAKachu, Piranha, and PureCLIP on the alignments files from the study by Nostrand et al. [13]. With CLIP-Explorer all three peakcallers found the main motif with lower background noise as in comparison to the already processed alignments files from ENCODE. Furthermore the motif set from CLIP-Explorer looks more similar between all three peakcallers (see Table 2). It is therefore possible to find a better ground truth, that is to say, a benchmark set with CLIP-Explorer, since additionally more than one peakcaller can be tested with just a few clicks.

Table 2. Top five RBFOX2 sequence motifs for each peakcaller identified by MEME-ChIP with E-value and the fraction of sequences with that specific motif, based on the RBFOX2 eCLIP data.

Pre-processing CLIP-Explorer			Pre-processing Nostrand et al. [13] (ENCODE)		
PEAKachu with 14,812 peaks	Piranha with 27,969 peaks	PureCLIP with 3,913 peaks	PEAKachu with 19,254 peaks	Piranha with 214,506 peaks	PureCLIP with 38,734 peaks
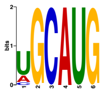 6.1e-702; 37.21%	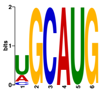 6.6e-381; 16.43%	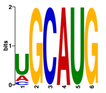 7.7e-288; 40.20%	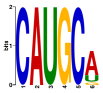 1.3e-1052; 39.61%	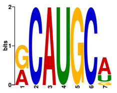 2.1e-983; 5.37%	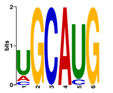 4.7e-2137; 34.07%
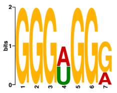 3.2e-075; 30.91%	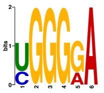 1.4e-065; 16.50%	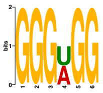 4.1e-030; 27.17%	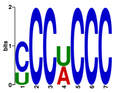 2.2e-118; 30.00%	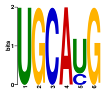 5.9e-366; 3.90%	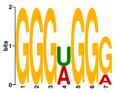 2.9e-215; 13.51%
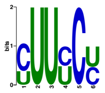 2.7e-049; 22.19%	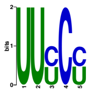 3.2e-057; 18.30%	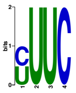 8.2e-009; 18.43%	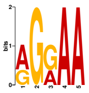 1.7e-083; 34.65%	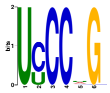 2.2e-329; 13.75%	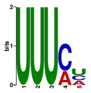 1.4e-107; 16.41%
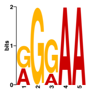 6.2e-045; 33.57%	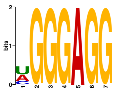 1.4e-038; 10.32%	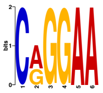 7.6e-006; 2.79%	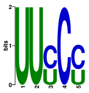 1.3e-058; 33.98%	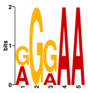 6.9e-298; 14.15%	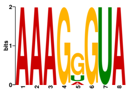 2.1e-052; 0.67%
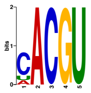 1.3e-027; 7.44%	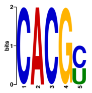 2.2e-031; 7.57%	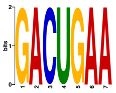 3.0e-005; 0.97%	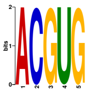 2.5e-046; 5.50%	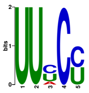 4.2e-146; 11.52%	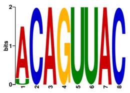 4.7e-049; 0.56%

We further tried to verify RBFOX2's known role as splicing factor and that it preferably binds to introns [21, 27, 28, 29] to substantiate the credibility and versatility of CLIP-Explorer. The distributions of binding sites generated by PEAKachu, Piranha, and PureCLIP showed a prevalence for introns as the main target of RBFOX2 in accordance with the literature [28, 29], which is further supported by the target distribution of the intersected peaks for all three peakcallers. The drop of the binding occupancy of RBFOX2 around the exon-intron boundaries can also be seen in other studies [28, 29]. The binding coverage of RBFOX2 around the exon-intron-boundaries suggests an involvement of RBFOX2 in the regulation of splicing. The GO term analysis corroborates the hypothesis linking RBFOX2 to various splicing and structure-related processes, such as histone modifications. Besides, the Shank gene family members, as a potential target of RBFOX2, play an important part in neuronal functions, where alterations in the encoded proteins may be connected to autism [21]. Shank2 and Shank3 might hereby be regulated by alternative splicing [46, 47]. Another study has found the same interdependence of RBFOX2 and the Shank protein family [21].

Comparison of CLIP-Explorer's Results

We compare the sequence motifs detected by CLIP-Explorer with two different databases [48, 49], and then with the peaks identified in the study by Nostrand et al. [13] (Supplements: Table 1). Here, the CLIPper algorithm [21] was used to identify potential binding regions of the same proteins. For the CLIPper peaks, we predict the sequence motifs with MEME-ChIP in the same way as implemented in CLIP-Explorer.

As a first step, we focus on the sequence motifs that resulted from CLIP-Explorer using PEAKachu for peak calling. The motifs are similar and sometimes nearly identical to the motifs listed in the databases for HNRNPK, KHDRBS1, PTBP1, QKI, RBFOX2, and U2AF2 (Supplements: Table 1). For example, the QKI-motif *ACUAA* [50] or the known motif *UGCAUG* of RBFOX2 [21, 28, 29] can be found in the databases and is also detected by CLIP-Explorer and CLIPper. Some proteins such as DROSHA, LIN28B, and SLBP are not listed in the databases, and the proteins IGF2BP1 and RBFOX2 have only one or two motifs. CLIP-Explorer identifies new motifs for these proteins. Several of the new motifs detected by CLIP-Explorer are also detected by CLIPper but not covered by the databases [48, 49] or any other literature to the best of our knowledge (Supplements: Table 1). For example, we find the new sequence motif *CAGGCUGG* for PTBP1, or the motif *ACAG* for U2AF2.

For some proteins, such as DROSHA and HNRNPK, the motifs detected by CLIP-Explorer deviate from the corresponding CLIPper motifs, but still show similar sequence compositions (Supplements: Table 1). In addition, CLIPper slightly misses the known motif *GGAGA* of LIN28B [51].

For a more fine-grained comparison between the PEAKachu pipeline in CLIP-Explorer and the CLIPper pipeline in Nostrand et al. [13], we intersect the peaks for each protein with bedtools [39] to check for common binding sites. We use a strict overlap for bedtools of at least one base (see Methods). This comparison reveals a discrepancy between some proteins. Less then ten percent of all PEAKachu peaks overlap with the CLIPper peaks of the proteins KHDRBS1 and SLBP (Supplements: Table 2). Quite often, PEAKachu finds more peaks in comparison to CLIPper, except for QKI and U2AF2. For example, CLIPper finds 161 peaks and CLIP-Explorer (PEAKachu) 1052 for the protein SLBP. Using PEAKachu, CLIP-Explorer predicts almost seven times more peaks than the CLIPper pipeline. We suspect that this difference is due to the computational model of CLIPper, which calls peaks for every replicate separately. Thus, we check the number of predicted peaks of the CLIPper pipeline for each replicate. The first replicate encompasses 9194 peaks, whereas the second replicate has 11686 peaks, which makes a

total difference of 2492 peaks. Intersecting the peaks without an irreducible discovery rate (IDR) of the two replicates (see Methods) results in 1136 peaks, so almost the same number as PEAKachu. We also check for potential CLIP-Seq biases (Supplements: Table 4). As a result, PEAKachu has 90.3% peaks that do not overlap with other PEAKachu peaks. CLIPper has 58% of peaks that do not overlap with other CLIPper peaks. Both PEAKachu and CLIPper have almost 60% of peaks from the plus strand and less than 1% of peaks from 3' or 5' UTRs. However, PEAKachu has some peaks (~ 16.25%) in repeats, which do not overlap with intron repeats, and it has some peaks in ncRNA pseudogenes (~ 0.022%) and one peak that overlaps with an rRNA pseudogene. PEAKachu also finds more peaks that overlap with ncRNA (321 peaks) than CLIPper (17 peaks). We also check the number of peaks overlapping with histone genes and their UTRs since SLBP targets mainly histone RNAs. Both PEAKachu and CLIPper find almost the same amount of peaks in histone regions (137 and 135 peaks, respectively), whereas PEAKachu finds more peaks in histone UTRs (35 and 6 peaks, respectively).

Our results revealed that different peakcallers deliver greatly varying sets of binding sites. We analyzed the discrepancy between the pipeline by Nostrand et al. [13] with the CLIPper algorithm [21] and CLIP-Explorer in more detail, using detected motifs and preferred genomic binding locations as quality criteria. Our analysis indicated that the differences between CLIPper and PEAKachu (CLIP-Explorer) might result from the fact that CLIPper was called for each CLIP replicate separately, followed by a robust peak detection between the two peak sets. PEAKachu, on the other hand, was called with all replicates in mind. Consequently, the difference in the peak set of PEAKachu (CLIP-Explorer) might come from a different noise estimation between replicates, thus detecting other binding sites. The results for SLBP endorse the conjecture, which showed a big difference in the number of peaks between CLIPper and CLIP-Explorer with PEAKachu. We found that roughly only 10% of the CLIPper peaks were contained in both replicates. This variation between replicates is in agreement with the literature [52]. We checked for potential CLIP-Seq biases and found no difference between PEAKachu and CLIPper for a specific strand and 3' or 5' UTR. Furthermore, both PEAKachu and CLIPper almost found the same amount of peaks in histone regions, despite PEAKachu finding slightly more peaks that overlapped with histone UTRs. However, PEAKachu also found more peaks in repeat regions and ncRNAs, which explains the difference in the total number of peaks and might represent false positives. The eCLIP pipeline from Nostrand et al. [13] already removed repeat regions in a double mapping approach.

To check the correctness of the identified new motifs such as *CAGGCUGG* for PTBP1, we took independent RNA-Seq data from ENCODE from a knockdown and control experiment for PTBP1 (see Methods). We intersected the identified binding sites of PTBP1 from CLIP-Explorer (PEAKachu) and CLIPper with the genes of hg38 and calculated the log2 fold change of all genes. Figure 3 clearly shows a significant shift in the cumulative density function (CDF) of the fold changes. This observation was expected since true binding sites should have different RNA rates in a knockdown experiment. Thus, the identified sites from PEAKachu and CLIPper might be real binding sites of PTBP1 as both peakcallers show similar CDFs even though the processing of the data was different. Thus we checked the motif *CAGGCUGG* (from CLIP-Explorer), *CCAGGCUG* (from CLIPper), and another motif *UUCCUUUC* (from CLIP-Explorer and CLIPper). All three CDFs are significantly shifted. Consequently, *CAGGCUGG* might not be a false positive.

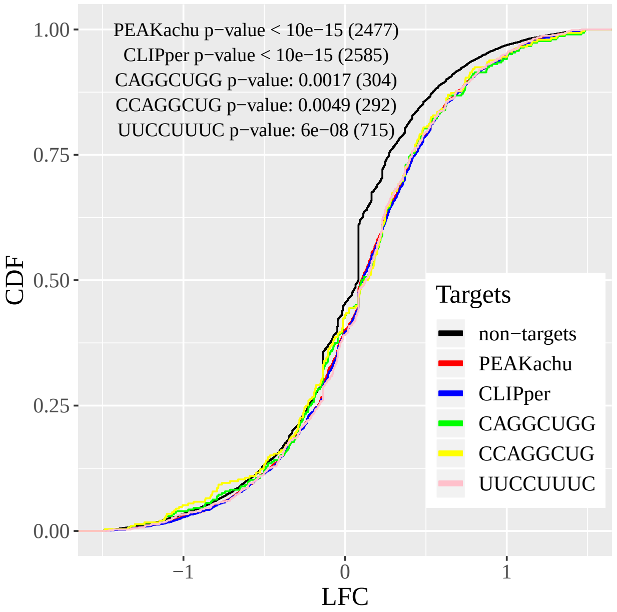

Figure 3. Cumulative density function (CDF) of the log2 fold changes of the genes of hg38 for a knockdown experiment of PTBP1; The identified targets of CLIPper and PEAKachu have nearly identical trends and show a significantly shifted CDF (p-values of one-sided t-test in the plot), as well as the targets with the identified motifs *CAGGCUGG* (CLIP-Explorer), *CCAGGCUG* (CLIPper), and *UUCCUUUC* (CLIP-Explorer and CLIPper) with p-values of one-sided Wilcoxon test, in comparison to the non-targets. The number of targets are listed after the p-value.

Potential Implications

CLIP-Explorer is a valuable tool for researchers working with CLIP-Seq data as it simplifies and integrates many processing steps in a well-tested and optimized pipeline. CLIP-Explorer provides the user with an extensive overview of the potential function of the RBP and its target RNAs. It can be easily extended or modified, and has no installation overhead as it is integrated in Galaxy. CLIP-Explorer is thus the first general and fully automatic pipeline for eCLIP, FLASH, and uvCLAP data, which can also be applied to iCLIP and other types of CLIP-Seq protocols. Besides, it is permanently maintained, and new tools can be implemented or exchanged with existing ones to warrant a highly efficient data analysis.

We analyzed different eCLIP datasets and compared our findings with different databases [48, 49] and the results from the study by Nostrand et al. [13]. The analysis of different proteins, such as DROSHA, HNRNPK, and in more detail RBFOX2, showed the strength of the flexibility provided by CLIP-Explorer. We could identify similar and even new sequence motifs for PTBP1 with the motif *CAGGCUGG*, and U2AF2 with *ACAG*. We also verified the sequence motif *UGCAUG* for RBFOX2 and found other guanine, cytosine and uracil-rich binding regions. The most significant sequence motif *UGCAUG* of RBFOX2 was found by Piranha, PEAKachu, and PureCLIP, even though the three peakcallers predicted three substantially different peak sets. Based on our results we recommend to test more than one peak calling algorithm for other RBPs to assess the robustness of the motifs. We showed that different peak calling tools might result in different peak sets and thus encompass different false positives and negatives. Yet, CLIP-Explorer allows this very easily, because of its user-friendly interface. An exchange of the peakcaller can be done in an instant. CLIP-Explorer is also essential for a later data investigation such as sequence motif detection or GO term analysis, since it reduces noise in the data. CLIP-Explorer enabled us to identify the in-

volvement of RBFOX2 in splicing because of the availability of specific annotation tools like RCAS [41]. We could identify a decrease of RBFOX2 occupancy around the exon-intron boundaries, linked RBFOX2 to the regulation of splicing and DNA structural modifications and verified other observation from previous studies.

We recently have integrated GraphProt [53] into our pipeline, which is one of the popular tools for RBP binding profile predictions. We will also include StoatyDive [54] in a later version for a newly developed peak shape clustering for a better noise reduction, as it showed promising results for CLIP-Seq data. All in all, CLIP-Explorer is a flexible and easily extendable pipeline, which greatly simplifies CLIP-Seq data analysis on a transcriptome- and genome-wide scale.

Methods

CLIP-Explorer includes all major processes that are required to analyze CLIP-Seq data. All tools for each step were selected based on review [55, 19, 17] or benchmark articles (stated for each tool later in the paper), or experiences. The analysis involves three major steps as shown in Figure 1, each followed by a specific quality control. In the pre-processing step, the data is demultiplexed into the read libraries stemming from different experiments. If necessary, adapter sequences (using Cutadapt [33, 56]) as well as in-line barcodes and UMIs are removed. FastQC [34] performs during that step a standard quality check for the read and library quality.

The post-processing for CLIP experiments is similar to RNA-seq experiments, that is, the reads are aligned and filtered. An additional deduplication step is required in the case of recent CLIP-Seq protocols such as iCLIP and eCLIP. The deduplication removes PCR duplication artifacts. CLIP-Explorer performs another quality control during the post-processing using mainly FastQC [34], and deeptools [40].

The final and major part of CLIP-Explorer is the analysis step. CLIP-Explorer searches in that step for sequence and coverage motifs, and potential targets of the investigated RBP. Peak calling and motif detection are the fundamental and most critical processes. The quality and amount of detected binding sites can vary significantly based on the used tools, whereas the tools depend heavily on the experimental setup. Different tools therefore lead in part to different results. For that reason, CLIP-Explorer provides several peak calling and motif detection tools to fathom the robustness of the results.

Input and Output of CLIP-Explorer

The user needs to provide their experimental data in FASTA or FASTQ format. Nonstandard adapter sequences can be provided by the user. Otherwise, CLIP-Explorer automatically detects them. The pipeline is designed for multiplexed or demultiplexed paired read data and supports replicates and control experiments. Barcode sequences are required in case of demultiplexing. Other additional files are provided by the Galaxy database. CLIP-Explorer can be easily changed, for example, to allow for single end reads and different tool settings.

The user obtains a MultiQC [57] report for the raw, trimming, alignment, and deduplication quality to assess the quality of the raw data and important processing steps of CLIP-Explorer. MultiQC collects the FastQC reports made during the pre- and post-processing of CLIP-Explorer to inspect the mapping quality, elaborating on the amount of unmapped and multiply mapped reads, the length of mapped reads, and other important characteristics of the read library. Further quality control is provided by deeptools, as it can elicit differences in signal and control experiments. A heatmap and a fingerprint plot assess the correlation between the signal and control libraries providing evidence for a correct execution of the CLIP experiment. CLIP-Explorer also generates coverage files (bigWig and bedGraph) for the alignments and the crosslinking sites to inspect the peak calling quality. Most importantly, CLIP-Explorer will produce a bed and gtf file of significantly enriched regions, representing the binding sites of the protein on the transcriptome or genome. A MEME-ChIP [42] report will further analyze the peaks, detecting potential sequence motifs of the protein. A FIMO [42] report then lists reference sequences, which where not covered by the peak calling, but contain the detected sequence motifs. Finally, a RCAS (RNA centric annotation system) [41] report determines the target distribution of the protein over RNA classes and transcript regions. It also includes a GO term analysis and plots to fathom the coverage of the protein binding around splice junctions, along the transcripts and along various other regions. CLIP-Explorer can also generate a list of robust peaks (shared between all input files). This feature is useful for peakcallers that do not support replicated data such as Piranha.

Mapping and Deduplication

We integrated STAR [35] into CLIP-Explorer to map reads against the genome based on the good performance and usability of STAR for RNA-Seq data [58, 59, 60]. STAR is an annotation and splice aware aligner, which is important for transcriptomic data. Thus, we used extra information about the transcriptome. The data of RBFOX2 was mapped against hg19 to better reproduce the literature results, all other proteins such as DROSHA and HNRNPK were mapped against hg38. STAR was executed with the *two pass mode* turned on and in the end-to-end alignment scheme. CLIP-Explorer further checks for incomplete pairs, ambiguously mapped and low quality reads. CLIP-Explorer also includes a deduplication step to lower the false positive rate for the identification of binding regions. PCR duplicates are often collapsed into one representative [2, 19]. CLIP-Explorer identifies potential PCR duplicates with the help of UMI-tools [31]. Duplicated reads are identified after the alignment step, searching for reads with identical genomic positions (begin and end) and orientation. Yet, sequencing errors can also occur in the UMIs. UMI-tools clusters the reads based on their UMI to handle these sequencing errors. Thus, we merged sequences with a high node count and a small Hamming distance between unique UMIs [31].

Identification of Enriched Regions and Sequence Motifs

Searching for enriched regions and motifs is the most challenging task because of the differences in the gene expression between CLIP experiments and background controls. Hence, high false negative rates are a common result in the detection of differentially enriched regions (peaks) [19]. CLIP-Explorer allows to choose between three different peakcallers, namely PEAKachu [20], PureCLIP [22], and Piranha [1]. PEAKachu was executed in adaptive mode with a mad multiplier of 0.0, a log2 fold change threshold of 2.0, an adjusted p-value (Benjamini-Hochberg procedure) threshold of 0.05 and a maximum insert size of 200, identified beforehand by Picard. All other parameters are set to their default values. We used for Piranha a negative binomial distribution with a bin size of 20 and a 0.05 p-value threshold. We did not include the control data for Piranha to test for possible experiments without control datasets. PureCLIP was trained on chromosome one, two, and three of hg38 (for RBFOX2 we used hg19, respectively), and executed

with *-bc o* as the default option. The resulting binding regions of PEAKachu, Piranha, and PureCLIP were then extended by 20 nucleotides because many peakcallers often call peaks that stop before the motif itself. The resulting regions were analyzed with RCAS [41] to determine the target distribution over genomic regions and possible binding patterns of RBFOX2. The peaks were also analyzed with the MEME Suite [42] tool package (MEME-ChIP) to find sequence motifs in the peaks. We used MEME-ChIP because of its versatility and performance for known motifs [61, 62]. Because of a missing ground truth for CLIP-Seq data, a benchmarking for motif finding tools is still missing. MEME-ChIP was set to find zero or one occurrence of the motif sites per sequence (zoops model).

Intersecting Peaks

We used the intersect module of bedtools [39] to assess the occurrence of the predicted peaks between CLIPper and CLIP-Explorer with PEAKachu, and between the three different CLIP-Explorer pipelines with Piranha, PureCLIP, and PEAKachu. We set bedtools intersect with the option *-s* to search for intersections on the same strand and kept the default value for *-f*, resulting in a minimum overlap of one base for overlapping regions to be reported. Further, we used the flag *-u* to consider only unique overlaps.

Analyzed Data

We used eCLIP data from DROSHA (ENCSR653HQC), HNRNPK (ENCSR828ZID), IGF2BP1 (ENCSR744GEU), KHDRBS1 (ENCSR628IDK), LIN28B (ENCSR861GYE), PTBP1 (ENCSR981WKN), QKI (ENCSR570WLM), RBFOX2 (ENCSR987FTF), SLBP (ENCSR483NOP), and U2AF2 (ENCSR202BFN) from the study by Nostrand et al. [13] to validate CLIP-Explorer. The data, which originated from human liver cancer cells (Hep G2) and immortalized myelogenous leukemia cells (K562), comprised two CLIP-Seq replicates and one control library for each RBP.

RBFOX2 was analyzed by CLIP-Explorer with PEAKachu, PureCLIP, and Piranha based on hg19. We took the peaks from the CLIPper pipeline (hg19 peaks ENCFF154DRN) and analyzed them with MEME-ChIP.

All other proteins were analyzed by CLIP-Explorer with PEAKachu on hg38. For the CLIPper pipeline we used the peaks from the IDR (signal normalization) from hg38 and analyzed them with MEME-ChIP.

We also analyzed data from an shRNA knockdown experiment against PTBP1 in Hep G2 cells followed by RNA-seq (ENCSR064DXG), including a control shRNA against no target (ENCSR603TCV). Both data samples are from ENCODE and encompass two replicates for both experiments. We took the alignments that were mapped with STAR against the genome (hg38). We calculated the coverage for each gene in hg38 with htseq-count [63]. We then obtained the log2 fold change for each gene from DESeq2 [43], taking both the knockdown and the control experiment into account. We then intersected the identified sites from PEAKachu and CLIPper for PTBP1 with the genes of hg38 and plotted the CDF of the log2 fold changes.

Availability of Supporting Source Code and Requirements

Project name: CLIP-Explorer
Project home page: https://clipseq.usegalaxy.eu/
Operating system(s): Galaxy
Training material: https://galaxyproject.github.io/training-material/topics/transcriptomics/tutorials/clipseq/tutorial.html
biotools:CLIP-Explorer
SciCrunch.org: SCR_018128

Availability of Supporting Data and Materials

CLIP-Explorer provides a small dataset for a test run, which can be found in the training material and on the CLIP-Explorer website. The whole eCLIP data used in this paper, such as RBFOX2 or PTBP1, is listed in the supplementary of the study by Nostrand et al. [13].

Additional files

Supplementary Table 1. Top five DREME sequence motifs of MEME-ChIP [42] of Different Proteins. CLIP-Explorer's sequence logos of different proteins from the binding regions that were identified by PEAKachu. Furthermore, sequence motifs of MEME-ChIP from the binding regions that were identified by the CLIPper algorithm [21]. The sequence motifs of CLIP-Explorer and CLIPper originated from eCLIP data [13]. To compare the sequence logos other motifs were collected from different databases [48, 49].

Supplementary Table 2. Peak intersections between PEAKachu [20] of CLIP-Explorer and CLIPper from the the study by Nostrand et al. [13]. A list of Venn diagrams showing the overlap between PEAKachu [20] (CLIP-Explorer) and CLIPper peaks [13].

Supplementary Table 3. Comparison of the peak length and the distance between the peaks between PEAKachu, Piranha, PureCLIP, and CLIPper for the RBFOX2 data.

Supplementary Table 4. Number of peaks of the SLBP peak set of PEAKachu and CLIPper overlapping with the stated features (P-genes = Pseudogenes).

Declarations

List of abbreviations

CLIP-Seq: Crosslinking immunoprecipitation in combination with high-throughput sequencing; CDF: Cumulative density function; IDF: irreducible discovery rate; RBP: RNA-binding proteins.

Ethical Approval

Not applicable

Consent for Publication

Not applicable

Competing Interests

The authors declare that they have no competing interests.

Funding

This study was funded by the Deutsche Forschungsgemeinschaft (DFG, German Research Foundation) grant

322977937/GRK2344 2017 MeInBio – BioInMe Research Training Group, Germany's Excellence Strategy (CIBSS – EXC-2189 – Project ID 390939984), DFG grant BA2168/11-2 SPP 1738, DFG grant TRR 167/1 2027 NeuroMac, and by the Collaborative Research Centre 992 Medical Epigenetics (DFG grant SFB 992/2 2016).

Author's Contributions

F.H. and D.M. performed the computational analysis. R.B., and D.M. initialized the project, and supervised the research. F.H. and R.B. wrote the manuscript with inputs from other authors. M.U. integrated Graphprot, PureCLIP and RCAS into CLIP-Explorer. All authors read and approved the final manuscript.

Acknowledgements

We are grateful to the members of the Galaxy team Freiburg (http://www.bioinf.uni-freiburg.de/Galaxy/) and to Torsten Houwaart for his support.

References

1. Uren PJ, Bahrami-Samani E, Burns SC, Qiao M, Karginov FV, Hodges E, et al. Site identification in high-throughput RNA-protein interaction data. Bioinformatics 2012;28(23):3013–3020.
2. Chakrabarti AM, Haberman N, Praznik A, Luscombe NM, Ule J. Data Science Issues in Understanding Protein-RNA Interactions. bioRxiv 2017;.
3. Hentze MW, Castello A, Schwarzl T, Preiss T. A brave new world of RNA-binding proteins. Nature Reviews Molecular Cell Biology 2018;19:327–341.
4. Baltz AG, Munschauer M, Schwanhäusser B, Vasile A, Murakawa Y, Schueler M, et al. The mRNA-bound proteome and its global occupancy profile on protein-coding transcripts. Molecular cell 2012;46(5):674–690.
5. Castello A, Fischer B, Eichelbaum K, Horos R, Beckmann BM, Strein C, et al. Insights into RNA Biology from an Atlas of Mammalian mRNA-Binding Proteins. Cell 2012;149(6):1393–1406.
6. Gerstberger S, Hafner M, Tuschl T. A census of human RNA-binding proteins. Nature Reviews Genetics 2014;15(12):829–845.
7. Beckmann BM, Castello A, Medenbach J. The expanding universe of ribonucleoproteins: of novel RNA-binding proteins and unconventional interactions. Pflügers Archiv-European Journal of Physiology 2016;468(6):1029–1040.
8. Pereira B, Billaud M, Almeida R. RNA-Binding Proteins in Cancer: Old Players and New Actors. Trends in cancer 2017;3(7):506–528.
9. Nussbacher JK, Batra R, Lagier-Tourenne C, Yeo GW. RNA-binding proteins in neurodegeneration: Seq and you shall receive. Trends in neurosciences 2015;38(4):226–236.
10. Jankowsky E, Harris ME. Specificity and nonspecificity in RNA–protein interactions. Nature reviews Molecular cell biology 2015;16(9):533–544.
11. Hafner M, Landthaler M, Burger L, Khorshid M, Hausser J, Berninger P, et al. Transcriptome-wide Identification of RNA-Binding Protein and MicroRNA Target Sites by PAR-CLIP. Cell 2010;141(1):129–141.
12. Huppertz I, Attig J, D'Ambrogio A, Easton LE, Sibley CR, Sugimoto Y, et al. iCLIP: Protein–RNA interactions at nucleotide resolution. Methods 2014;65(3):274–287.
13. Van Nostrand EL, Pratt GA, Shishkin AA, Gelboin-Burkhart C, Fang MY, Sundararaman B, et al. Robust transcriptome-wide discovery of RNA-binding protein binding sites with enhanced CLIP (eCLIP). Nature methods 2016;13(6):508.
14. Lee FC, Ule J. Advances in CLIP technologies for studies of protein-RNA interactions. Molecular cell 2018;69(3):354–369.
15. Sugimoto Y, König J, Hussain S, Zupan B, Curk T, Frye M, et al. Analysis of CLIP and iCLIP methods for nucleotide-resolution studies of protein-RNA interactions. Genome biology 2012;13(8):R67.
16. König J, Zarnack K, Rot G, Curk T, Kayikci M, Zupan B, et al. iCLIP reveals the function of hnRNP particles in splicing at individual nucleotide resolution. Nature structural & molecular biology 2010;17(7):909–915.
17. Wheeler EC, Van Nostrand EL, Yeo GW. Advances and challenges in the detection of transcriptome-wide protein–RNA interactions. Wiley Interdisciplinary Reviews: RNA 2018;9(1):e1436.
18. Maticzka D, Ilik IA, Aktas T, Backofen R, Akhtar A. uvCLAP is a fast and non-radioactive method to identify in vivo targets of RNA-binding proteins. Nature communications 2018;9(1):1142.
19. Uhl M, Houwaart T, Corrado G, Wright PR, Backofen R. Computational analysis of CLIP-seq data. Methods 2017;118:60–72.
20. Bischler T, Maticzka D, Förstner KU, Wright PR, PEAKachu;. https://github.com/tbischler/PEAKachu.
21. Lovci MT, Ghanem D, Marr H, Arnold J, Gee S, Parra M, et al. Rbfox proteins regulate alternative mRNA splicing through evolutionarily conserved RNA bridges. Nature structural & molecular biology 2013;20:1434.
22. Krakau S, Richard H, Marsico A. PureCLIP: capturing target-specific protein–RNA interaction footprints from single-nucleotide CLIP-seq data. Genome biology 2017;18(1):240.
23. Chen B, Yun J, Kim MS, Mendell JT, Xie Y. PIPE-CLIP: a comprehensive online tool for CLIP-seq data analysis. Genome biology 2014;15(1):R18.
24. Maragkakis M, Alexiou P, Nakaya T, Mourelatos Z. CLIPSeqTools—a novel bioinformatics CLIP-seq analysis suite. RNA 2016;22(1):1–9.
25. Curk T, Rot G, Gorup u, Zmrzlikar J, Konig J, Sugimoto Y, et al. iCount: protein-RNA interaction iCLIP data analysis 2016;.
26. Afgan E, Baker D, Batut B, van den Beek M, Bouvier D, Čech M, et al. The Galaxy platform for accessible, reproducible and collaborative biomedical analyses: 2018 update. Nucleic acids research 2018;46(W1):W537–W544.
27. Gehman LT, Meera P, Stoilov P, Shiue L, O'Brien JE, Meisler MH, et al. The splicing regulator Rbfox2 is required for both cerebellar development and mature motor function. Genes & development 2012;.
28. Yeo GW, Coufal NG, Liang TY, Peng GE, Fu XD, Gage FH. An RNA code for the FOX2 splicing regulator revealed by mapping RNA-protein interactions in stem cells. Nature Structural and Molecular Biology 2009;16(2):130.
29. Singh RK, Xia Z, Bland CS, Kalsotra A, Scavuzzo MA, Curk T, et al. Rbfox2-Coordinated Alternative Splicing of Mef2d and Rock2 Controls Myoblast Fusion during Myogenesis. Molecular cell 2014;55(4):592–603.
30. Girardot C, Scholtalbers J, Sauer S, Su SY, Furlong EE. Je, a versatile suite to handle multiplexed NGS libraries with unique molecular identifiers. BMC bioinformatics 2016;17(1):419.
31. Smith TS, Heger A, Sudbery I. UMI-tools: Modelling sequencing errors in Unique Molecular Identifiers to improve quantification accuracy. Genome research 2017;.
32. Maticzka D, bctools;. https://github.com/dmaticzka/bctools.

33. Martin M. Cutadapt removes adapter sequences from high-throughput sequencing reads. EMBnet journal 2011;17(1):pp–10.
34. Andrews S, et al. FastQC: a quality control tool for high throughput sequence data 2010;.
35. Dobin A, Davis CA, Schlesinger F, Drenkow J, Zaleski C, Jha S, et al. STAR: ultrafast universal RNA-seq aligner. Bioinformatics 2013;29(1):15–21.
36. Langmead B, Salzberg SL. Fast gapped-read alignment with Bowtie 2. Nature methods 2012;9(4):357.
37. Hoffmann S, Otto C, Kurtz S, Sharma CM, Khaitovich P, Vogel J, et al. Fast mapping of short sequences with mismatches, insertions and deletions using index structures. PLoS computational biology 2009;5(9):e1000502.
38. Wysoker A, Fennell T, Ruan J, Homer N, Marth G, Abecasis G, et al. The Sequence alignment/map (SAM) format and SAMtools. Bioinformatics 2009;25:2078–2079.
39. Quinlan AR, Hall IM. BEDTools: a flexible suite of utilities for comparing genomic features. Bioinformatics 2010;26(6):841–842.
40. Ramírez F, Ryan DP, Grüning B, Bhardwaj V, Kilpert F, Richter AS, et al. deepTools2: a next generation web server for deep-sequencing data analysis. Nucleic acids research 2016;44(W1):W160–W165.
41. Uyar B, Yusuf D, Wurmus R, Rajewsky N, Ohler U, Akalin A. RCAS: an RNA centric annotation system for transcriptome-wide regions of interest. Nucleic acids research 2017;45(10):e91–e91.
42. Bailey TL, Boden M, Buske FA, Frith M, Grant CE, Clementi L, et al. MEME SUITE: tools for motif discovery and searching. Nucleic acids research 2009;37(suppl_2):W202–W208.
43. Love MI, Huber W, Anders S. Moderated estimation of fold change and dispersion for RNA-seq data with DESeq2. Genome biology 2014;15(12):550.
44. broadinstitute, Picard;. http://broadinstitute.github.io/picard.
45. Maticzka D, targetdist; 2016. https://github.com/dmaticzka/targetdist.git.
46. Guilmatre A, Huguet G, Delorme R, Bourgeron T. The emerging role of SHANK genes in neuropsychiatric disorders. Developmental neurobiology 2014;74(2):113–122.
47. Leblond CS, Heinrich J, Delorme R, Proepper C, Betancur C, Huguet G, et al. Genetic and functional analyses of SHANK2 mutations suggest a multiple hit model of autism spectrum disorders. PLoS genetics 2012;8(2):e1002521.
48. Giudice G, Sánchez-Cabo F, Torroja C, Lara-Pezzi E. ATtRACT – a database of RNA-binding proteins and associated motifs. Database 2016;2016.
49. Ray D, Kazan H, Cook KB, Weirauch MT, Najafabadi HS, Li X, et al. A compendium of RNA-binding motifs for decoding gene regulation. Nature 2013;499(7457):172.
50. Teplova M, Hafner M, Teplov D, Essig K, Tuschl T, Patel DJ. Structure–function studies of STAR family Quaking proteins bound to their in vivo RNA target sites. Genes & development 2013;27(8):928–940.
51. Wilbert ML, Huelga SC, Kapeli K, Stark TJ, Liang TY, Chen SX, et al. LIN28 binds messenger RNAs at GGAGA motifs and regulates splicing factor abundance. Molecular cell 2012;48(2):195–206.
52. Jungkamp AC, Stoeckius M, Mecenas D, Grün D, Mastrobuoni G, Kempa S, et al. In vivo and transcriptome-wide identification of RNA binding protein target sites;.
53. Maticzka D, Lange SJ, Costa F, Backofen R. GraphProt: modeling binding preferences of RNA-binding proteins. Genome biology 2014;15(1):R17.
54. Heyl F, Backofen R. StoatyDive: Evaluation and Classification of Peak Profiles for Sequencing Data. bioRxiv 2019;p. 799114.
55. Bottini S, Pratella D, Grandjean V, Repetto E, Trabucchi M. Recent computational developments on CLIP-seq data analysis and microRNA targeting implications. Briefings in bioinformatics 2018;19(6):1290–1301.
56. Davis MP, van Dongen S, Abreu-Goodger C, Bartonicek N, Enright AJ. Kraken: a set of tools for quality control and analysis of high-throughput sequence data. Methods 2013;63(1):41–49.
57. Ewels P, Magnusson M, Lundin S, Käller M. MultiQC: summarize analysis results for multiple tools and samples in a single report. Bioinformatics 2016;32(19):3047–3048.
58. Baruzzo G, Hayer KE, Kim EJ, Di Camillo B, FitzGerald GA, Grant GR. Simulation-based comprehensive benchmarking of RNA-seq aligners. Nature methods 2017;14(2):135.
59. Engström PG, Steijger T, Sipos B, Grant GR, Kahles A, Alioto T, et al. Systematic evaluation of spliced alignment programs for RNA-seq data. Nature methods 2013;10(12):1185.
60. Williams CR, Baccarella A, Parrish JZ, Kim CC. Empirical assessment of analysis workflows for differential expression analysis of human samples using RNA-Seq. BMC bioinformatics 2017;18(1):38.
61. Lihu A, Holban Ş. A review of ensemble methods for de novo motif discovery in ChIP-Seq data. Briefings in bioinformatics 2015;16(6):964–973.
62. Hashim FA, Mabrouk MS, Al-Atabany W. Review of Different Sequence Motif Finding Algorithms. Avicenna journal of medical biotechnology 2019;11(2):130.
63. Anders S, Pyl PT, Huber W. HTSeq—a Python framework to work with high-throughput sequencing data. Bioinformatics 2015;31(2):166–169.

Supplementary 1

Click here to access/download
Supplementary Material
Supplements_1.pdf

Supplementary 2

Click here to access/download
Supplementary Material
Supplements_2.pdf

Supplementary 3

Click here to access/download
Supplementary Material
Supplements_3.pdf

Supplementary 4

Click here to access/download
Supplementary Material
Supplements_4.pdf

bib style

Click here to access/download
Supplementary Material
vancouver-authoryear.bst

style

Click here to access/download
Supplementary Material
oup-contemporary.cls

Manuscript tex

Click here to access/download
Supplementary Material
main.tex

bib

Click here to access/download
Supplementary Material
paper-refs.bib
