# Supplementary material for: Galaxy CLIP-Explorer: a web server for CLIP-Seq data analysis
Source: Gigascience. 2020 Nov 11;9(11):giaa108. doi: 10.1093/gigascience/giaa108 (PMC7657819; doi:10.1093/gigascience/giaa108)
Supplement: giaa108_GIGA-D-19-00287_Revision_3 [file giaa108_giga-d-19-00287_revision_3.pdf]

GigaScience

Galaxy CLIP-Explorer: a web server for CLIP-Seq data analysis

--Manuscript Draft--

**Manuscript Number:**	GIGA-D-19-00287R3
**Full Title:**	Galaxy CLIP-Explorer: a web server for CLIP-Seq data analysis
**Article Type:**	Review
**Funding Information:**	Deutsche Forschungsgemeinschaft (322977937/GRK2344 2017 MeInBio -- BioInMe) — Mr. Florian Heyl<br>Deutsche Forschungsgemeinschaft (BA2168/11-2 SPP 1738) — Mr. Michael Uhl<br>Deutsche Forschungsgemeinschaft (TRR 167/1 2027 NeuroMac) — Dr. Rolf Backofen<br>Deutsche Forschungsgemeinschaft (SFB 992/2 2016) — Dr. Rolf Backofen
**Abstract:**	Background:<br><br>Post-transcriptional regulation via RNA-binding proteins (RBP) plays a fundamental role in every organism, but the regulatory mechanisms lack important understanding. Nevertheless, they can be fathomed by crosslinking immunoprecipitation in combination with high-throughput sequencing (CLIP-Seq). CLIP-Seq answers questions about the functional role of an RBP and its targets by determining binding sites on a nucleotide level and associated sequence and structural binding patterns. In recent years the amount of CLIP-Seq data skyrocketed, urging the need for an automatic data analysis that can deal with different experimental setups. However, noncanonical data, new protocols, and a huge variety of tools, especially for peak calling, made it difficult to define a standard.<br><br>Findings:<br><br>CLIP-Explorer is a flexible, and reproducible data analysis pipeline for iCLIP data that supports for the first time eCLIP, FLASH, and uvCLAP data. Individual steps like peak calling can be changed to adapt to different experimental settings. We validate CLIP-Explorer on eCLIP data, finding similar or nearly identical motifs for various proteins in comparison with other databases. In addition, we detect new sequence motifs for PTBP1, and U2AF2. Finally, we optimize the peak calling with three different peakcallers on RBFOX2 data, discuss the difficulty of the peak calling step and give advice for different experimental setups.<br><br>Conclusion:<br><br>CLIP-Explorer finally fills the demand for a flexible CLIP-Seq data analysis pipeline that is applicable to the up-to-date CLIP protocols. The paper further shows the limitations of current peak calling algorithms and the importance of a robust peak detection.
**Corresponding Author:**	Florian Heyl<br><br>GERMANY
**Corresponding Author Secondary Information:**	
**Corresponding Author's Institution:**	
**Corresponding Author's Secondary Institution:**	
**First Author:**	Florian Heyl
**First Author Secondary Information:**	
**Order of Authors:**	Florian Heyl

Powered by Editorial Manager® and ProduXion Manager® from Aries Systems Corporation

	Daniel Maticzka
	Michael Uhl
	Rolf Backofen
**Order of Authors Secondary Information:**	
**Response to Reviewers:**	Reviewer #2: I support publication and I think this will be a very useful tool!<br><br>Minor comment: the Nostrand et al. [13] callouts should be "Van Nostrand et al. [13]"<br><br>Answer: We have changed the callouts to the correct format.<br><br>We thank all reviewers for their time and input to complete the paper.
**Additional Information:**	
**Question**	**Response**
Are you submitting this manuscript to a special series or article collection?	No
**Experimental design and statistics**<br><br>Full details of the experimental design and statistical methods used should be given in the Methods section, as detailed in our Minimum Standards Reporting Checklist. Information essential to interpreting the data presented should be made available in the figure legends.<br><br>Have you included all the information requested in your manuscript?	Yes
**Resources**<br><br>A description of all resources used, including antibodies, cell lines, animals and software tools, with enough information to allow them to be uniquely identified, should be included in the Methods section. Authors are strongly encouraged to cite Research Resource Identifiers (RRIDs) for antibodies, model organisms and tools, where possible.<br><br>Have you included the information requested as detailed in our Minimum Standards Reporting Checklist?	Yes
**Availability of data and materials**	Yes

Powered by Editorial Manager® and ProduXion Manager® from Aries Systems Corporation

All datasets and code on which the conclusions of the paper rely must be either included in your submission or deposited in publicly available repositories (where available and ethically appropriate), referencing such data using a unique identifier in the references and in the “Availability of Data and Materials” section of your manuscript.<br><br>Have you have met the above requirement as detailed in our Minimum Standards Reporting Checklist?	

Manuscript
Click here to access/download;Manuscript;main.pdf

GigaScience, 2017, 1–11

doi: xx.xxxx/xxxx
Manuscript in Preparation
Technical Note

TECHNICAL NOTE

Galaxy CLIP-Explorer: a web server for CLIP-Seq data analysis

Florian Heyl[1,*], Daniel Maticzka[1], Michael Uhl[1] and Rolf Backofen[1,2,*]

[1]Bioinformatics Group, Department of Computer Science, University of Freiburg, Georges-Köhler-Allee 106, 79110 Freiburg, Germany and [2]Signalling Research Centres BIOSS and CIBSS, University of Freiburg, Schaenzlestr. 18, 79104 Freiburg, Germany

[*]heylf@informatik.uni-freiburg.de, backofen@informatik.uni-freiburg.de

Abstract

Background Post-transcriptional regulation via RNA-binding proteins (RBP) plays a fundamental role in every organism, but the regulatory mechanisms lack important understanding. Nevertheless, they can be fathomed by crosslinking immunoprecipitation in combination with high-throughput sequencing (CLIP-Seq). CLIP-Seq answers questions about the functional role of an RBP and its targets by determining binding sites on a nucleotide level and associated sequence and structural binding patterns. In recent years the amount of CLIP-Seq data skyrocketed, urging the need for an automatic data analysis that can deal with different experimental setups. However, noncanonical data, new protocols, and a huge variety of tools, especially for peak calling, made it difficult to define a standard.
Findings CLIP-Explorer is a flexible, and reproducible data analysis pipeline for iCLIP data that supports for the first time eCLIP, FLASH, and uvCLAP data. Individual steps like peak calling can be changed to adapt to different experimental settings. We validate CLIP-Explorer on eCLIP data, finding similar or nearly identical motifs for various proteins in comparison with other databases. In addition, we detect new sequence motifs for PTBP1, and U2AF2. Finally, we optimize the peak calling with three different peakcallers on RBFOX2 data, discuss the difficulty of the peak calling step and give advice for different experimental setups.
Conclusion CLIP-Explorer finally fills the demand for a flexible CLIP-Seq data analysis pipeline that is applicable to the up-to-date CLIP protocols. The paper further shows the limitations of current peak calling algorithms and the importance of a robust peak detection.

Key words: CLIP-Seq; Data Analysis; Galaxy; RNA; Protein

Findings

Background

RNA plays a fundamental role in many regulatory processes like splicing or translation. Yet, processes like translation also undergo regulatory steps involving proteins such as elongation factors. These RBPs (RNA-binding proteins) interact with their target RNA and form ribonucleoprotein complexes [1]. Studies have revealed the involvement of RBPs in stages like splicing, polyadenylation, localization, translation, stability, and degradation [2, 3, 4, 5]. So far more than a thousand RBPs have been identified in human cells [2, 6, 7]. Various RBPs have been linked to neurodegenerative diseases and various types of cancer [2, 4, 8, 9]. These observations emphasize the importance to explore the mechanisms behind the regulatory processes mediated by RBPs.

Crosslinking and immunoprecipitation (CLIP) facilitates the analysis of the interdependence between the proteome and transcriptome *in vivo* [10] by detecting binding sites for RBPs on a genome-wide level. Many CLIP protocols such as PAR-CLIP [11], iCLIP [12], or eCLIP [13] emerged over a short period of time and new methods are still in development [14]. All methods consist of three fundamental steps: crosslinking the

Compiled on: August 6, 2020.
Draft manuscript prepared by the author.

RBP of interest to its target RNAs, purification and immunoprecipitation of the resulting complexes, and high-throughput sequencing of the resulting RNAs. Despite these commonalities, protocols such as iCLIP or eCLIP perform additional steps to increase the precision of the CLIP-Seq experiment [13, 15, 16, 17], which have to be covered by additional analysis tasks. For example, iCLIP introduced random barcodes (unique molecular identifiers, short UMIs) to reduce the number of duplicated reads [12]. Protocols like eCLIP [13] and uvCLAP [18] adapted this procedure. A deduplication step is therefore imperative for iCLIP, eCLIP, FLASH, and uvCLAP [12, 19].

Because of the complexity and variety of CLIP protocols, the computational analysis is still the critical bottleneck, both in time and reproducibility. Individual tools that perform quality control, mapping, peak calling, and motif detection for CLIP-Seq data exist. However, an automatic and complete data analysis pipeline has to deal with a big list of obstacles such as biases that are introduced by the CLIP-Seq protocol and experimental conditions. On top of this, additional problems arise from changing hardware and tool versions, practicality of the user interface, different library formats (e.g., biological replicates or multiplexed data), and different CLIP-Seq data formats for old, recent, or upcoming protocols. Furthermore, each tool for each subtask has different assumptions and parameters that need to be optimized for the underlying protocol [19]. The most challenging task is the binding site identification, where a couple of different peakcallers, such as Piranha [1], PEAKachu [20], CLIPper [21], and PureCLIP [22], exist. For example, biological replicates are not supported by some peakcallers like Piranha [1]. These obstacles lead to a lack of reproducibility for the CLIP-Seq data analysis.

One possible solution could be one big, but fixed pipeline that can cope with every possible type of data. This solution was already tried in the case of PIPE-CLIP [23] or CLIPSeqTools [24]. Nevertheless, it is intractable to cover all possible combinations of different experimental settings, such as the number of replicates, the existence of a control library and others. Focussing instead on one specific type of data is easier to handle, like analyzing only iCLIP data with iCount [25]. However, neither PIPE-CLIP, nor CLIPSeqTools and iCount can be quickly and simply expanded or modified. They lack the option for an extension to cover noncanonical experimental data or new CLIP-Seq data types such as eCLIP, FLASH, or uvCLAP.

We hereby present CLIP-Explorer (https://clipseq.usegalaxy.eu/), a CLIP-Seq pipeline implemented in Galaxy [26]. CLIP-Explorer provides all necessary tools to analyze eCLIP, FLASH, uvCLAP and iCLIP data. CLIP-Explorer is well documented through an online tutorial in the Galaxy training material (https://galaxyproject.github.io/training-material/topics/transcriptomics/tutorials/clipseq/tutorial.html) and the main domain. Both websites assist the user to understand the main steps and parameters of the pipeline and the featured tools. The user can then, for example, replace the peakcaller or read mapper. It is not required to have detailed knowledge about the tools. CLIP-Explorer works in a server environment, thus the user does not have to worry about varying hardware or tool versions. A constant maintenance of CLIP-Explorer makes the data analysis easy to reproduce.

We have validated CLIP-Explorer on eCLIP data of DROSHA, HNRNPK, IGF2BP1, KHDRBS1, LIN28B, PTBP1, QKI, SLBP and U2AF2. We compared the results with a different analysis pipeline and databases, finding great diversity in the number of predicted peaks and found motifs. A more comprehensive analysis including the peakcallers Piranha, PureCLIP, and PEAKachu was done for RBFOX2 [13] as it has well documented targets and motifs. The protein RBFOX2 encoded by the gene RBM9 is a tissue-specific splicing factor involved in developmental processes [21, 27]. Studies have shown RBFOX2's binding preference for introns close to differentially spliced exons [28, 29]. The conserved sequence motif *TGCATG* has been shown to be enriched in RBPFOX2's binding sites [21, 28, 29]. Concerning the inconsistent results of the peak calling, we propose standard guidelines for the peak calling for different experimental setups. We confirm RBFOX2's binding characteristics from the literature as another validation for CLIP-Explorer.

CLIP-Explorer: A Versatile Pipeline for the Analysis of CLIP-Seq Data

Different experimental settings require different analysis pipelines, since pre-processing, mapping, peak calling and motif detection have to be adapted. For that reason, CLIP-Explorer integrates several pipelines for analyzing different protocols, namely eCLIP, iCLIP, FLASH and uvCLAP. Common to all pipelines in CLIP-Explorer is the division into four major steps (Figure 1). In the pre-processing, the read library is demultiplexed and, if necessary, adapter sequences as well as inline barcodes and UMIs are removed. In the post-processing, the reads are aligned and deduplicated. CLIP-Explorer then identifies differentially enriched regions (peaks) that are further analyzed according to genomic localization and other criteria to investigate the precise function of the protein and properties of its targets. All subtasks are accompanied by quality control steps. The versatility of CLIP-Explorer allows the user to select three different peak calling pipelines for three different data specifications. The methods section covers CLIP-Explorer in more detail. Additional information can be found in the Galaxy training material.

Recommendations for PEAKachu, Piranha, and PureCLIP

To this day, a benchmarking dataset for CLIP-Seq data analysis does not exist because of missing experimental methods to verify predicted binding sites. It is therefore recommended to test more than one peakcaller and to test more than one parameter set, which is easily possible with CLIP-Explorer.

The newest version of PEAKachu [20] is well-suited for an experimental setup with at least two replicates for the CLIP experiment and at least two replicates for the control experiment, because it uses DESeq2 [43]. It is therefore best to turn on the DESeq2 normalization. If the user has less replicates, the user can still use PEAKachu, but DESeq2 requires at least two replicates for both experiment and control to calculate p-values. With less than two replicates, PEAKachu filters the peaks based on the fold change and the mad (median absolute deviation) multiplier. It is therefore wise to check the peaks with another peakcaller or peak calling pipeline. PEAKachu works best in the adaptive mode with a mad multiplier of 0.0, a log2 fold change threshold of 2.0, and an adjusted p-value threshold of 0.05. PEAKachu needs the parameter of the maximum insert size, identified beforehand by Picard [44]. The estimation of the insert size is only necessary if the user provides paired-end read data. The window mode of PEAKachu is not recommended, because it is rather unstable. The mad multiplier might reduce the number of peaks, as it works as a second cutoff. To get the full peak set, it is wise to leave it at zero and filter the peaks by the log2 fold change together with the adjusted p-value. One key parameter is the minimum block overlap that has to be tested with the default of 0.5 in the beginning. The user has to increase this parameter if the results show a lot of peaks in a close vicinity. Another critical parameter is the minimum cluster expression fraction and the minimum block expression. These parameters can change the total number of

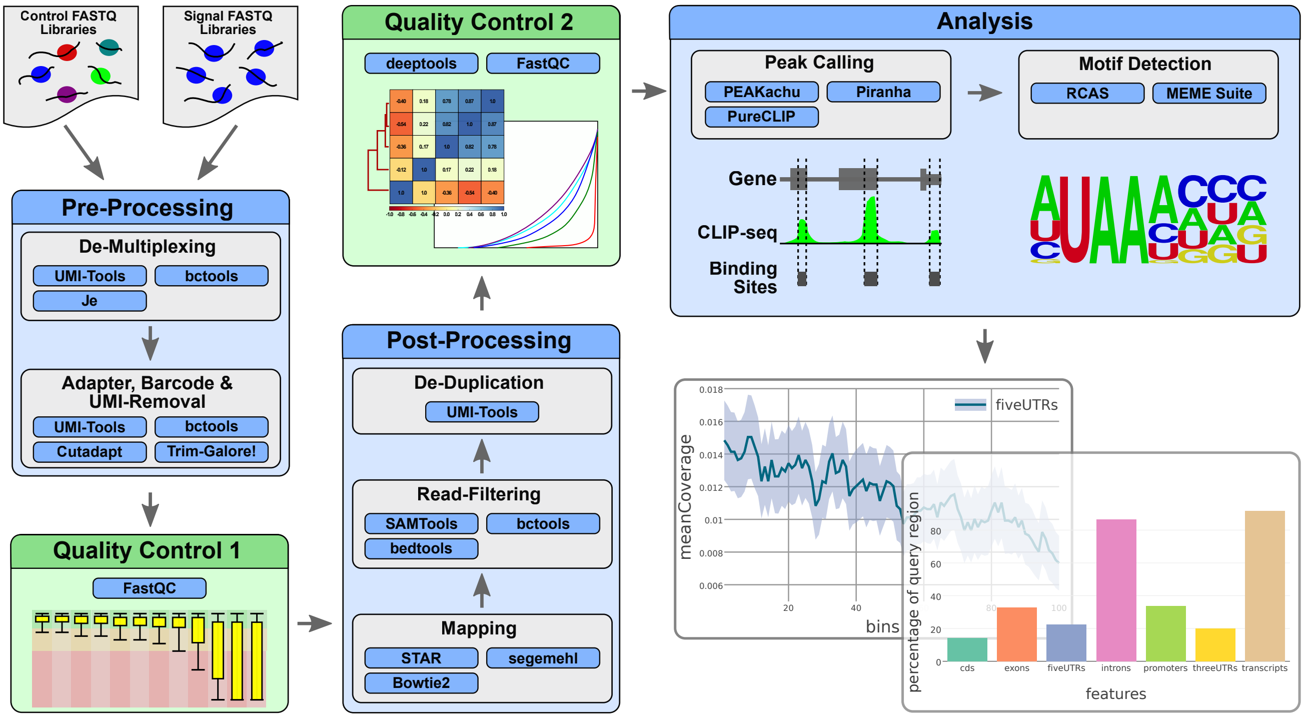

Figure 1. Flowchart of CLIP-Explorer; CLIP-Explorer has three major steps. In the pre-processing, the read library is demultiplexed, if necessary, and adapter sequences as well as in-line barcodes and UMIs are removed. CLIP-Explorer uses Je [30], UMI-Tools [31], bctools [32], and Cutadapt [33] for that purpose. A quality control step using FastQC [34] follows the pre-processing. In the post-processing, the reads are aligned with STAR [35], Bowtie [36] or segemehl [37], filtered using SAMtools [38], bedtools [39], and bctools, and deduplicated with UMI-tools. Another quality control, mainly with deeptools [40], checks the batch quality. Finally, CLIP-Explorer identifies differentially enriched regions using either PEAKachu [20], Piranha [1], or PureCLIP [22]. The binding regions are then analyzed with RCAS [41] and MEME-ChIP [42].

predicted peaks. Leave them in default with 0.01 and 0.1 and adjust the parameters if some interesting binding regions are not covered by PEAKachu's prediction. PEAKachu can be applied to iCLIP, eCLIP, FLASH, uvCLAP and even older protocols like PAR-CLIP.

Piranha [1] can be applied to an experimental setup with or without control, but it does not support replicates for the CLIP experiment. Each replicate has to be treated separately or further validated with a robust peak detection. However, if the user has only one replicate and no control, we recommend to use Piranha. If a control is provided, Piranha uses a zero-truncated negative binomial regression by default. Without a control it is wise to stick to a negative binomial. The distance to merge significant bins is one of the most crucial parameters of Piranha, similar to the minimum block overlap of PEAKachu. If the user observes a lot of peaks in a close vicinity, then this parameter has to be increased (e.g, 10). The bin size of the signal and control is another crucial parameter of Piranha that needs to be optimized. If the bin size is quite big (e.g., 200), then Piranha might miss a few good candidates. If the bin size is very small (e.g., 5), then Piranha predicts a lot of false positives. Piranha can also be applied to iCLIP, eCLIP, FLASH, uvCLAP, and even older protocols like PAR-CLIP.

PureCLIP [22] can be applied to an experimental setup with or without control, but it does not support replicates by the time of our analysis. It is therefore best to apply PureCLIP, as well as Piranha, to each replicate separately and find robust peaks by intersection, merging or calculating an IDR. Therefore, we recommend to use PureCLIP if the user has only one replicate for the CLIP and control experiment. PureCLIP already incorporates two default parameters sets. One set can be used if the protein is assumed to bind low complex motifs, which results in more broader and unspecific binding sites. PureCLIP predicts not only the binding region, but also the crosslinking sites. In our tests, PureCLIP quite often reported very small binding regions, almost identical to the crosslinking sites. It is therefore recommended to slightly extend the predicted binding sites (e.g, five to ten bases to the left and right) to cover the whole binding region. Furthermore, if the user provides paired-end reads, the mate containing the crosslinking event has to be provided explicitly. For iCLIP, FLASH, and uvCLAP this corresponds to the first mate, while for eCLIP it is the second mate. PureCLIP was specifically designed for eCLIP and iCLIP [22]. We recommend to use the peakcaller only for those protocols or other variants such as FLASH or uvCLAP.

It is not easy to find a standard peak calling algorithm with a standard parameter set. We tried to cover possible cases and recommendations for Piranha, PEAKachu, and PureCLIP, but these tools can change over time, or a new peakcaller might outrank them. The user can therefore find permanently updated recommendations and guidelines for a CLIP-Seq data analysis on CLIP-Explorer's main domain.

Effects of Using Different Peakcallers for RBFOX2

We use eCLIP data from DROSHA, HNRNPK, IGF2BP1, KHDRBS1, LIN28B, PTBP1, QKI, RBFOX2, SLBP, and U2AF2 from the study by Van Nostrand et al. [13] to validate CLIP-Explorer (see Methods). We first use the data of RBFOX2 to check the robustness and quality of the predicted binding sites of CLIP-Explorer, since the protein has a well-known binding motif. We intersect the peaks of Piranha, PEAKachu, and PureCLIP with bedtools [39] (see Methods). Piranha detects the highest number of potential binding regions for RBFOX2 (Figure 2a). Yet, more than one third of Piranha's peaks are not included in PEAKachu's and PureCLIP's peak set. PureCLIP has the highest fraction of peaks shared with the other two peakcallers, but it also has the lowest total number of peaks. PEAKachu on the other hand also has a high number of individual peaks, but significantly less

than Piranha. To check for the origin of the difference in the number of peaks, we compare all three peakcallers (PEAKachu, Piranha, PureCLIP), including the CLIPper peaks of the study by Van Nostrand et al. [13] for RBFOX2, regarding potential CLIP-Seq artifacts and biases. The table 1 shows that less than 1% of the peaks from all peakcallers overlap with 3' or 5' UTRs. PEAKachu has 362 peaks that overlap with repeats (2.45%), however all peakcallers have a rate below 1% of peaks that overlap with intron repeats or any RNA pseudogene region. We also check the peak length and the distance between the peaks of the different peakcallers (Supplementary Table 3). PEAKachu has on average larger peaks with 131 nucleotides. On the other hand, PureCLIP has the smallest peaks with an average of 36 nucleotides. The distribution of Piranha is a constant of 20 nucleotides because the peak length is a parameter the user defines for the tool. Furthermore, the distance between the

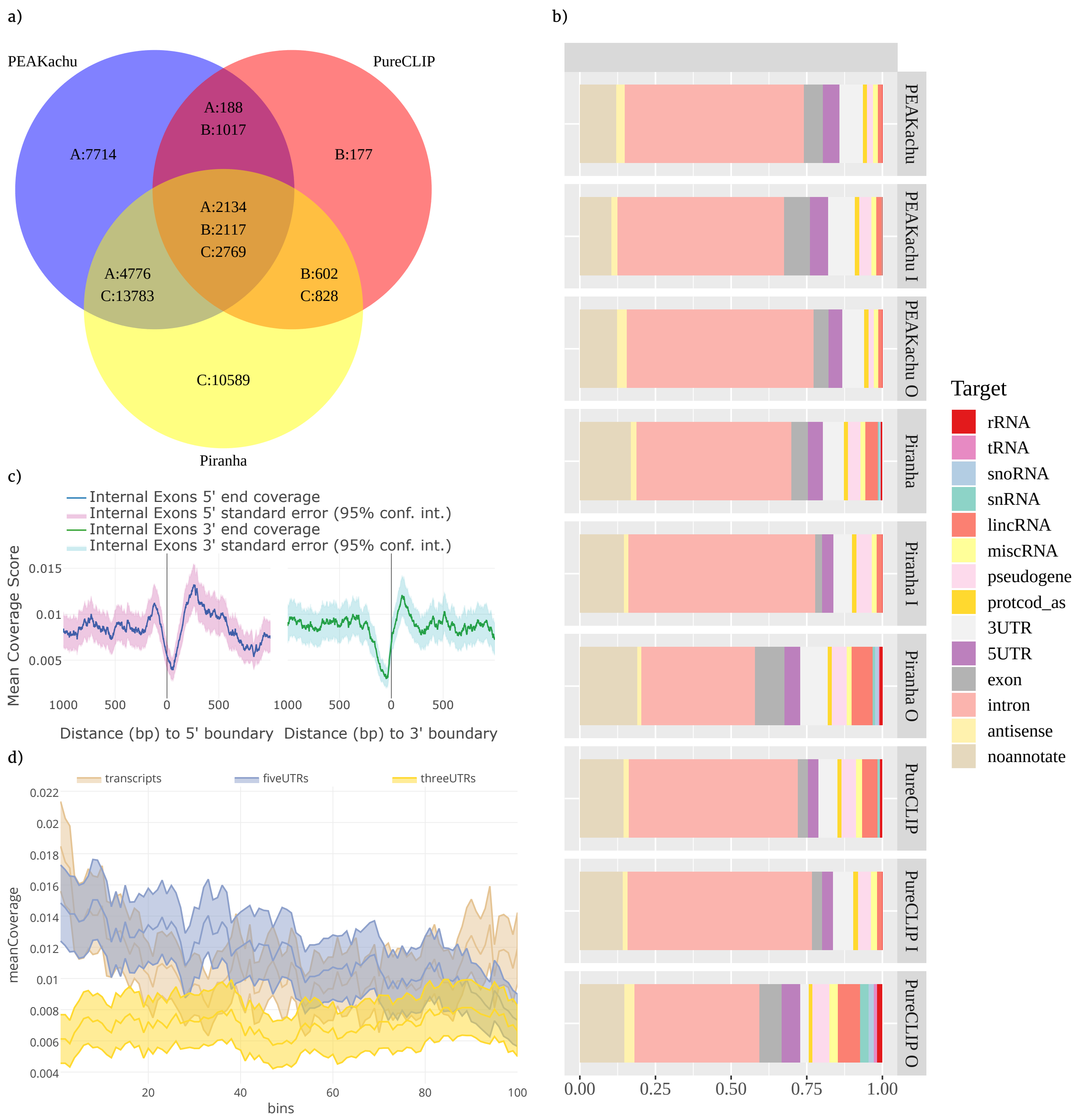

Figure 2. Comparison and analysis of the binding regions detected by PEAKachu, Piranha, and PureCLIP. The results are from the eCLIP data of RBFOX2 [13]. **(a)** We intersected the binding regions identified by the peakcallers with bedtools [39], paying attention to the strand (intersect -s), to assess the robustness of each method. **(b)** We then annotated the binding regions of each peakcaller and plotted the fraction of each target. We also analyzed separately the peak set of the individual peakcallers (O = peak set 7714/177/10589) and the peak set of the intersection with all peakcallers (I = peak set 2134/2117/2769). RBFOX2 prevalently binds introns, but also 3' and 5' UTRs as well as lincRNAs. The plot was generated with the hg19 script of targetdist [45]. Investigating the mean coverage of these binding regions identified by **(c)** PEAKachu [20] reveals an occupancy drop around the 5' and 3' ends of the exons. **(d)** Looking further at the mean coverage of the binding regions identified by PEAKachu in the overall transcript as well as for the 5' and 3' UTRs. The thickness of the ribbon around the mean coverage indicates the 95% confidence interval (mean +- standard error of the mean times 1.96). Each feature is divided into 100 bins of equal length, whereas features smaller than 100 bp are excluded [41].

Table 1. Percentage of peaks of the RBFOX2 peak set overlapping with the stated feature (P-genes = Pseudogenes).

Feature	PEAKachu	Piranha	PureCLIP	CLIPper
3'UTR	0.42	0.02	0.03	0.09
5'UTR	0.96	0.05	0.03	0.27
Repeats	2.45	0.01	0.15	0.87
Intron Repeats	0.04	0.00	0.00	0.00
ncRNA P-genes	0.09	0.38	0.59	0.01
rRNA P-genes	0.00	0.07	0.05	0.00
tRNA P-genes	0.00	0.00	0.00	0.00

peaks is almost identical between the peakcallers PEAKachu, PureCLIP, and CLIPper (Supplementary Table 3). Only Piranha has slightly more peaks, which are close together.

To identify the type of bound genomic regions, we annotate the peaks discovered by the three different peakcallers. The distributions of binding sites generated by these three tools show a similar trend and prevalence for introns as the main target of RBFOX2 (Figure 2b). Yet, introns are not the only target. Our findings suggest that some binding regions of RBFOX2 lie in 3' and 5' UTRs, but this fraction is not as big as for introns. PEAKachu, Piranha, and PureCLIP further detect another chunk of target sites in lincRNAs, but the portion detected by PEAKachu is smaller, probably because of a low coverage of lincRNAs in CLIP protocols. In addition, we annotated separately the peak set of the individual peakcallers (7714/177/10589, from Figure 2a) and the peak set of the intersection with all peakcallers (2134/2117/2769). The target distribution of Piranha and PureCLIP changes for the individual peak set. The portion of intron coverage shrinks and the portion exon targets increases as well as lincRNA, rRNA, snoRNA, and snRNA. In contrast, the target distributions of PEAKachu looks similar. However, the target distribution of the intersection of all three peakcallers looks different in comparison to the distribution of all peaks. PEAKachu's distribution has a smaller intron and a bigger exon and pseudogene portion. On the other hand, the peaks from the intersection set of Piranha and PureCLIP overlap more with introns and less with exons or any snRNA, snoRNA, tRNA, or rRNA.

To verify that RBFOX2 is a splicing factor, we investigate the peak profile of the binding sites by looking at the coverage plot of RBFOX2. PEAKachu depict a drop in the binding region coverage around the exon-intron-boundaries at the 5' and 3' ends (Figure 2c). The drop is more intense for the sites found by PEAKachu. Checking the mean coverage of the binding regions (Figure 2d), the results suggest a binding prevalence of RBFOX2 in the upstream region of the transcripts. Furthermore, RBFOX2 seems to target the beginning of the 5' UTR. In contrast, the binding coverage is homogeneous for the 3' UTR. The sequence *UGCAUG* seems to play an important part for the binding of RBFOX2 as it is among the top five motifs detected by the PEAKachu, Piranha and PureCLIP pipeline (see Table 2). The second motif shows guanine richness and the third motif cytosine and uracil richness for all peakcallers.

At the end, we check the function of RBFOX2 to clarify the role in human liver cancer cells (Hep G2). Looking at the top one hundred genomic regions that have RBFOX2 binding sites, we find Shank2 and Shank3 among the top hits as potential targets. Furthermore, a gene ontology (GO) analysis with RCAS for the targets of RBFOX2 identifies the protein to be relevant for the regulation of RNA splicing (with a Benjamini-Hochberg (BH) adjusted p-value of $< 10^{-4}$), as well as regulation of transcription by RNA polymerase I (BH adjusted p-value $< 10^{-3}$). In addition, the GO analysis identifies RBFOX2 to be involved in the regulation of histone modifications (BH adjusted p-value $< 10^{-4}$), nucleosome and nucleosomal binding (BH adjusted p-value $< 10^{-4}$ and 0.04, respectively) and methyl-CpG binding (BH adjusted p-value < 0.04).

Checking the results for RBFOX2, Piranha found the highest number of peaks. A lot of these peaks, however, might represent false positives since Piranha was executed without the information from the control experiments (see Methods). More than one third of Piranha's peaks were not included in PEAKachu's and PureCLIP's peak set, which endorses the supposition. The observation is also substantiated by the change of the target distribution of the Piranha peaks that do not intersect with the peaks of the other two peakcallers. These peaks might cover false positives that lie in unspecific regions such as rRNAs. Furthermore, the distance between the peaks is almost identical between the peakcallers. Only Piranha has slightly more peaks, which are closer together indicating a higher number of false positives because it calls many peaks (local maxima) in close proximity and does not combine them into a bigger peak (global maximum). In contrast, PureCLIP had the lowest number of predicted peaks and the highest fractions of peaks shared with the other peakcallers. This indicates that PureCLIP selects the peaks based on very stringent criteria. However, PureCLIP might therefore also have a high false negative rate. PureCLIP calls peaks for each replicate separately, which is likely the reason for missing some good candidates that have been jointly found by PEAKachu. Furthermore, the change of the target distribution for the PureCLIP peaks that do not intersect with the peaks of the other two peakcallers might show possible false positives. Like Piranha's distribution, peaks might overlap with unspecific regions such as rRNAs. PEAKachu, on the other hand, had the largest peaks. We cannot make a general judgement about a good value for the peak length, because each RBP and each binding region can be specific and a benchmarking dataset is missing for CLIP-Seq data. PEAKachu's target distribution for peaks that do not intersect with the peaks of the other two peakcallers might suggest some false positives, because of the bigger portion of peaks in pseudogenes in that peak set. We also checked for potential CLIP-Seq biases (annotation from Ensembl) to answer the question of the different number in peaks, but we could not find any evidence regarding a significant UTR or repeat region bias for any of the implemented peakcallers.

Despite the disparate numbers of peaks between PEAKachu, Piranha, and PureCLIP, the main motif of RBFOX2 with the sequence *UGCAUG* was identified with CLIP-Explorer for all used peakcallers. The motif seems to be very robust to varying peak calling conditions, which is endorsed by the fact that it can be even found with PureCLIP with a different pre-processing (see Table 2). We tested the peakcallers PEAKachu, Piranha, and PureCLIP on the alignments files from the study by Van Nostrand et al. [13]. With CLIP-Explorer all three peakcallers found the main motif with lower background noise as in comparison to the already processed alignments files from ENCODE. Furthermore the motif set from CLIP-Explorer looks more similar between all three peakcallers (see Table 2). It is therefore possible to find a better ground truth, that is to say, a benchmark set with CLIP-Explorer, since additionally more than one peakcaller can be tested with just a few clicks.

We further tried to verify RBFOX2's known role as splicing factor and that it preferably binds to introns [21, 27, 28, 29] to substantiate the credibility and versatility of CLIP-Explorer. The distributions of binding sites generated by PEAKachu, Piranha, and PureCLIP showed a prevalence for introns as the main target of RBFOX2 in accordance with the literature [28, 29], which is further supported by the target distribution of the intersected peaks for all three peakcallers. The drop of the binding occupancy of RBFOX2 around the exon-intron boundaries can also be seen in other studies [28, 29]. The binding coverage of RBFOX2 around the exon-intron-boundaries

Table 2. Top five RBFOX2 sequence motifs for each peakcaller identified by MEME-ChIP with E-value and the fraction of sequences with that specific motif, based on the RBFOX2 eCLIP data.

Pre-processing CLIP-Explorer			Pre-processing Van Nostrand et al. [13] (ENCODE)		
PEAKachu with 14,812 peaks	Piranha with 27,969 peaks	PureCLIP with 3,913 peaks	PEAKachu with 19,254 peaks	Piranha with 214,506 peaks	PureCLIP with 38,734 peaks
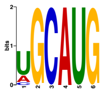	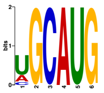	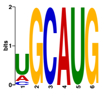	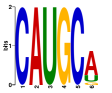	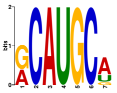	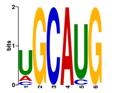
6.1e-702; 37.21%	6.6e-381; 16.43%	7.7e-288; 40.20%	1.3e-1052; 39.61%	2.1e-983; 5.37%	4.7e-2137; 34.07%
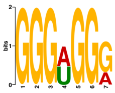	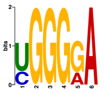	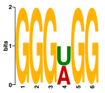	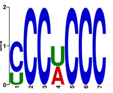	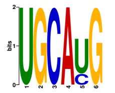	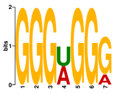
3.2e-075; 30.91%	1.4e-065; 16.50%	4.1e-030; 27.17%	2.2e-118; 30.00%	5.9e-366; 3.90%	2.9e-215; 13.51%
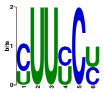	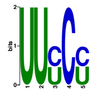	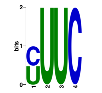	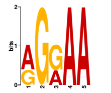	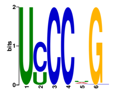	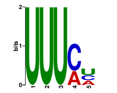
2.7e-049; 22.19%	3.2e-057; 18.30%	8.2e-009; 18.43%	1.7e-083; 34.65%	2.2e-329; 13.75%	1.4e-107; 16.41%
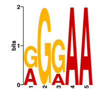	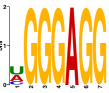	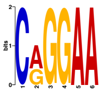	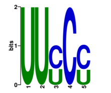	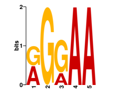	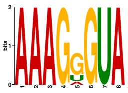
6.2e-045; 33.57%	1.4e-038; 10.32%	7.6e-006; 2.79%	1.3e-058; 33.98%	6.9e-298; 14.15%	2.1e-052; 0.67%
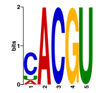	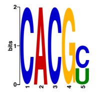	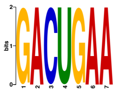	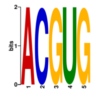	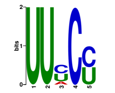	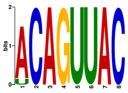
1.3e-027; 7.44%	2.2e-031; 7.57%	3.0e-005; 0.97%	2.5e-046; 5.50%	4.2e-146; 11.52%	4.7e-049; 0.56%

suggests an involvement of RBFOX2 in the regulation of splicing. The GO term analysis corroborates the hypothesis linking RBFOX2 to various splicing and structure-related processes, such as histone modifications. Besides, the Shank gene family members, as a potential target of RBFOX2, play an important part in neuronal functions, where alterations in the encoded proteins may be connected to autism [21]. Shank2 and Shank3 might hereby be regulated by alternative splicing [46, 47]. Another study has found the same interdependence of RBFOX2 and the Shank protein family [21].

Comparison of CLIP-Explorer's Results

We compare the sequence motifs detected by CLIP-Explorer with two different databases [48, 49], and then with the peaks identified in the study by Van Nostrand et al. [13] (Supplementary Table 1). Here, the CLIPper algorithm [21] was used to identify potential binding regions of the same proteins. For the CLIPper peaks, we predict the sequence motifs with MEME-ChIP in the same way as implemented in CLIP-Explorer.

As a first step, we focus on the sequence motifs that resulted from CLIP-Explorer using PEAKachu for peak calling. The motifs are similar and sometimes nearly identical to the motifs listed in the databases for HNRNPK, KHDRBS1, PTBP1, QKI, RBFOX2, and U2AF2 (Supplementary Table 1). For example, the QKI-motif *ACUAA* [50] or the known motif *UGCAUG* of RBFOX2 [21, 28, 29] can be found in the databases and is also detected by CLIP-Explorer and CLIPper. Some proteins such as DROSHA, LIN28B, and SLBP are not listed in the databases, and the proteins IGF2BP1 and RBFOX2 have only one or two motifs. CLIP-Explorer identifies new motifs for these proteins. Several of the new motifs detected by CLIP-Explorer are also detected by CLIPper but not covered by the databases [48, 49] or any other literature to the best of our knowledge (Supplementary Table 1). For example, we find the new sequence motif *CAGGCUGG* for PTBP1, or the motif *ACAG* for U2AF2.

For some proteins, such as DROSHA and HNRNPK, the motifs detected by CLIP-Explorer deviate from the corresponding CLIPper motifs, but still show similar sequence compositions (Supplementary Table 1). In addition, CLIPper slightly misses the known motif *GGAGA* of LIN28B [51].

For a more fine-grained comparison between the PEAKachu pipeline in CLIP-Explorer and the CLIPper pipeline in Van Nostrand et al. [13], we intersect the peaks for each protein with bedtools [39] to check for common binding sites. We require an overlap for bedtools of at least one base (see Methods). This comparison reveals a discrepancy between some proteins. Less then ten percent of all PEAKachu peaks overlap with the CLIPper peaks of the proteins KHDRBS1 (Supplementary Table 2). Quite often, PEAKachu finds more peaks in comparison to CLIPper, except for QKI and U2AF2. For example, CLIPper finds 161 peaks and CLIP-Explorer (PEAKachu) 220 for the protein SLBP. Using PEAKachu, CLIP-Explorer predicts nearly sixty more peaks than the CLIPper pipeline. We suspect that this difference is due to the computational model of CLIPper, which calls peaks for every replicate separately. PEAKachu, on the other hand, calls with all replicates in mind. Therefore, we investigate the difference in the CLIPper peak sets for each replicate. The first replicate encompasses 7,942 CLIPper peaks and the second replicate 1,530 peaks, when we filter for entries with a p-value of 0.05 and a log2 fold change of 1, which makes a total difference of 6,412 peaks. Intersecting the peaks without an irreducible discovery rate (IDR) results in 291 peaks, more than PEAKachu, and with an IDR CLIPper has the aforementioned 161 peaks, so less than PEAKachu. Thus, roughly 20% of the CLIPper peaks in replicate one are contained in replicate two. Consequently, the difference in the peak set of PEAKachu (CLIP-Explorer) might come from a different noise estimation between replicates, thus detecting other binding sites. Because of the different number of peaks, we check for potential CLIP-

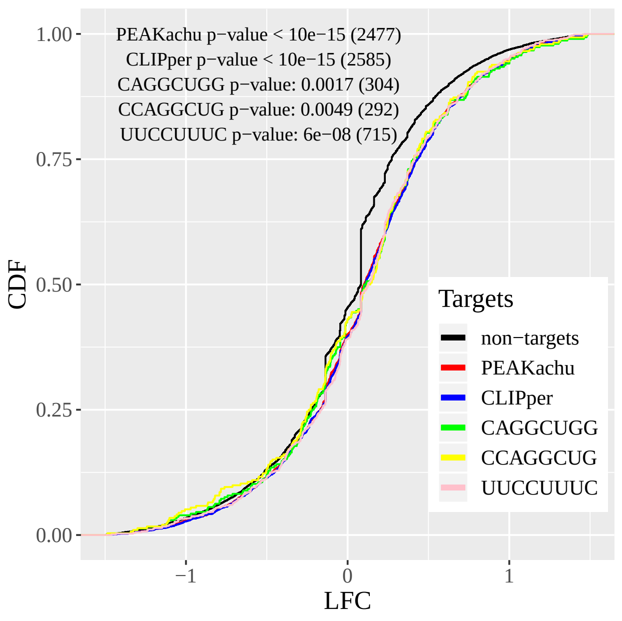

Figure 3. Cumulative density function (CDF) of the log2 fold changes of the genes of hg38 for a knockdown experiment of PTBP1; The identified targets of CLIPper and PEAKachu have nearly identical trends and show a significantly shifted CDF (p-values of one-sided t-test in the plot), as well as the targets with the identified motifs *CAGGCUGG* (CLIP-Explorer), *CCAGGCUG* (CLIPper), and *UUCCUUUC* (CLIP-Explorer and CLIPper) with p-values of one-sided Wilcoxon test, in comparison to the non-targets. The number of targets are listed after the p-value.

Seq biases (Supplementary Table 4). Both PEAKachu and CLIPper have no peaks in repeat or pseudogene regions, except for one PEAKachu peak in a ncRNA pseudogene. PEAKachu also finds more peaks that overlap with ncRNA genes (48 peaks) than CLIPper (17 peaks). We also check the number of peaks overlapping with histone genes since SLBP targets mainly histone RNAs. PEAKachu finds slightly more peaks in histone regions (163 peaks) than CLIPper (135 peaks). Consequently, PEAKachu covers slightly more peaks in histone regions, but also more peaks in ncRNAs genes, which might represent false positives since SLBP is known to target histone mRNA. The eCLIP pipeline from Van Nostrand et al. [13] already removed repeat regions in a double mapping approach.

To check the correctness of the identified new motifs such as *CAGGCUGG* for PTBP1, we took independent RNA-Seq data from ENCODE from a knockdown and control experiment for PTBP1 (see Methods). We intersected the identified binding sites of PTBP1 from CLIP-Explorer (PEAKachu) and CLIPper with the genes of hg38 and calculated the log2 fold change of all genes. Figure 3 clearly shows a significant shift in the cumulative density function (CDF) of the fold changes. This observation was expected since true binding sites should have different RNA rates in a knockdown experiment. Thus, the identified sites from PEAKachu and CLIPper might be real binding sites of PTBP1 as both peakcallers show similar CDFs even though the processing of the data was different. Thus we checked the motif *CAGGCUGG* (from CLIP-Explorer), *CCAGGCUG* (from CLIPper), and another motif *UUCCUUUC* (from CLIP-Explorer and CLIPper). All three CDFs are significantly shifted. Consequently, *CAGGCUGG* might not be a false positive.

Potential Implications

CLIP-Explorer is a valuable tool for researchers working with CLIP-Seq data as it simplifies and integrates many processing steps in a well-tested and optimized pipeline. CLIP-Explorer provides the user with an extensive overview of the potential function of the RBP and its target RNAs. It can be easily extended or modified, and has no installation overhead as it is integrated in Galaxy. CLIP-Explorer is thus the first general and fully automatic pipeline for eCLIP, FLASH, and uvCLAP data, which can also be applied to iCLIP and other types of CLIP-Seq protocols. Besides, it is permanently maintained, and new tools can be implemented or exchanged with existing ones to warrant a highly efficient data analysis.

We analyzed different eCLIP datasets and compared our findings with different databases [48, 49] and the results from the study by Van Nostrand et al. [13]. The analysis of different proteins, such as DROSHA, HNRNPK, and in more detail RBFOX2, showed the strength of the flexibility provided by CLIP-Explorer. We could identify similar and even new sequence motifs for PTBP1 with the motif *CAGGCUGG*, and U2AF2 with *ACAG*. We also verified the sequence motif *UGCAUG* for RBFOX2 and found other guanine, cytosine and uracil-rich binding regions. The most significant sequence motif *UGCAUG* of RBFOX2 was found by Piranha, PEAKachu, and PureCLIP, even though the three peakcallers predicted three substantially different peak sets. Based on our results we recommend to test more than one peak calling algorithm for other RBPs to assess the robustness of the motifs. We showed that different peak calling tools might result in different peak sets and thus encompass different false positives and negatives. Yet, CLIP-Explorer allows this very easily, because of its user-friendly interface. An exchange of the peakcaller can be done in an instant. CLIP-Explorer is also essential for a later data investigation such as sequence motif detection or GO term analysis, since it reduces noise in the data. CLIP-Explorer enabled us to identify the involvement of RBFOX2 in splicing because of the availability of specific annotation tools like RCAS [41]. We could identify a decrease of RBFOX2 occupancy around the exon-intron boundaries, linked RBFOX2 to the regulation of splicing and DNA structural modifications and verified other observation from previous studies.

We recently have integrated GraphProt [52] into our pipeline, which is one of the popular tools for RBP binding profile predictions. We will also include StoatyDive [53] in a later version for a newly developed peak shape clustering for a better noise reduction, as it showed promising results for CLIP-Seq data. All in all, CLIP-Explorer is a flexible and easily extendable pipeline, which greatly simplifies CLIP-Seq data analysis on a transcriptome- and genome-wide scale.

Methods

CLIP-Explorer includes all major processes that are required to analyze CLIP-Seq data. All tools for each step were selected based on review [54, 19, 17] or benchmark articles (stated for each tool later in the paper), or experiences. The analysis involves three major steps as shown in Figure 1, each followed by a specific quality control. In the pre-processing step, the data is demultiplexed into the read libraries stemming from different experiments. If necessary, adapter sequences (using Cutadapt [33, 55]) as well as in-line barcodes and UMIs are removed. FastQC [34] performs during that step a standard quality check for the read and library quality.

The post-processing for CLIP experiments is similar to RNA-seq experiments, that is, the reads are aligned and filtered. An additional deduplication step is required in the case of recent CLIP-Seq protocols such as iCLIP and eCLIP. The deduplication removes PCR duplication artifacts. CLIP-Explorer performs another quality control during the post-processing using mainly FastQC [34], and deeptools [40].

The final and major part of CLIP-Explorer is the analysis step. CLIP-Explorer searches in that step for sequence and coverage motifs, and potential targets of the investigated RBP. Peak calling and motif detection are the fundamental and most critical processes. The quality and amount of detected binding sites can vary significantly based on the used tools, whereas the tools depend heavily on the experimental setup. Different tools therefore lead in part to different results. For that reason, CLIP-Explorer provides several peak calling and motif detection tools to fathom the robustness of the results.

Input and Output of CLIP-Explorer

The user needs to provide their experimental data in FASTA or FASTQ format. Nonstandard adapter sequences can be provided by the user. Otherwise, CLIP-Explorer automatically detects them. The pipeline is designed for multiplexed or demultiplexed paired read data and supports replicates and control experiments. Barcode sequences are required in case of demultiplexing. Other additional files are provided by the Galaxy database. CLIP-Explorer can be easily changed, for example, to allow for single end reads and different tool settings.

The user obtains a MultiQC [56] report for the raw, trimming, alignment, and deduplication quality to assess the quality of the raw data and important processing steps of CLIP-Explorer. MultiQC collects the FastQC reports made during the pre- and post-processing of CLIP-Explorer to inspect the mapping quality, elaborating on the amount of unmapped and multiply mapped reads, the length of mapped reads, and other important characteristics of the read library. Further quality control is provided by deeptools, as it can elicit differences in signal and control experiments. A heatmap and a fingerprint plot assess the correlation between the signal and control libraries providing evidence for a correct execution of the CLIP experiment. CLIP-Explorer also generates coverage files (bigWig and bedGraph) for the alignments and the crosslinking sites to inspect the peak calling quality. Most importantly, CLIP-Explorer will produce a bed and gtf file of significantly enriched regions, representing the binding sites of the protein on the transcriptome or genome. A MEME-ChIP [42] report will further analyze the peaks, detecting potential sequence motifs of the protein. A FIMO [42] report then lists reference sequences, which where not covered by the peak calling, but contain the detected sequence motifs. Finally, a RCAS (RNA centric annotation system) [41] report determines the target distribution of the protein over RNA classes and transcript regions. It also includes a GO term analysis and plots to fathom the coverage of the protein binding around splice junctions, along the transcripts and along various other regions. CLIP-Explorer can also generate a list of robust peaks (shared between all input files). This feature is useful for peakcallers that do not support replicated data such as Piranha.

Mapping and Deduplication

We integrated STAR [35] into CLIP-Explorer to map reads against the genome based on the good performance and usability of STAR for RNA-Seq data [57, 58, 59]. STAR is an annotation and splice aware aligner, which is important for transcriptomic data. Thus, we used extra information about the transcriptome. The data of RBFOX2 was mapped against hg19 to better reproduce the literature results, all other proteins such as DROSHA and HNRNPK were mapped against hg38. STAR was executed with the *two pass mode* turned on and in the end-to-end alignment scheme. CLIP-Explorer further checks for incomplete pairs, ambiguously mapped and low quality reads. CLIP-Explorer also includes a deduplication step to lower the false positive rate for the identification of binding regions. PCR duplicates are often collapsed into one representative [2, 19]. CLIP-Explorer identifies potential PCR duplicates with the help of UMI-tools [31]. Duplicated reads are identified after the alignment step, searching for reads with identical genomic positions (begin and end) and orientation. Yet, sequencing errors can also occur in the UMIs. UMI-tools clusters the reads based on their UMI to handle these sequencing errors. Thus, we merged sequences with a high node count and a small Hamming distance between unique UMIs [31].

Identification of Enriched Regions and Sequence Motifs

Searching for enriched regions and motifs is the most challenging task because of the differences in the gene expression between CLIP experiments and background controls. Hence, high false negative rates are a common result in the detection of differentially enriched regions (peaks) [19]. CLIP-Explorer allows to choose between three different peakcallers, namely PEAKachu, PureCLIP, and Piranha, which we picked based on their performance, availability (i.e., open source and conda package) and support of different CLIP-Seq data sets with replicates or controls [1, 20, 22, 60, 61, 62, 63].

PEAKachu was executed in adaptive mode with a mad multiplier of 0.0, a log2 fold change threshold of 2.0, an adjusted p-value (Benjamini-Hochberg procedure) threshold of 0.05 and a maximum insert size of 200, identified beforehand by Picard. All other parameters are set to their default values. We used for Piranha a negative binomial distribution with a bin size of 20 and a 0.05 p-value threshold. We did not include the control data for Piranha to test for possible experiments without control datasets. PureCLIP was trained on chromosome one, two, and three of hg38 (for RBFOX2 we used hg19, respectively), and executed with *-bc 0* as the default option. The resulting binding regions of PEAKachu, Piranha, and PureCLIP were then extended by 20 nucleotides because many peakcallers often call peaks that stop before the motif itself. The resulting regions were analyzed with RCAS [41] to determine the target distribution over genomic regions and possible binding patterns of RBFOX2. The peaks were also analyzed with the MEME Suite [42] tool package (MEME-ChIP) to find sequence motifs in the peaks. We used MEME-ChIP because of its versatility and performance for known motifs [64, 65]. Because of a missing ground truth for CLIP-Seq data, a benchmarking for motif finding tools is still missing. MEME-ChIP was set to find zero or one occurrence of the motif sites per sequence (zoops model).

Intersecting Peaks

We used the intersect module of bedtools [39] to assess the occurrence of the predicted peaks between CLIPper and CLIP-Explorer with PEAKachu, and between the three different CLIP-Explorer pipelines with Piranha, PureCLIP, and PEAKachu. We set bedtools intersect with the option *-s* to search for intersections on the same strand and kept the default value for *-f*, resulting in a minimum overlap of one base for overlapping regions to be reported. Further, we used the flag *-u* to consider only unique overlaps.

Analyzed Data

We used eCLIP data from DROSHA (ENCSR653HQC), HNRNPK (ENCSR828ZID), IGF2BP1 (ENCSR744GEU), KHDRBS1 (ENCSR628IDK), LIN28B (ENCSR861GYE), PTBP1 (ENCSR981WKN), QKI (ENCSR570WLM), RB-

FOX2 (ENCSR987FTF), SLBP (ENCSR483NOP), and U2AF2 (ENCSR202BFN) from the study by Van Nostrand et al. [13] to validate CLIP-Explorer. The data, which originated from human liver cancer cells (Hep G2) and immortalized myelogenous leukemia cells (K562), comprised two CLIP-Seq replicates and one control library for each RBP.

RBFOX2 was analyzed by CLIP-Explorer with PEAKachu, PureCLIP, and Piranha based on hg19. We took the peaks from the CLIPper pipeline (hg19 peaks ENCFF154DRN) and analyzed them with MEME-ChIP.

All other proteins were analyzed by CLIP-Explorer with PEAKachu on hg38. For the CLIPper pipeline we used the peaks from the IDR (signal normalization) from hg38 and analyzed them with MEME-ChIP. We investigated SLBP more thoroughly and used a more stringent parameter set for PEAKachu (i.e., no default) to find more robust peaks between replicates, since CLIPper does the same with an IDR calculation. We set the fold change to 4, the MAD to 1.0, the minimum block overlap to 0.5, minimum cluster expression fraction to 0.001, and the minimum block expression to 0.6. This parameter set was optimal for the SLBP data, but it can be sub-optimal for other proteins or CLIP-Seq data. To better compare the two tools, we also filtered for peaks that do not overlap with repeat regions and that have an annotated feature, because CLIPper (i.e., the CLIPper pipeline) implements similar filter steps.

We also analyzed data from an shRNA knockdown experiment against PTBP1 in Hep G2 cells followed by RNA-seq (ENCSR064DXG), including a control shRNA against no target (ENCSR603TCV). Both data samples are from ENCODE and encompass two replicates for both experiments. We took the alignments that were mapped with STAR against the genome (hg38). We calculated the coverage for each gene in hg38 with htseq-count [66]. We then obtained the log2 fold change for each gene from DESeq2 [43], taking both the knockdown and the control experiment into account. We then intersected the identified sites from PEAKachu and CLIPper for PTBP1 with the genes of hg38 and plotted the CDF of the log2 fold changes.

Availability of Supporting Source Code and Requirements

Project name: CLIP-Explorer
Project home page: https://clipseq.usegalaxy.eu/
Operating system(s): Galaxy
Training material: https://galaxyproject.github.io/training-material/topics/transcriptomics/tutorials/clipseq/tutorial.html
biotools:CLIP-Explorer
SciCrunch.org: SCR_018128

Availability of Supporting Data and Materials

CLIP-Explorer provides a small dataset for a test run, which can be found in the training material and on the CLIP-Explorer website. The whole eCLIP data used in this paper, such as RBFOX2 or PTBP1, is listed in the supplementary of the study by Van Nostrand et al. [13].

Additional files

Supplementary Table 1. Top five DREME sequence motifs of MEME-ChIP [42] of Different Proteins. CLIP-Explorer's sequence logos of different proteins from the binding regions that were identified by PEAKachu. Furthermore, sequence motifs of MEME-ChIP from the binding regions that were identified by the CLIPper algorithm [21]. The sequence motifs of CLIP-Explorer and CLIPper originated from eCLIP data [13]. To compare the sequence logos other motifs were collected from different databases [48, 49].

Supplementary Table 2. Peak intersections between PEAKachu [20] of CLIP-Explorer and CLIPper from the the study by Van Nostrand et al. [13]. A list of Venn diagrams showing the overlap between PEAKachu [20] (CLIP-Explorer) and CLIPper peaks [13].

Supplementary Table 3. Comparison of the peak length and the distance between the peaks between PEAKachu, Piranha, PureCLIP, and CLIPper for the RBFOX2 data.

Supplementary Table 4. Number of peaks of the SLBP peak set of PEAKachu and CLIPper overlapping with the stated features (P-genes = Pseudogenes).

Declarations

List of abbreviations

CLIP-Seq: Crosslinking immunoprecipitation in combination with high-throughput sequencing; CDF: Cumulative density function; IDR: irreducible discovery rate; RBP: RNA-binding proteins.

Ethical Approval

Not applicable

Consent for Publication

Not applicable

Competing Interests

The authors declare that they have no competing interests.

Funding

This study was funded by the Deutsche Forschungsgemeinschaft (DFG, German Research Foundation) grant 322977937/GRK2344 2017 MeInBio – BioInMe Research Training Group, Germany's Excellence Strategy (CIBSS – EXC-2189 – Project ID 390939984), DFG grant BA2168/11-2 SPP 1738, DFG grant TRR 167/1 2027 NeuroMac, and by the Collaborative Research Centre 992 Medical Epigenetics (DFG grant SFB 992/2 2016).

Author's Contributions

F.H. and D.M. performed the computational analysis. R.B., and D.M. initialized the project, and supervised the research. F.H. and R.B. wrote the manuscript with inputs from other authors. M.U. integrated Graphprot, PureCLIP and RCAS into CLIP-Explorer. All authors read and approved the final manuscript.

Acknowledgements

We are grateful to the members of the Galaxy team Freiburg (http://www.bioinf.uni-freiburg.de/Galaxy/) and to Torsten Houwaart for his support.

References

1. Uren PJ, Bahrami-Samani E, Burns SC, Qiao M, Karginov FV, Hodges E, et al. Site identification in high-throughput RNA-protein interaction data. Bioinformatics 2012;28(23):3013–3020.
2. Chakrabarti AM, Haberman N, Praznik A, Luscombe NM, Ule J. Data Science Issues in Understanding Protein-RNA Interactions. bioRxiv 2017;.
3. Hentze MW, Castello A, Schwarzl T, Preiss T. A brave new world of RNA-binding proteins. Nature Reviews Molecular Cell Biology 2018;19:327–341.
4. Baltz AG, Munschauer M, Schwanhäusser B, Vasile A, Murakawa Y, Schueler M, et al. The mRNA-bound proteome and its global occupancy profile on protein-coding transcripts. Molecular cell 2012;46(5):674–690.
5. Castello A, Fischer B, Eichelbaum K, Horos R, Beckmann BM, Strein C, et al. Insights into RNA Biology from an Atlas of Mammalian mRNA-Binding Proteins. Cell 2012;149(6):1393–1406.
6. Gerstberger S, Hafner M, Tuschl T. A census of human RNA-binding proteins. Nature Reviews Genetics 2014;15(12):829–845.
7. Beckmann BM, Castello A, Medenbach J. The expanding universe of ribonucleoproteins: of novel RNA-binding proteins and unconventional interactions. Pflügers Archiv-European Journal of Physiology 2016;468(6):1029–1040.
8. Pereira B, Billaud M, Almeida R. RNA-Binding Proteins in Cancer: Old Players and New Actors. Trends in cancer 2017;3(7):506–528.
9. Nussbacher JK, Batra R, Lagier-Tourenne C, Yeo GW. RNA-binding proteins in neurodegeneration: Seq and you shall receive. Trends in neurosciences 2015;38(4):226–236.
10. Jankowsky E, Harris ME. Specificity and nonspecificity in RNA-protein interactions. Nature reviews Molecular cell biology 2015;16(9):533–544.
11. Hafner M, Landthaler M, Burger L, Khorshid M, Hausser J, Berninger P, et al. Transcriptome-wide Identification of RNA-Binding Protein and MicroRNA Target Sites by PAR-CLIP. Cell 2010;141(1):129–141.
12. Huppertz I, Attig J, D'Ambrogio A, Easton LE, Sibley CR, Sugimoto Y, et al. iCLIP: Protein-RNA interactions at nucleotide resolution. Methods 2014;65(3):274–287.
13. Van Nostrand EL, Pratt GA, Shishkin AA, Gelboin-Burkhart C, Fang MY, Sundararaman B, et al. Robust transcriptome-wide discovery of RNA-binding protein binding sites with enhanced CLIP (eCLIP). Nature methods 2016;13(6):508.
14. Lee FC, Ule J. Advances in CLIP technologies for studies of protein-RNA interactions. Molecular cell 2018;69(3):354–369.
15. Sugimoto Y, König J, Hussain S, Zupan B, Curk T, Frye M, et al. Analysis of CLIP and iCLIP methods for nucleotide-resolution studies of protein-RNA interactions. Genome biology 2012;13(8):R67.
16. König J, Zarnack K, Rot G, Curk T, Kayikci M, Zupan B, et al. iCLIP reveals the function of hnRNP particles in splicing at individual nucleotide resolution. Nature structural & molecular biology 2010;17(7):909–915.
17. Wheeler EC, Van Nostrand EL, Yeo GW. Advances and challenges in the detection of transcriptome-wide protein-RNA interactions. Wiley Interdisciplinary Reviews: RNA 2018;9(1):e1436.
18. Maticzka D, Ilik IA, Aktas T, Backofen R, Akhtar A. uvCLAP is a fast and non-radioactive method to identify in vivo targets of RNA-binding proteins. Nature communications 2018;9(1):1142.
19. Uhl M, Houwaart T, Corrado G, Wright PR, Backofen R. Computational analysis of CLIP-seq data. Methods 2017;118:60–72.
20. Bischler T, Maticzka D, Förstner KU, Wright PR, PEAKachu;. https://github.com/tbischler/PEAKachu.
21. Lovci MT, Ghanem D, Marr H, Arnold J, Gee S, Parra M, et al. Rbfox proteins regulate alternative mRNA splicing through evolutionarily conserved RNA bridges. Nature structural & molecular biology 2013;20:1434.
22. Krakau S, Richard H, Marsico A. PureCLIP: capturing target-specific protein-RNA interaction footprints from single-nucleotide CLIP-seq data. Genome biology 2017;18(1):240.
23. Chen B, Yun J, Kim MS, Mendell JT, Xie Y. PIPE-CLIP: a comprehensive online tool for CLIP-seq data analysis. Genome biology 2014;15(1):R18.
24. Maragkakis M, Alexiou P, Nakaya T, Mourelatos Z. CLIPSeqTools—a novel bioinformatics CLIP-seq analysis suite. RNA 2016;22(1):1–9.
25. Curk T, Rot G, Gorup u, Zmrzlikar J, Konig J, Sugimoto Y, et al. iCount: protein-RNA interaction iCLIP data analysis 2016;.
26. Afgan E, Baker D, Batut B, van den Beek M, Bouvier D, Čech M, et al. The Galaxy platform for accessible, reproducible and collaborative biomedical analyses: 2018 update. Nucleic acids research 2018;46(W1):W537–W544.
27. Gehman LT, Meera P, Stoilov P, Shiue L, O'Brien JE, Meisler MH, et al. The splicing regulator Rbfox2 is required for both cerebellar development and mature motor function. Genes & development 2012;.
28. Yeo GW, Coufal NG, Liang TY, Peng GE, Fu XD, Gage FH. An RNA code for the FOX2 splicing regulator revealed by mapping RNA-protein interactions in stem cells. Nature Structural and Molecular Biology 2009;16(2):130.
29. Singh RK, Xia Z, Bland CS, Kalsotra A, Scavuzzo MA, Curk T, et al. Rbfox2-Coordinated Alternative Splicing of Mef2d and Rock2 Controls Myoblast Fusion during Myogenesis. Molecular cell 2014;55(4):592–603.
30. Girardot C, Scholtalbers J, Sauer S, Su SY, Furlong EE. Je, a versatile suite to handle multiplexed NGS libraries with unique molecular identifiers. BMC bioinformatics 2016;17(1):419.
31. Smith TS, Heger A, Sudbery I. UMI-tools: Modelling sequencing errors in Unique Molecular Identifiers to improve quantification accuracy. Genome research 2017;.
32. Maticzka D, bctools;. https://github.com/dmaticzka/bctools.
33. Martin M. Cutadapt removes adapter sequences from high-throughput sequencing reads. EMBnet journal 2011;17(1):pp–10.
34. Andrews S, et al. FastQC: a quality control tool for high throughput sequence data 2010;.
35. Dobin A, Davis CA, Schlesinger F, Drenkow J, Zaleski C, Jha S, et al. STAR: ultrafast universal RNA-seq aligner. Bioinformatics 2013;29(1):15–21.
36. Langmead B, Salzberg SL. Fast gapped-read alignment with Bowtie 2. Nature methods 2012;9(4):357.
37. Hoffmann S, Otto C, Kurtz S, Sharma CM, Khaitovich P, Vogel J, et al. Fast mapping of short sequences with mismatches, insertions and deletions using index structures. PLoS computational biology 2009;5(9):e1000502.
38. Wysoker A, Fennell T, Ruan J, Homer N, Marth G, Abecasis G, et al. The Sequence alignment/map (SAM) format and SAMtools. Bioinformatics 2009;25:2078–2079.
39. Quinlan AR, Hall IM. BEDTools: a flexible suite of utilities for comparing genomic features. Bioinformatics 2010;26(6):841–842.
40. Ramírez F, Ryan DP, Grüning B, Bhardwaj V, Kilpert F, Richter AS, et al. deepTools2: a next generation web server

for deep-sequencing data analysis. Nucleic acids research 2016;44(W1):W160–W165.
41. Uyar B, Yusuf D, Wurmus R, Rajewsky N, Ohler U, Akalin A. RCAS: an RNA centric annotation system for transcriptome-wide regions of interest. Nucleic acids research 2017;45(10):e91–e91.
42. Bailey TL, Boden M, Buske FA, Frith M, Grant CE, Clementi L, et al. MEME SUITE: tools for motif discovery and searching. Nucleic acids research 2009;37(suppl_2):W202–W208.
43. Love MI, Huber W, Anders S. Moderated estimation of fold change and dispersion for RNA-seq data with DESeq2. Genome biology 2014;15(12):550.
44. broadinstitute, Picard;. http://broadinstitute.github.io/picard.
45. Maticzka D, targetdist; 2016. https://github.com/dmaticzka/targetdist.git.
46. Guilmatre A, Huguet G, Delorme R, Bourgeron T. The emerging role of SHANK genes in neuropsychiatric disorders. Developmental neurobiology 2014;74(2):113–122.
47. Leblond CS, Heinrich J, Delorme R, Proepper C, Betancur C, Huguet G, et al. Genetic and functional analyses of SHANK2 mutations suggest a multiple hit model of autism spectrum disorders. PLoS genetics 2012;8(2):e1002521.
48. Giudice G, Sánchez-Cabo F, Torroja C, Lara-Pezzi E. ATtRACT – a database of RNA-binding proteins and associated motifs. Database 2016;2016.
49. Ray D, Kazan H, Cook KB, Weirauch MT, Najafabadi HS, Li X, et al. A compendium of RNA-binding motifs for decoding gene regulation. Nature 2013;499(7457):172.
50. Teplova M, Hafner M, Teplov D, Essig K, Tuschl T, Patel DJ. Structure–function studies of STAR family Quaking proteins bound to their in vivo RNA target sites. Genes & development 2013;27(8):928–940.
51. Wilbert ML, Huelga SC, Kapeli K, Stark TJ, Liang TY, Chen SX, et al. LIN28 binds messenger RNAs at GGAGA motifs and regulates splicing factor abundance. Molecular cell 2012;48(2):195–206.
52. Maticzka D, Lange SJ, Costa F, Backofen R. GraphProt: modeling binding preferences of RNA-binding proteins. Genome biology 2014;15(1):R17.
53. Heyl F, Backofen R. StoatyDive: Evaluation and Classification of Peak Profiles for Sequencing Data. bioRxiv 2019;p. 799114.
54. Bottini S, Pratella D, Grandjean V, Repetto E, Trabucchi M. Recent computational developments on CLIP-seq data analysis and microRNA targeting implications. Briefings in bioinformatics 2018;19(6):1290–1301.
55. Davis MP, van Dongen S, Abreu-Goodger C, Bartonicek N, Enright AJ. Kraken: a set of tools for quality control and analysis of high-throughput sequence data. Methods 2013;63(1):41–49.
56. Ewels P, Magnusson M, Lundin S, Käller M. MultiQC: summarize analysis results for multiple tools and samples in a single report. Bioinformatics 2016;32(19):3047–3048.
57. Baruzzo G, Hayer KE, Kim EJ, Di Camillo B, FitzGerald GA, Grant GR. Simulation-based comprehensive benchmarking of RNA-seq aligners. Nature methods 2017;14(2):135.
58. Engström PG, Steijger T, Sipos B, Grant GR, Kahles A, Alioto T, et al. Systematic evaluation of spliced alignment programs for RNA-seq data. Nature methods 2013;10(12):1185.
59. Williams CR, Baccarella A, Parrish JZ, Kim CC. Empirical assessment of analysis workflows for differential expression analysis of human samples using RNA-Seq. BMC bioinformatics 2017;18(1):38.
60. Bottini S, Hamouda-Tekaya N, Tanasa B, Zaragosi LE, Grandjean V, Repetto E, et al. From benchmarking HITS-CLIP peak detection programs to a new method for identification of miRNA-binding sites from Ago2-CLIP data. Nucleic acids research 2017;45(9):e71–e71.
61. Park S, Ahn SH, Cho ES, Cho YK, Jang ES, Chi SW. CLIPick: a sensitive peak caller for expression-based deconvolution of HITS-CLIP signals. Nucleic acids research 2018;46(21):11153–11168.
62. Drewe-Boss P, Wessels HH, Ohler U. omniCLIP: probabilistic identification of protein-RNA interactions from CLIP-seq data. Genome biology 2018;19(1):183.
63. Corcoran DL, Georgiev S, Mukherjee N, Gottwein E, Skalsky RL, Keene JD, et al. PARalyzer: definition of RNA binding sites from PAR-CLIP short-read sequence data. Genome biology 2011;12(8):R79.
64. Lihu A, Holban Ş. A review of ensemble methods for de novo motif discovery in ChIP-Seq data. Briefings in bioinformatics 2015;16(6):964–973.
65. Hashim FA, Mabrouk MS, Al-Atabany W. Review of Different Sequence Motif Finding Algorithms. Avicenna journal of medical biotechnology 2019;11(2):130.
66. Anders S, Pyl PT, Huber W. HTSeq—a Python framework to work with high-throughput sequencing data. Bioinformatics 2015;31(2):166–169.

Supplementary 1

Click here to access/download

Supplementary Material

Supplements_1.pdf

Supplementary 2

Click here to access/download
Supplementary Material
Supplements_2.pdf

Supplementary 3

Click here to access/download
Supplementary Material
Supplements_3.pdf

Supplementary 4

Click here to access/download
Supplementary Material
Supplements_4.pdf

bib style

Click here to access/download
Supplementary Material
vancouver-authoryear.bst

style

Click here to access/download
Supplementary Material
oup-contemporary.cls

bib

Click here to access/download
Supplementary Material
paper-refs.bib

Click here to access/download
Supplementary Material
main.tex
