# Supplementary material for: Galaxy CLIP-Explorer: a web server for CLIP-Seq data analysis
Source: Gigascience. 2020 Nov 11;9(11):giaa108. doi: 10.1093/gigascience/giaa108 (PMC7657819; doi:10.1093/gigascience/giaa108)
Supplement: giaa108_Supplemental_Files [file giaa108_supplemental_files.zip › Supplements_1.pdf]

| Protein | Source | Motif 1 & E-value | Motif 2 & E-value | Motif 3 & E-value | Motif 4 & E-value | Motif 5 & E-value |
|---|---|---|---|---|---|---|
| DROSHA | CLIP-Explorer | 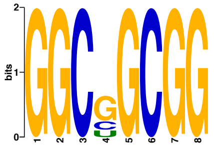 5.3e-016 | 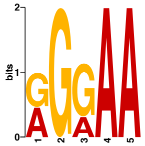 6.7e-011 | 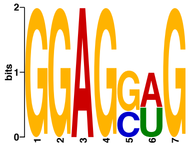 6.0e-011 | 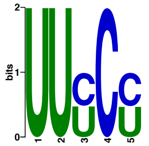 7.6e-009 | 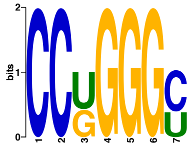 3.9e-007 |
| | CLIPper | 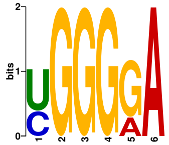 4.8e-013 | 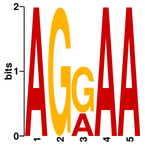 9.1e-009 | 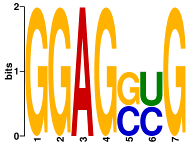 1.4e-007 | 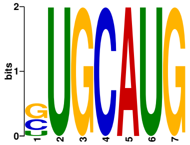 6.4e-008 | 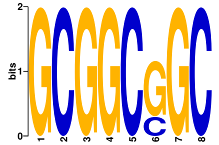 1.6e-006 |
| HNRNPK | Database | 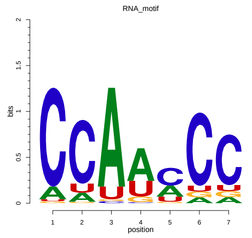 | 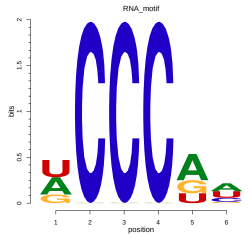 | 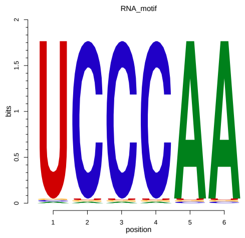 | 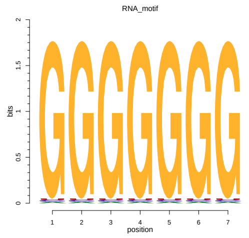 | |
| | CLIP-Explorer | 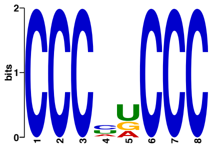 3.9e-136 | 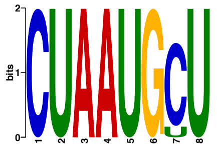 6.4e-063 | 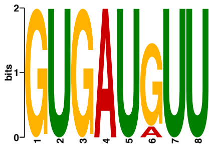 9.9e-060 | 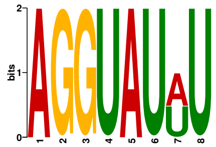 5.3e-035 | 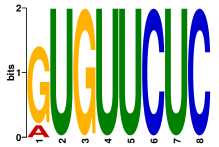 9.6e-034 |
| | CLIPper | 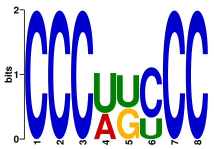 4.5e-066 | 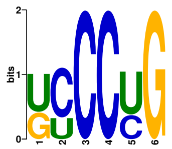 5.1e-027 | 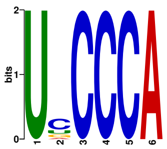 1.1e-019 | 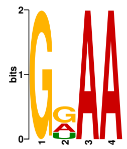 3.0e-008 | 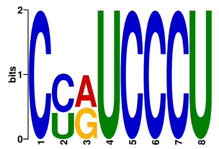 1.3e-006 |

| | | | | | | |
|---|---|---|---|---|---|---|
| IGF2BP1 | Database | 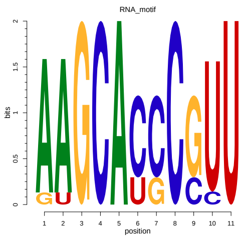 | | | | |
| | CLIP-Explorer | 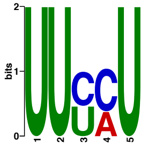 4.9e-039 | 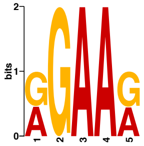 2.2e-037 | 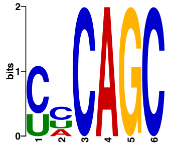 2.9e-020 | 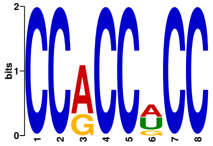 1.2e-019 | 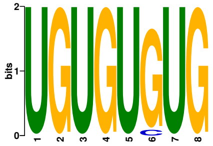 1.1e-016 |
| | CLIPper | 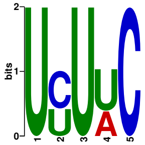 3.2e-011 | 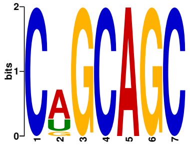 3.0e-009 | 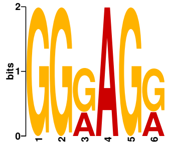 8.8e-007 | 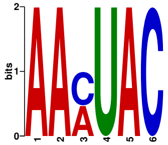 1.5e-005 | 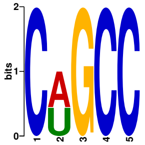 4.4e-005 |
| KHDRBS1 | Database | 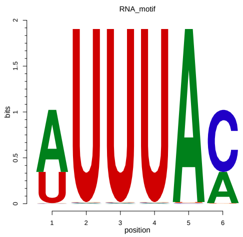 | 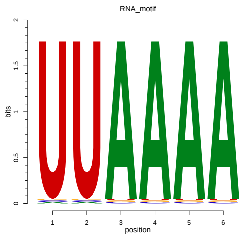 | 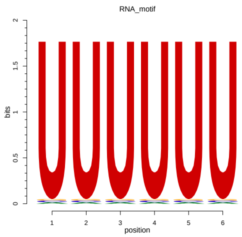 | 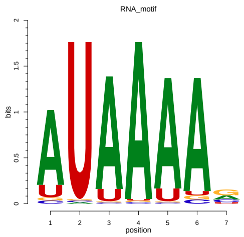 | |
| | CLIP-Explorer | 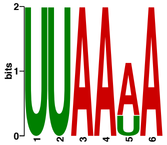 1.6e-193 | 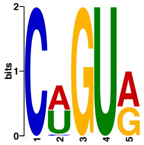 2.0e-083 | 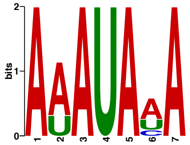 4.4e-062 | 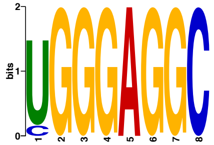 1.8e-059 | 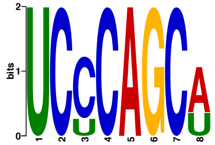 4.1e-063 |

| | | | | | | |
|---|---|---|---|---|---|---|
| | CLIPper | 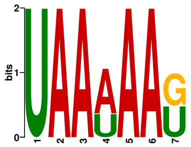 3.1e-025 | 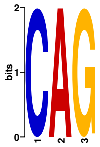 4.8e-006 | 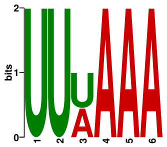 7.9e-006 | 2.2e-002 | 3.4e-002 |
| LIN28B | CLIP-Explorer | 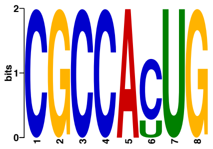 8.9e-038 | 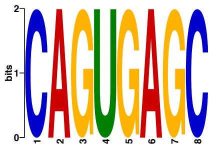 1.7e-037 | 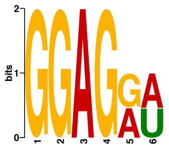 3.6e-033 | 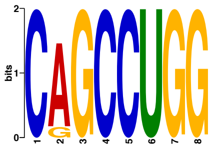 7.0e-029 | 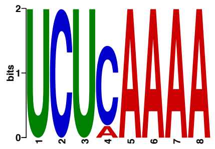 1.7e-023 |
| | CLIPper | 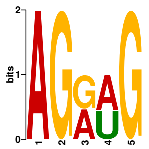 3.5e-019 | 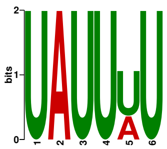 7.0e-015 | 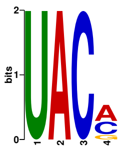 2.1e-012 | 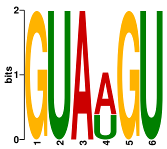 4.1e-010 | 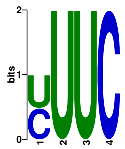 1.1e-008 |
| PTBP1 | Database | 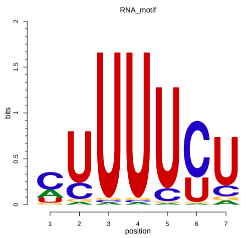 | 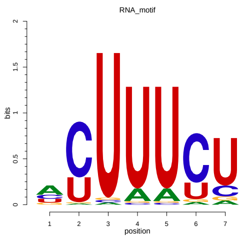 | 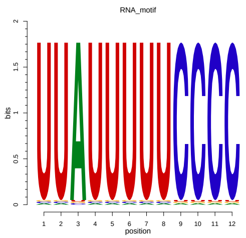 | 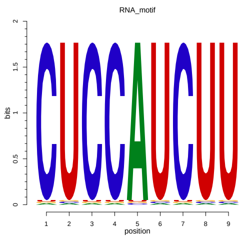 | 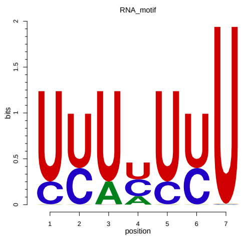 |
| | CLIP-Explorer | 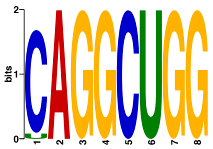 2.5e-153 | 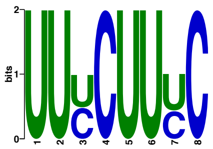 4.4e-136 | 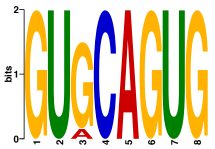 1.0e-103 | 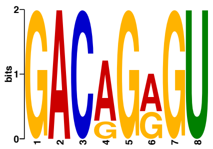 2.7e-095 | 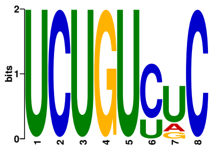 2.1e-076 |

| | | | | | | |
|---|---|---|---|---|---|---|
| | CLIPper | 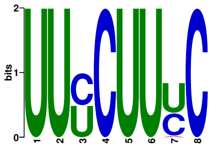 1.7e-107 | 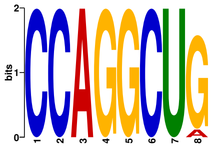 2.9e-089 | 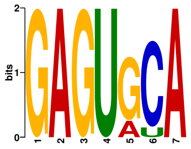 1.8e-066 | 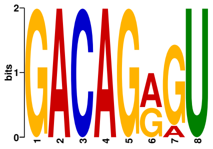 1.0e-042 | 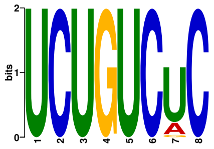 2.9e-041 |
| QKI | Database | 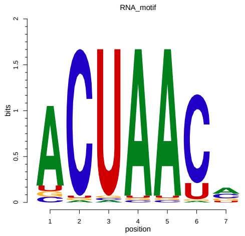 | 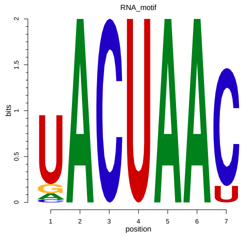 | 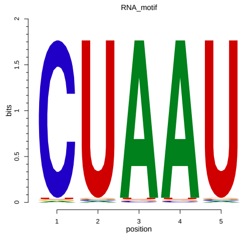 | 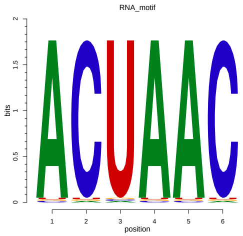 | 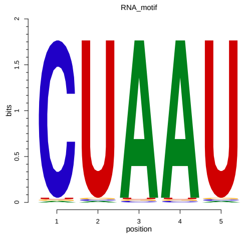 |
| | CLIP-Explorer | 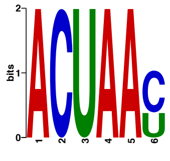 5.6e-630 | 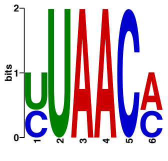 4.1e-084 | 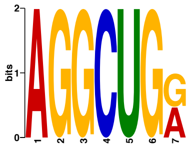 3.6e-070 | 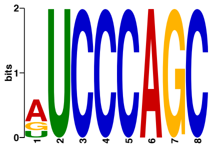 4.9e-057 | 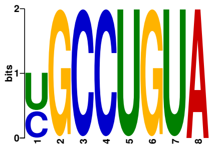 1.5e-037 |
| | CLIPper | 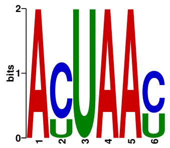 1.3e-1171 | 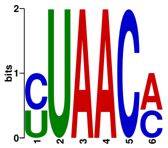 1.0e-085 | 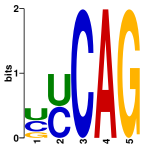 4.0e-038 | 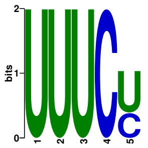 9.4e-031 | 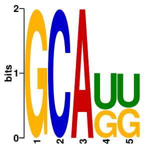 5.5e-018 |
| RBFOX2 | Database | 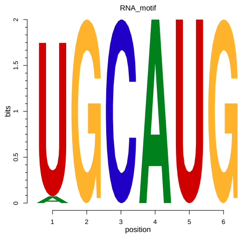 | 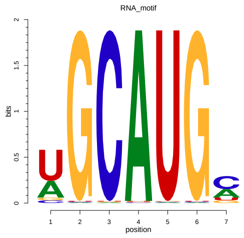 | | | |

| | | | | | | |
|---|---|---|---|---|---|---|
| | CLIP-Explorer | 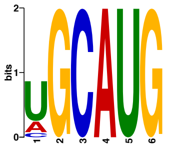 6.1e-702 | 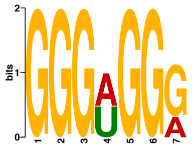 3.2e-075 | 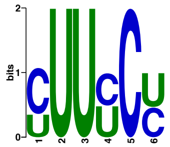 2.7e-049 | 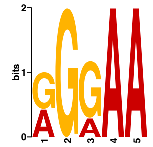 6.2e-045 | 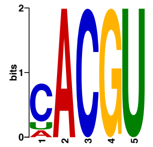 1.3e-027 |
| | CLIPper | 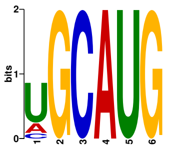 2.1e-504 | 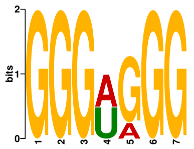 6.0e-032 | 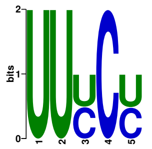 4.7e-019 | 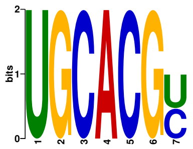 4.1e-018 | 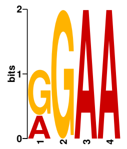 5.4e-015 |
| SLBP | CLIP-Explorer | 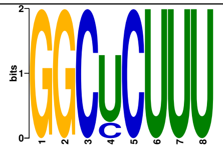 2.8e-015 | 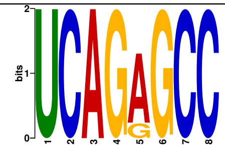 1.4e-010 | 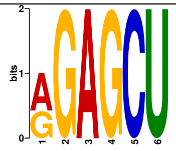 6.3e-004 | 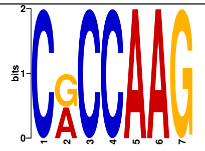 1.2e-003 | 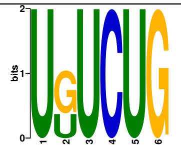 1.2e-003 |
| | CLIPper | 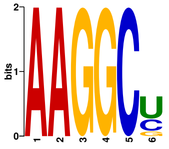 3.7e-014 | 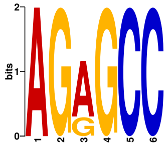 2.2e-009 | 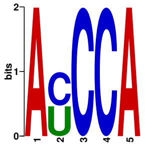 1.1e-003 | 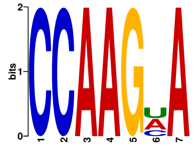 6.8e-003 | 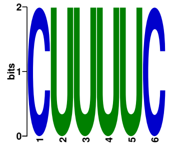 9.6e-003 |
| U2AF2 | Database | 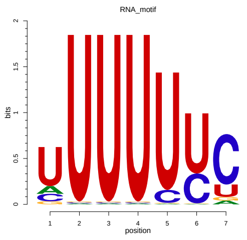 | 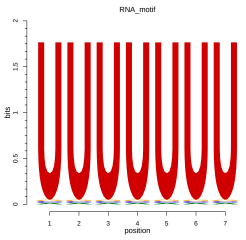 | | | |
| | CLIP-Explorer | 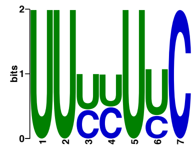 | 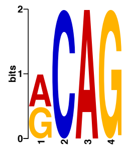 | 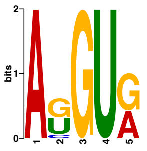 | 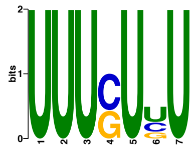 | 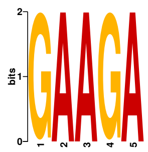 |

| | | | | | | |
|---|---|---|---|---|---|---|
| | CLIPper | 1.8e-126 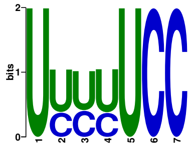 1.2e-229 | 1.3e-076 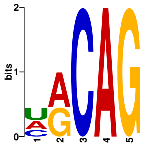 2.4e-126 | 1.7e-050 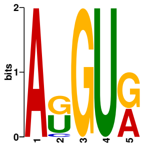 7.9e-075 | 1.8e-043 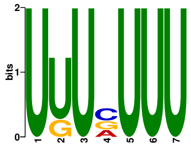 1.3e-055 | 1.6e-040 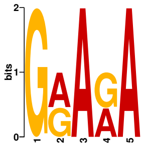 8.8e-040 |
