# Supplementary material for: Galaxy CLIP-Explorer: a web server for CLIP-Seq data analysis
Source: Gigascience. 2020 Nov 11;9(11):giaa108. doi: 10.1093/gigascience/giaa108 (PMC7657819; doi:10.1093/gigascience/giaa108)
Supplement: giaa108_Supplemental_Files [file giaa108_supplemental_files.zip › Supplements_4.pdf]

Feature	PEAKachu	CLIPper
Total	220	161
Repeats	0	0
Intron Repeats	0	0
ncRNA P-genes	1	0
rRNA P-genes	0	0
tRNA P-genes	0	0
Histones	163	135
ncRNA	48	17
