# Supplementary figures and images for: Galaxy CLIP-Explorer: a web server for CLIP-Seq data analysis
Source: Gigascience. 2020 Nov 11;9(11):giaa108. doi: 10.1093/gigascience/giaa108 (PMC7657819; doi:10.1093/gigascience/giaa108)

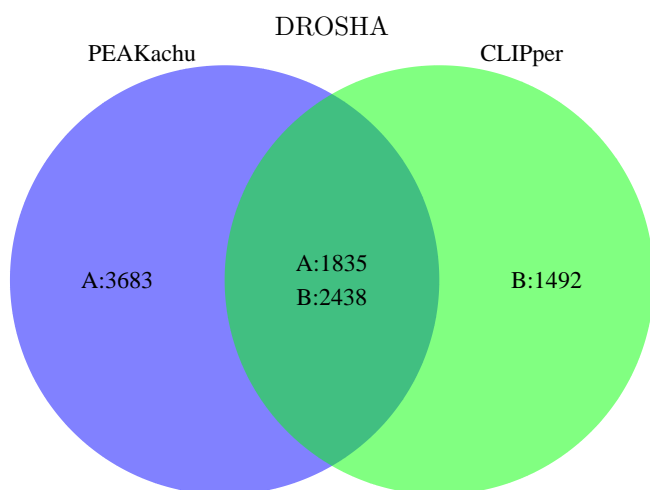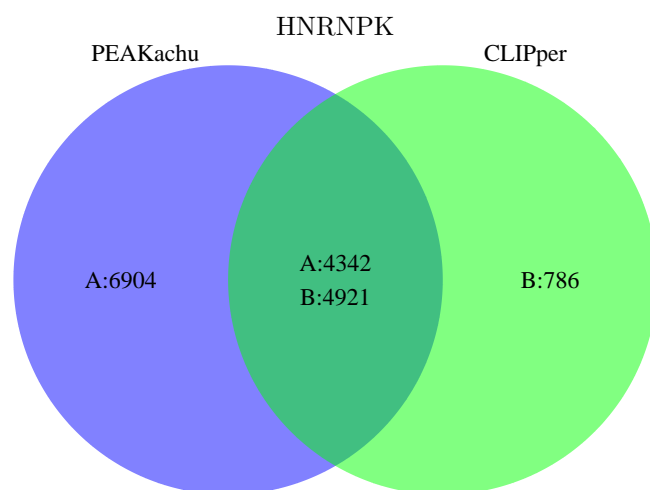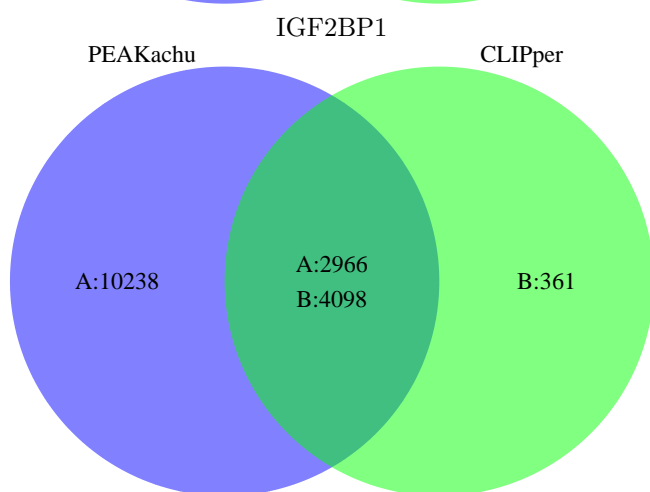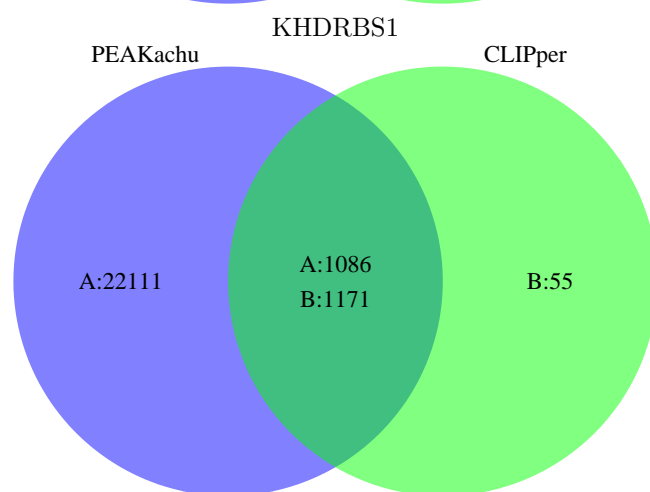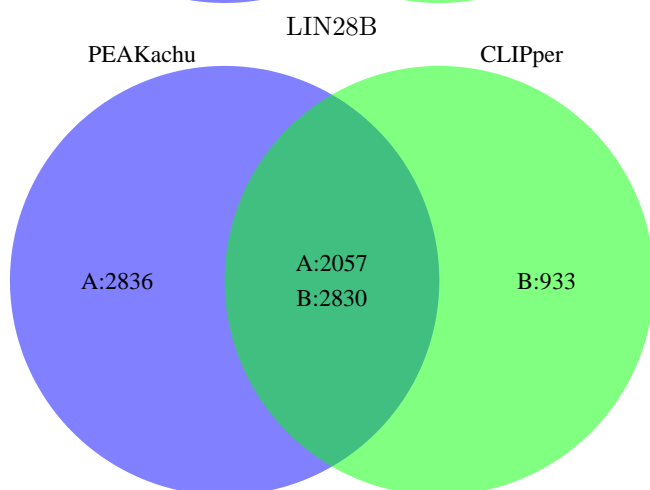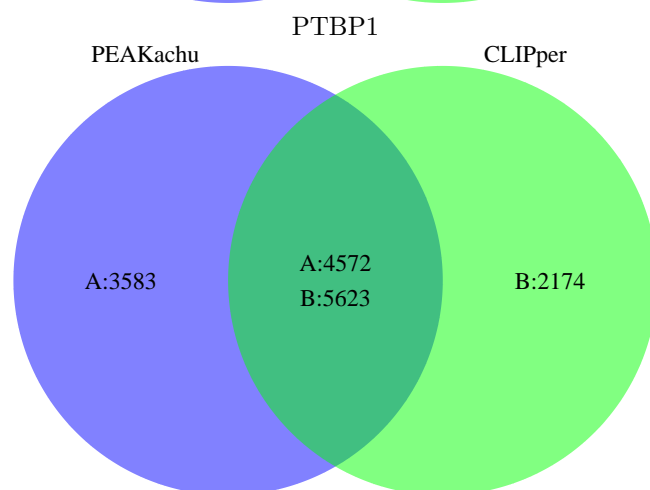

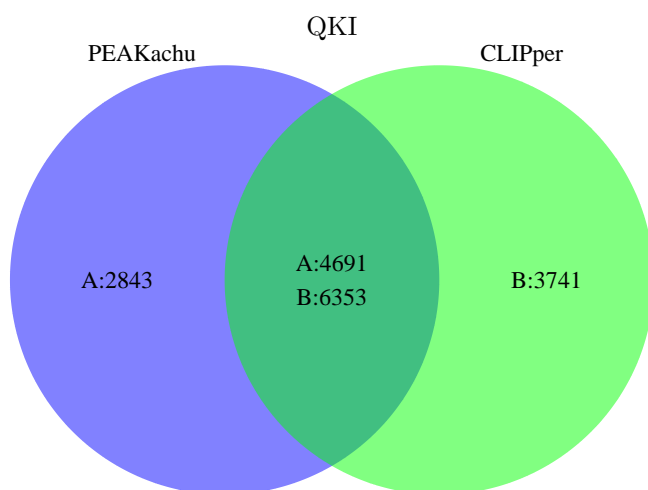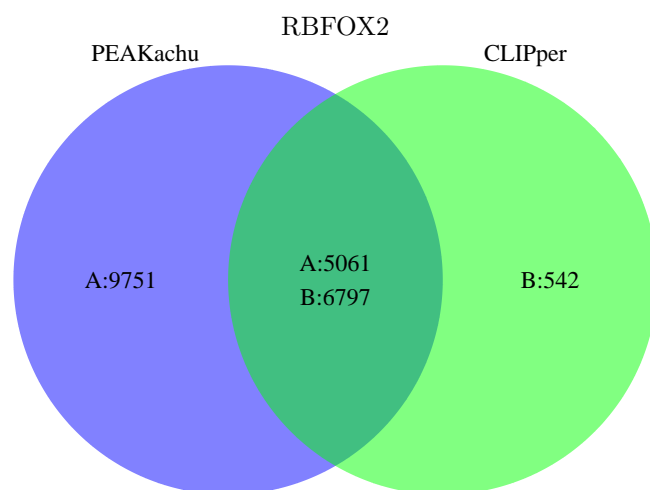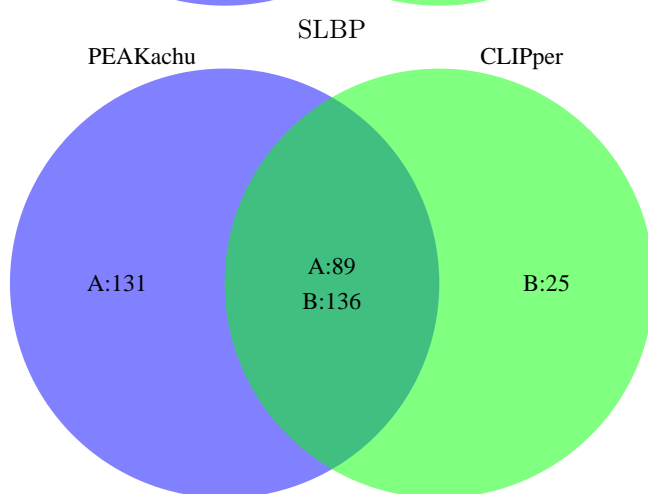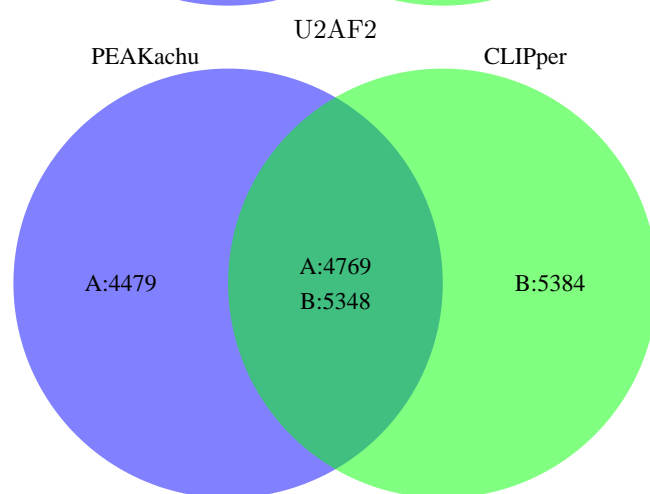

Supplement: giaa108_Supplemental_Files [file giaa108_supplemental_files.zip › Supplements_2.pdf]

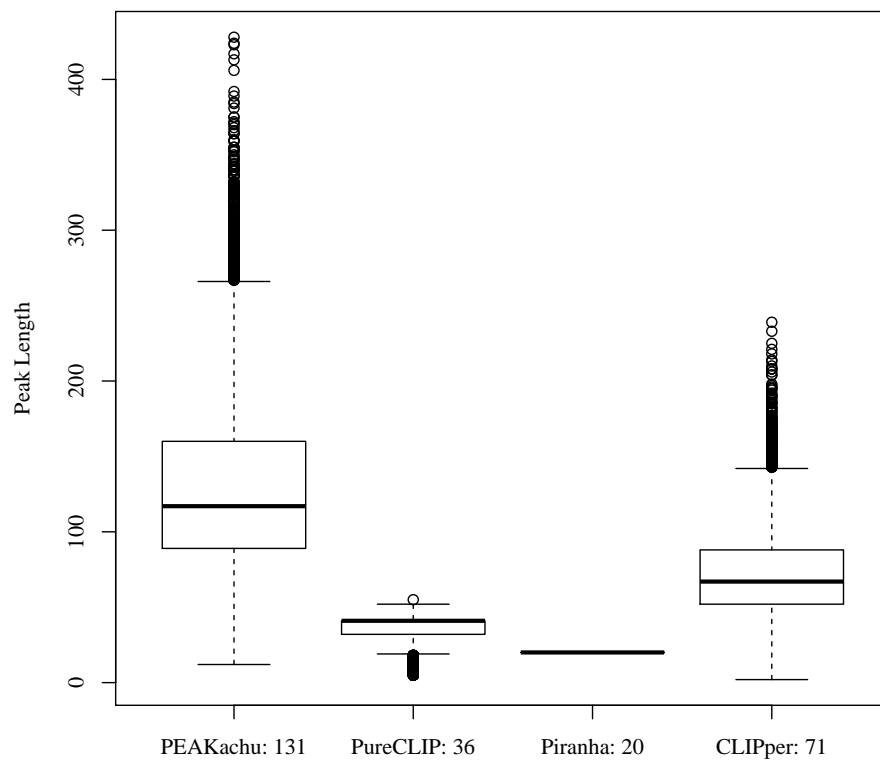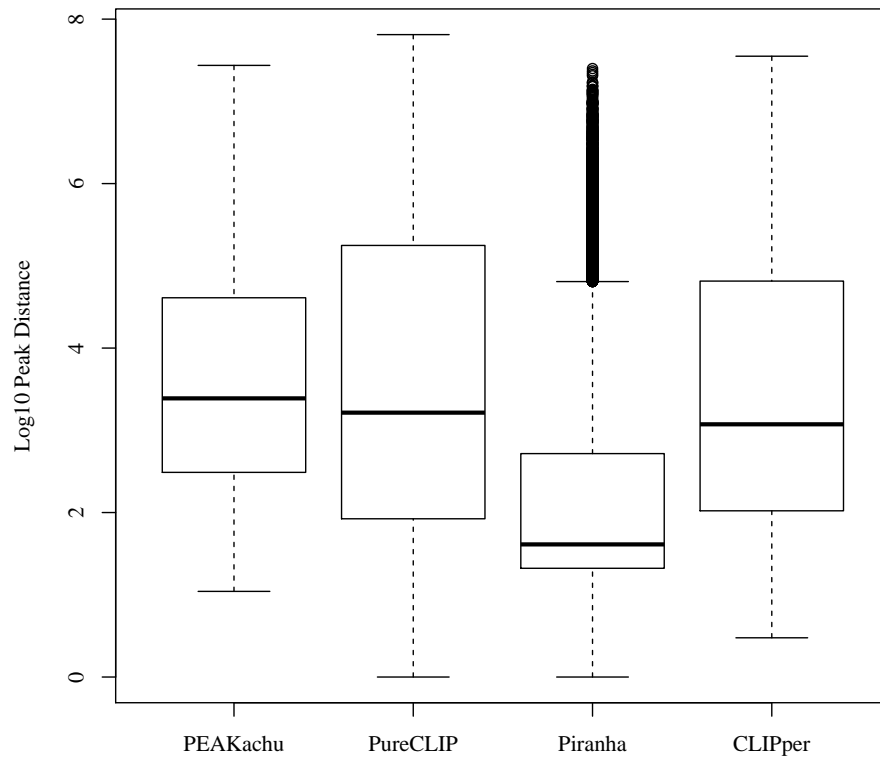

Supplement: giaa108_Supplemental_Files [file giaa108_supplemental_files.zip › Supplements_3.pdf]
