# Supplementary material for: Galaxy CLIP-Explorer: a web server for CLIP-Seq data analysis
Source: Gigascience. 2020 Nov 11;9(11):giaa108. doi: 10.1093/gigascience/giaa108 (PMC7657819; doi:10.1093/gigascience/giaa108)
Supplement: giaa108_Response_to_Reviewer_Comments_Original_Submission [file giaa108_response_to_reviewer_comments_original_submission.pdf]

Author's Response To Reviewer Comments

Close

\section{Reviewer \#1}

The authors proposed a new web server for CLIP-seq data analysis. Although the work is sound and interesting, I have few suggestions/concerns that might improve the work.

Major revisions:
The authors propose specific tools for each step of the pipeline, however they missed to explain why they made this choice. The authors missed to cite recent CLIP-seq tools benchmark and to put their work in relationship with these previous related papers.

\begin{answer}
The use of Cutadapt for adapter trimming is nearly a standard for NGS data. Trim Galore is another tool, but it is also using Cutadapt in the background, and the adapter trimming cannot be turned off, which might be useful if the user has data where the adapters were already removed. Other tools like Trimmomatic are also supported by Galaxy and can be used instead of Cutadapt. We assume that a comprehensive adapter trimming method benchmark has not been done yet since the only real difference between them is the runtime, because adapter trimming is a step which can be easily checked. However, we put a short statement into the method section, where we cite a paper comparing the runtime of different adapter trimming methods, where Cutadapt outperformed the rest.

The use of UMI-tools is based on the fact that it is the only tool that can select UMIs taking possible sequencing errors into account, which might occur in the UMIs.

We put a statement into the methods section (Mapping and Deduplication) for the mapping with STAR. We integrated STAR into CLIP-Explorer because of the good performance and usability of STAR for RNA-Seq data. STAR is an annotation and splice aware aligner, which is important for transcriptomics data. We cited three benchmark papers, where STAR was compared with other read mappers for RNA-Seq data.

The use of MEME-ChIP for sequence motif finding results from its versatility and performance for known motifs. It is a combinatorial approach of an enumeration method (DREME) and a probabilistic model (MEME). Furthermore, it encompasses a module for local enrichments in the sequence motifs (CentriMo) and a module for the detection of secondary sequence motifs (SpaMo). Because of a missing ground truth (missing experimental methods to verify predicted motifs) for CLIP-Seq data, a benchmarking for motif finding tools is still missing. We put a statement into the methods section (Identification of Enriched Regions and Sequence Motifs).

The use of RCAS is also based on the versatility of the tool and the performance with RBFOX2 and other proteins. The tool simply collects the information obtained from the peak regions and runs a broad analysis of certain features that can then be used to imply biological functions. It is the only tool so far that can do such an extensive analysis for RNA-Seq related data.

The rest of quality control tools are either standard in any NGS data analysis (e.g., FastQC) or are short scripts to collect important features of CLIP-Seq experiments (e.g, bctools).

We also state now in the method section a selection of review articles for the analysis of CLIP-Seq data.
\end{answer}

Furthermore the benchmark of peak calling tools should be extended, including exact metrics to evaluate the comparisons of the performances of the selected tools.

\begin{answer}
We do think that a statement that a peakcaller is better or worse is not appropriate because of a missing

ground truth. We show and included now more metrics for the reader and potential user to give an idea about the characteristics of the different peakcallers, thus the user can make a sophisticated choice for their type of data. For that reason, we put a new Table and new results into the supplements of the paper (Supplementary Table 3 and 4).

The new Table compares all four peakcallers (PEAKachu, Piranha, PureCLIP, and CLIPper) regarding potential CLIP-Seq artifacts and biases for the RBFOX2 example data of the paper. The table shows the percentage of peaks that have the stated feature. The table describes and tries to answer several questions. (1) How many peaks do overlap with other peaks of the same peakcaller, i.e., how many peaks deliver unique information? (2,3) How many peaks come from the plus and how many peaks come from the minus strand, i.e., are there any potential mispriming events in the read libraries or does the peakcaller have a strand bias? (4,5) How many peaks overlap with 3'UTRs and how many peaks overlap with 5'UTRs, i.e., is there a protocol bias (e.g., restriction enzyme bias) or peakcaller problem that leads to an unusual amount of peaks in these regions? We tested further the specificity of the peakcallers, since the number of peaks between the different tools vary. We therefore checked (6) how many peaks overlap with repeat regions, (7) especially with intron repeats, and how many peaks overlap with pseudogenes of (8) ncRNA, (9) rRNA, and (10) tRNA, like reviewer 2 has suggested. The results are written in section "Effects of Using Different Peakcallers for RBFOX2".

We have done almost the same thing for the protein SLBP (Supplementary Table 4), because of the suggestion of reviewer 2. We also checked the number of peaks overlapping with histone genes and their UTRs, because SLBP targets mainly histone RNAs. The results are written in the section "Comparison of CLIP-Explorer's Results".

We also checked the peak length and the distance between the peaks of the different peakcallers for the RBFOX2 data (Supplementary Figure 3). We put the results in the section "Effects of Using Different Peakcallers for RBFOX2". We cannot make a general judgement about a good value for the peak length, because each RBP and each binding region can be specific and a benchmarking dataset is missing for CLIP-Seq data. Furthermore, the distances between the peaks are almost identical between the peakcallers PEAKachu, PureCLIp and CLIPper. Only Piranha has slightly more peaks that are closer together, indicating that Piranha might have many false positives because it calls many peaks (local maxima) in close proximity and does not combine them into a bigger peak (global maximum).
\end{answer}

Another key question that the authors should answer is how the pre-processing affect the peak caller tools? CLIP-explorer use 3 peak caller tools to call peaks, what would change running these stand alone tools compared to running them within CLIP-explorer?

\begin{answer}
We checked the behavior of the different peakcallers (PEAKachu, Piranha, and PureCLIP) based on a different pre-processing. We took the hg19 alignments of RBFOX2 (ENCFF154BQS, ENCFF994WPX, ENCFF590UCY) from ENCODE of the pipeline from the Nostrand et al. paper and ran the peakcallers on that data. We put the results in section "Effects of Using Different Peakcallers for RBFOX2".

The full main motif of RBFOX2 with the sequence \textit{UGCAUG} was identified only by PureCLIP. With CLIP-Explorer all three peakcallers found the main motif with lower background noise. Furthermore the motif set from CLIP-Explorer looks more similar between all three peakcallers.

\end{answer}

The choice of the multiple datasets used in the manuscript should be clearly stated in the methods or results section. Possibly a summary table would help the reader to keep track of the different analysis and dataset used.

\begin{answer}
We made a short overview paragraph in the methods section called "Analyzed Data".
\end{answer}

Overall the manuscript require a reorganization of the different sections and it is often redundant and repetitive. For instance the peak callers comparison should be mentioned in the first place in order to select the "best" tool to use in afterwards within the galaxy framework. Then how the pre-processing affect the peak calling step, in order to show (or not) the advantages to use the peak callers within

CLIP-explorer respect to the stand alone version. Finally the results of the application of CLIP-explorer on several dataset.

\begin{answer}
We have rearranged and pruned the paper.
\end{answer}

Another concern regards the flexibility of CLIP-explorer: the authors state in the manuscript the CLIP-seq analysis tools change over time and new tools that outrank the chosen ones may happen. How the authors would face this challenge? How flexible is CLIP-explorer to include new tools, change parameters etc?

\begin{answer}
The flexibility of CLIP-Explorer can be seen and already tested with Galaxy. A tutorial or description of Galaxy would be beyond the scope of the paper (see https://galaxyproject.github.io/training-material/). However, tools and tool versions can be easily exchanged in CLIP-Explorer (see https://galaxyproject.github.io/training-material/topics/introduction/tutorials/galaxy-intro-101/tutorial.html for a tutorial about Galaxy's workflows). It is a simple click to remove and a new click to add a new tool. The new tool can be connected to the workflow with simple drag and drop actions. All important tool parameters are well described and the user can change them without the knowledge of any programming language or the use of the terminal. Figuratively, the user can use bioinformatic tools in an environment that is more suited for a reproducible and user-friendly data analysis.
\end{answer}

Minor revision:

Table 1 is not easy readable and takes a lot of space. Perhaps it would be better having the peak callers on the columns instead of the rows, this would allow easier comparisons of motifs.

\begin{answer}
The layout of Table 1 was improved to make it more readable and concise. It also includes now the peakcaller results for a different data pre-processing.
\end{answer}

The paragraph "verifying known facts of RBFOX2" goes beyond the aims of the paper and lead no strong conclusions to support the original question.

\begin{answer}
We moved the text into section "Effects of Using Different Peakcallers for RBFOX2" and adapted the text slightly to fit the content better. We think it is still relevant to show the versatility and credibility of CLIP-Explorer. We understand that the paper is not supposed to show any highly biological relevance to RBFOX2, but we reckon as a proof of concept, that is to say, we find the same observation as stated in previous studies, showing what CLIP-Explorer is capable of.
\end{answer}

\section{Reviewer \#2}

The manuscript by Heyl et al describe a data analysis pipeline for processing CLIP-seq data that enables simplified comparison between peak callers and analysis options using the Galaxy server and structure. The development of improved (and standardized) CLIP-seq peak calling and analysis tools is of great utility to the CLIP field, and CLIP-explorer seems like it has appealing characteristics as a Galaxy implementation that will extend usability to non-experts (and the extensive tutorial and documentation provided in the associated Galaxy pipeline are well-done). As Gigascience has open reviews, I can note here that I am one of the primary contributors to the ENCODE eCLIP resource.

I have two main areas of concern with the current manuscript:

First, with respect to the analysis of CLIPper peaks provided by the ENCODE consortium:

$>>$In addition, CLIPper sometimes misses important motifs in comparison to CLIP-Explorer. CLIPper does not find the known motif UGCAUG of RBFOX2 [21, 28, 29] and GGAGA of LIN28B [47] (Supplements: Table 1).

With respect to the authors, I think there may be some confusion in the analysis here, as this is in direct contradiction to the original eCLIP paper (PMID 27018577; Supplementary Figures 3F discussing an RBFOX2 293T dataset) and my experience with the ENCODE data (and the RBFOX2 datasets in particular).

I do not see in the Methods section any discussion of significance (p-values and/or fold-enrichment in IP versus input) - the eCLIP paper discussed that (CLIPper-identified) peaks that were not significantly enriched in IP versus input did not contain the RBFOX2 motif and should not be included in downstream analysis, but I believe (in many sections in the manuscript) that the authors use all CLIPper-identified clusters rather than only the subset that are significantly enriched (based on the \# of peaks listed in the text).

I also am not clear if motif analysis was performed on the CLIPper peaks only, or whether this analysis also included flanking regions (this is not mentioned in the text, though in the associated galaxy tutorial there is a step that adds 20nt to each side of peaks prior to motif analysis, but I am unclear if this was performed for CLIPper peaks as well).

Although I am not as familiar with the MEME-ChIP software the authors use, when I run that software on the CLIPper peaks from the RBFOX2 dataset (specifically ENCFF639MYI.bed , which is the RBFOX2 HepG2 replicate 1 peak bed file):

MEME-ChIP -o /outputdirectory/ -dna -seed 123 -filter-thresh 0.05 -norc -meme-mod zoops -meme-minw 5 -meme-maxw 20 -meme-nmotifs 20 -dreme-e 0.05 -dreme-m 5 ENCFF639MYI.bed.fa

I agree that there is no visible RBFOX2 motif enriched (attached image)

However, as the authors note, many peak callers (including CLIPper) often call peaks that terminate prior to the motif itself (due to reverse transcription termination within the motif, which causes the read density to drop within or 3' of the motif). If the CLIPper peaks are extended by 10nt on the 5' end only, the canonical RBFOX motif then becomes more clearly enriched (attached image)

More critically, however, if this is done using IP versus input enrichment cutoffs recommended in the ENCODE papers (log2(foldenrichment) $>=$ 3 and -log10(p-value) $>=$ 3), the RBFOX2 motif is recovered even without this 10nt extension (attached image)

And becomes more clearly enriched with the 10nt extension (attached image)

Particularly as the authors are comparing peak callers that include an input normalization step, it seems more appropriate to use the input-normalized CLIPper peaks for comparative analysis here.

\begin{answer}
For the analysis in the manuscript, we took the peaks from the IDR (signal normalization), for example, RBFOX2 with ENCFF154DRN (hg19). However indeed, we performed the motif analysis on the CLIPper peaks only without the inclusion of the flanking regions. We assumed that this was already done to the CLIPper peaks. Therefore, we repeated the analysis including a 10nt extension. Furthermore, we found a bug in the CrossMap tool (for hg19 to hg38 or vice versa genomic location conversion) and fixed it. We can therefore verify the observations of reviewer 2 and made the appropriate changes.

Thus, the Supplements 1 and 2 and the results in section "Comparison of CLIP-Explorer Results" have changed.
\end{answer}

My other concern with the manuscript as written is that a lot of emphasis describes differences (or similarities) in CLIP-explorer output versus other peak callers, but the discovery of additional peaks and novel features (motifs, binding patterns, etc) are often assumed as positive and unique insights rather

than as potential evidence of false-positive signal. As the main purpose of this manuscript is not a deep comparison of peak callers but rather the implementation of the CLIP-Explorer pipeline, I think it would be more appropriate to present both of these options except where some independent validation can be described.

\begin{answer}
Thank you very much for this point. It is true that we did not aim at a deep comparison, but tried to sensitize for possible differences in the results analyzing CLIP-Seq data with different peakcallers. We therefore state now clearly and more than once in the paper, because of a missing benchmarking set (ground truth) it is not guaranteed that each peakcaller works impeccable, that is to say, all peakcallers (PEAKachu, Piranha, PureCLIP, CLIPper) might have false positives and false negatives. We mention this in section "Recommendations for PEAKachu, Piranha, and PureCLIP", stating: "To this day, a benchmarking dataset for CLIP-Seq data analysis does not exist because of missing experimental methods to verify predicted binding sites. It is therefore recommended to test more than one peakcaller and to test more than one parameter set, which is easily possible with CLIP-Explorer.", and again in section "Effects of Using Different Peakcallers for RBFOX2", where we say: "It is therefore possible to find a better ground truth, that is to say, a benchmark set with CLIP-Explorer, since more than one peakcaller can be tested with just a few clicks. A simple exchange with a different peakcaller is often not supported by other pipelines such as iCount."; and finally in the "Potential implications": "Based on our results we recommend to test more than one peak calling algorithm for other RBPs to assess the robustness of the motifs. CLIP-Explorer allows this very easily, because of its user-friendly interface. An exchange of the peakcaller can be done in an instant.".

We also now included more independent validations to stress out potential false positives in the peak sets of PEAKachu, Piranha, and PureCLIP and make clear that it is not easy to say which peakcaller performs "better". We stress this out in the discussion of the results of RBFOX2 in section "Effects of Using Different Peakcallers for RBFOX2" and in the discussion of the results for the RBP SLBP in section "Comparison of CLIP-Explorer's Results".
\end{answer}

For example,
$>>$"Several of the new motifs detected by CLIP-Explorer are also detected by CLIPper but not covered by the databases [43, 44] or any other literature to the best of our knowledge (Supplements: Table 1). In more detail, we find the new sequence motif CAGGCUGG for PTBP1, whereas LIN28B has the additional sequence motif CAGCCUG or the short motif CUCA. Finally, we find additional binding motifs for U2AF2 (ACAG) and QKI (AGGCU)."

The reliability of these motifs would be increased if the authors can present independent evidence (affected genes from knockdown RNA-seq, weaker enrichment in in vitro binding assays, etc) for at least one of these motifs to validate that these are not an unexplored source of false-positives. This is particularly true for cases where the RBP has a well-characterized binding modality (e.g., SLBP binds a highly conserved hairpin structure, and so the fact that CLIP-Explorer identifies significantly more peaks which enable identification of significant primary sequence motifs raises the question of whether these represent true binding motifs)

\begin{answer}
We checked the motif of PTBP1, as recommended, using independent data from ENCODE from a knockdown and control experiment. A new figure (Figure 3) shows the CDF of the log2 fold changes of the genes of hg38. The results are described as a validation in the section "Comparison of CLIP-Explorer’s Results" and the analysis is described in the method section "Analyzed Data". We could show that both PEAKachu and CLIPper peaks (binding sites) are probably true binding sites, as indicated by a significant shift of the CDF of the log2 fold changes in comparison to the non-targeted genes. The targets with the motif CAGGCUGG (from CLIP-Explorer) and with the motif CCAGGCUG (from CLIPper) show a significant shift as well, however not as prominent as the whole target ensemble of CLIP-Explorer and CLIPper. We also checked a different motif (UUCCUUUC), which was also identified by CLIP-Explorer and CLIPper. The motif seemed more significant then the motif CAGGCUGG. However, it also had more targets (715) than the motif CAGGCUGG (304) and CCAGGCUG (292). We can only speculate why the targets of the motif CAGGCUGG are not more influenced by the knockdown. We have not done the knockdown experiment and cannot make any statements about the quality or the results of the experiment. However, we can clearly state that all targets with the three motifs are significantly affected.
\end{answer}

Similarly:
$>>$For example, CLIPper finds 158 peaks and CLIP-Explorer (PEAKachu) 1052 for the protein SLBP. CLIP-Explorer predicts almost seven times more peaks than the CLIPper pipeline. We suspect that this difference is due to the computational model of CLIPper, which calls peaks for every replicate separately. Thus, we check the number of predicted peaks of the CLIPper pipeline for each replicate. The first replicate encompasses 9194 peaks, whereas the second replicate has 11686 peaks, which makes a total difference of 2492 peaks. Intersecting the peaks, without an irreducible discovery rate (IDR), of the two replicates with bedtools (see Methods) results in 1136 peaks.

The authors seem to imply in this section that the increased number of peaks correlates with quality; but as SLBP is well-characterized to exclusively bind to a hairpin structure within histone 3'UTRs (and there are only ~150 histone genes, which may include pseudogenes) it is unclear whether >1000 peaks is reasonable for SLBP. It would be helpful to include further analysis here to query whether these additional peaks represent true peaks that make sense biologically, or rather signal a potential source of false-positive signal. Are these additional peaks at (or near) histone 3'UTRs, or are they randomly distributed across other genes (or particularly at abundant noncoding RNAs or other locations that are common artifacts in many CLIP experiments)?

(It should be noted here that I believe the '9194 and 11686' numbers reflect CLIPper peaks without including a fold-enrichment or p-value cutoff for IP versus input, whereas the IDR peaks provided by the ENCODE project include these cutoffs as part of the IDR processing pipeline; so the larger numbers (clusters identified by CLIPper prior to input normalization) are not comparable to other peak callers that include input normalization.)

\begin{answer}
We did not intend to make any implications or quality assessment with this paragraph. It was just the description of the observed differences between peak sets of the two pipelines, that is to say, more importantly between the two peakcallers PEAKachu and CLIPper. Thus, we extended the analysis to find out what might cause the difference in peak numbers without making any quality assessments. Furthermore, we slightly changed the wording from the previous paper to not mislead the reader into thinking that either PEAKachu or CLIPper "performs better".
\end{answer}

Along these lines, for the comparisons between peak callers, I would be more interested in deeper analysis of the distinct peaks identified rather than all peaks. For Figure 2, for example - I would be particularly interested to see the distribution in Fig 2b shown not for all peaks, but for the 7714/177/10589 peak caller-specific peaks shown in Fig 2a, as I think those would be most informative as to which peak caller is 'best'. Do the authors see fundamental differences in the peak-caller-specific peaks (i.e., does Piranha or PEAKachu tend to over-call peaks in repetitive elements in introns, to split broader peaks into many neighboring short peak calls, etc)?

\begin{answer}
We took the recommendation an adapted figure 2b. We investigated the different target distributions for the individual peakcallers (O; 7714/177/10589) and the peak set of the intersection with all peakcallers (I; 2134/2117/2769). We describe and discuss the results in section "Effects of Using Different Peakcallers for RBFOX2" of the paper, as we think this is an interesting question for the reader.

As stated now in the paper:
The target distribution of Piranha and PureCLIP changes for the individual peak set. The portion of intron coverage shrinks and the portion of exon targets increases as well as lincRNA, rRNA, snoRNA, and snRNA. In contrast, the target distributions of PEAKachu looks similar. However, the target distribution of the intersection of all three peakcaller looks different in comparison to the distribution of all peaks. PEAKachu's distribution has a smaller intron and a bigger exon and pseudogene portion. On the other hand, the peaks from the intersection set of Piranha and PureCLIP overlap more with introns and less with exons or any snRNA, snoRNA, tRNA, or rRNA.

We conclude from these observations:
a) The higher false positive rate in the Piranha peak set, since the peaks might cover false positives that lie in unspecific regions such as rRNAs.
b) False positives in the PureCLIP peak set, even though it might be more robust than Piranha, because of the change of the target distribution for the PureCLIP peaks that do not intersect with the peaks of

the other two peakcallers. Like Piranha's distribution, peaks might overlap with unspecific regions such as rRNAs.
c) False positives in the PEAKachu peak set. As the paper states now: "PEAKachu's target distribution for peaks that do not intersect with the peaks of the other two peakcallers might also suggest some false positives, because of the bigger portion of peaks in pseudogenes in that peak set."

As also mentioned for reviewer 1, because of a missing ground truth, we validated the peakcaller now more on biological facts than technical features (e.g., false positives or accuracy). For that reason, we put a new Table and new results into the supplements of the paper (Supplementary Table 3 and 4).
\end{answer}

This is a particularly important question as the methods indicates the authors use a one-step mapping to the human genome, rather than the two-step approach the ENCODE pipeline used for eCLIP analysis (first removing repetitive elements by mapping against a database of RepBase and other elements, then followed by standard genomic mapping with STAR that requires unique mapping). I wonder if this is a major contributor to the large differences in number of identified peaks - are many of these peak caller-specific peaks at rRNA/tRNA/ncRNA pseudogenes throughout the genome?

\begin{answer}
We refer at this point to the aforementioned changes which also include a deeper analysis of the SLBP peaks based on rRNA/tRNA/ncRNA pseudogenes.
\end{answer}

Minor comments:

Fig. 2b would be more informative with a different color scheme, as it's hard to identify quickly which color is which

\begin{answer}
We changed the color scheme and we hope it is more distinguishable now.
\end{answer}

$>>$However, CLIP-Explorer maps the reads to hg38, except for RBFOX2 (hg19), whereas the pipeline of the study by Nostrand et al. [13] used hg19. We therefore convert the peak coordinates of the CLIPper algorithm from hg19 to hg38 with CrossMap [45].
Although I think this is likely fine for the analysis described here, it should be noted that the ENCODE DCC has available pipeline output for both hg19 and hg38 genome versions (e.g. ENCFF390PJW for replicate 1 peak calls for RBFOX2 HepG2 in hg38)

\begin{answer}
For the sake of reproducibility and correctness, we used now directly the CLIPper peaks of hg38 from ENCODE, except for RBFOX2, where we used the hg19 peaks. We have changed the paragraph in section "CLIP-Explorer: A Versatile Pipeline for the Analysis of CLIP-seq Data" (removing the statement about CrossMap). The results have changed because of it. It had some meaningful impact because of a bug in CrossMap, thanks to your comment. That is to say, the motifs of CLIPper are different (see Supplements 1) and the peak intersections with PEAKachu and CLIPper (see Supplements 2).
\end{answer}

\section{Remark}

\begin{answer}
We highlighted the major changes in the paper red. Major changes involve the recommendation and questions of the reviewer. Other changed such as rearrangements of whole paragraphs and changes of figures are not highlighted and need to be checked again. However, they are mentioned in the answers of the reviewers.

Figures and Supplements that changed: Figure 2, Supplements 1 and 2.

We thank reviewer 1 and 2 for their ideas, recommendations and questions that improved the manuscript. We thank both reviewers for their time and wish them both a good day.
\end{answer}

Close
