# Supplementary material for: Galaxy CLIP-Explorer: a web server for CLIP-Seq data analysis
Source: Gigascience. 2020 Nov 11;9(11):giaa108. doi: 10.1093/gigascience/giaa108 (PMC7657819; doi:10.1093/gigascience/giaa108)
Supplement: giaa108_Response_to_Reviewer_Comments_Revision_1 [file giaa108_response_to_reviewer_comments_revision_1.pdf]

Author's Response To Reviewer Comments

Close

\section{Reviewer 1:}
The manuscript significantly improved and is now ready for publication.
The only statement with which I do not agree is the absence of CLIP-seq tools benchmarks: there are several in the literature, here a couple of examples:
Bottini et al. 2017 NAR "From benchmarking HITS-CLIP peak detection programs to a new method for identification of miRNA-binding sites from Ago2-CLIP data"
Park et al. 2018 NAR "CLIPick: a sensitive peak caller for expression-based deconvolution of HITS-CLIP signals"
These works and others should be mentioned in the paper and the statement changed accordingly.

\begin{answer}
Many thanks for this comment.

We included the cited papers and other articles for peak calling benchmarking in the section "Identification of Enriched Regions and Sequence Motifs". We want to stress out at that we would like to integrate and thus give the opportunity for the user to be able to choose other peak callers in our pipeline, such as, omniCLIP and CLIPper, but these tools are so far not supported by conda or miss an open source license, but CLIP-Explorer is constantly updated with new tools and tool versions.
\end{answer}

\section{Reviewer 2:}
The manuscript by Heyl describes a software framework to perform analysis of CLIP datasets in the Galaxy platform, incorporating numerous different software tools and peak callers. As CLIP analysis remains an open question that is often challenging for non-expert users, the development of easy to use frameworks is of high interest.

I appreciate the author's edits throughout, particularly with respect to the CLIPper peak calls and motif analysis. With respect to the discussion of comparing peak callers, though, I think there still needs to be more care in separating out what are reasonable comparisons between datasets versus what results are either surprising or concerning and conflict with existing literature.

Major concerns

1) I'm very confused by the 'Plus Strand / Minus Strand' in Table 1 (and related text). Are those just referring to genomic Watson versus Crick strand? As far as I'm aware, every CLIP method has a stranded library preparation method, so those numbers should be nearly 100\% plus strand (with plus defined as the sense strand of the annotated gene).

\begin{answer}
It is true that the reads are nearly 100\% from the plus strand. It seemed that this feature was quite confusing. After further consideration, we removed this feature from the paper, since it had no important impact on the paper. We have changed table number one and supplementary table number 4.
\end{answer}

2) In particular, with respect to the number of peaks and SLBP analysis and the authors response:
"We did not intend to make any implications or quality assessment with this paragraph. It was just the description of the observed differences between peak sets of the two pipelines, that is to say, more importantly between the two peakcallers PEAKachu and CLIPper. Thus, we extended the analysis to find out what might cause the difference in peak numbers without making any quality assessments. Furthermore, we slightly changed the wording from the previous paper to not mislead the reader into thinking that either PEAKachu or CLIPper "performs better"."
In other responses the authors note that "We therefore state now clearly and more than once in the paper, because of a missing benchmarking set (ground truth) it is not guaranteed that each peakcaller

works impeccable, that is to say, all peakcallers (PEAKachu, Piranha, PureCLIP, CLIPper) might have false positives and false negatives.", but this is my main point with SLBP - it actually does provide a reasonable ground truth set, as to my knowledge previous genomic and mechanistic studies of SLBP indicate that it exclusively binds to histone 3'UTRs (e.g. PMID 26377992). Thus, SLBP provides a unique opportunity to actually benchmark these pipelines (and conversely, in this context I think it's misleading to simply compare the number of peaks for SLBP from these different pipelines without actually making a quality assessment).
Going through this section:
For example, CLIP- per finds 161 peaks and CLIP-Explorer (PEAKachu) 1052 for the protein SLBP. Using PEAKachu, CLIP-Explorer predicts almost seven times more peaks than the CLIPper pipeline.

This PEAKachu result suggests a nearly 90\% false positive rate without some evidence that the non-histone peaks are real.

\begin{answer}
We have refined the peak set from PEAKachu for SLBP. That is why Supplementary Table 4 changed. Indeed PEAKachu found in the default setting way more false positives for SLBP than CLIPper. We then investigated the parameter setting of CLIPper (CLIPper pipeline) in more detail. Thus, we setup a parameter set for PEAKachu that matches the specificity settings of CLIPper. We adjusted the parameters for this particular protein and filtered for peaks that are in non-repeat and annotated regions, which are additional steps performed by the CLIPper pipeline. With this modifications, we got in total 514 peaks with the new parameter set, which we filtered for repeats (272 peaks), and finally for peaks in annotated regions (final peaks set with 220 peaks).

PEAKachu still finds more putative false positives (overall more peaks on ncRNAs), under the assumption that SLBP targets only histone mRNAs, but it also finds generally more peaks in histone regions than CLIPper and based on the overlap with the CLIPper peaks (see Supplementary Material 2) we know that these peaks are unique to PEAKachu. We looked into the histone transcripts covered by the CLIPper (45 transcripts) and PEAKachu peaks (57 transcripts), out of which 42 are covered by both, 3 are covered only by CLIPper (2 are pseudogenes), and 15 are covered only by PEAKachu (3 are pseudogenes).

It is true that SLBP can be used to better benchmark CLIP-Seq pipelines and peakcallers. Other proteins like PTBP1 are harder to evaluate and might require a different annotation file or other stringent filter criteria for the peak set. Because of the deeper analysis with the SLBP data, we have two parameter sets for PEAKachu, one which can be initially used to get a genome/transcriptome-wide overview of potential binding sites, and another more specific and stringent parameter set to filter for highly significant binding sites.

In addition, we would like to integrate CLIPper in CLIP-Explorer to provide the user with more alternatives. The tool got recently (a few days ago) an no open source license but still miss a conda build and is thus not supported by Galaxy. Peak calling is so far the most crucial step in CLIP-seq data analysis and thus it is advantageous if the user has different options.
\end{answer}

We suspect that this difference is due to the computational model of CLIPper, which calls peaks for every replicate separately. Thus, we check the number of predicted peaks of the CLIPper pipeline for each replicate. The first replicate encompasses 9194 peaks, whereas the second replicate has 11686 peaks, which makes a total difference of 2492 peaks.

I believe these numbers are all still reflecting raw CLIPper clusters, and do not include fold-enrichment cutoffs as the eCLIP Nature Methods paper suggests - indeed that paper indicated that CLIPper alone (without including the IP versus input normalization) gives a high fraction of artifact clusters at abundant transcripts. The results here should be compared against the 'state of the art', which (at least to me) requires at least some fold-enrichment and significance cutoffs.

\begin{answer}
We made a change to the statement in results section:

The first replicate encompasses $7,942$ CLIPper peaks and the second replicate $1,530$ peaks, when we filter for entries with a p-value of 0.05 and a log2 fold change of 1, which makes a total difference of $6,412$ peaks. Intersecting the peaks without an irreducible discovery rate (IDR) results in 291 peaks, more than PEAKachu, and with an IDR CLIPper has the aforementioned 161 peaks, so less

than PEAKachu.

We also changed the statement in the discussion section.
\end{answer}

…Both PEAKachu and CLIPper have almost 60\% of peaks from the plus strand and less than 1\% of peaks from 3' or 5' UTRs.

These numbers would be extremely concerning to me - the known function of SLBP is targeted to histone 3'UTRs. Some bleed-through into the CDS region is reasonable, but the authors need some description here of why this result suggests that the peaks used are of high quality.

\begin{answer}
As aforementioned we have removed the strand feature from the table and also the statement in the text. We also removed the 3' UTR histone feature. We tried to find a complete annotation for 3' histone UTRs (Ensembl, UCSC) but the annotation we took covered only 105 histone transcripts and missed a lot of known histone transcripts. Thus, we decided to remove this feature from the paper.
\end{answer}

However, PEAKachu has some peaks (~ 16.25\%) in repeats, which do not overlap with intron repeats, and it has some peaks in ncRNA pseudogenes (~ 0.022\%) and one peak that overlaps with an rRNA pseudogene. PEAKachu also finds more peaks that overlap with ncRNA (321 peaks) than CLIPper (17 peaks).

Again, this suggests that PEAKachu has a much higher false positive rate.

\begin{answer}
Because of the changes through the major revision, this part changed in the paper because of the stricter parameter set and filtering for PEAKachu stating:

Both PEAKachu and CLIPper have no peaks in repeat or pseudogene regions, except for one PEAKachu peak in a ncRNA pseudogene. PEAKachu also finds more peaks that overlap with ncRNA genes (48 peaks) than CLIPper (17 peaks). We also check the number of peaks overlapping with histone genes since SLBP targets mainly histone RNAs. PEAKachu finds slightly more peaks in histone regions (163 peaks) than CLIPper (135 peaks). Consequently, PEAKachu covers slightly more peaks in histone regions, but also more peaks in ncRNAs genes, which might represent false positives since SLBP is known to target histone mRNA. The eCLIP pipeline from Nostrand et al. \cite{van2016robust} already removed repeat regions in a double mapping approach.
\end{answer}

Minor issues:

1. The authors discuss 'peaks from method A overlapping with peaks from method A' multiple times, but I'm not very clear on what the interpretation is intended from this analysis. Are the authors comparing peaks between biological replicates? Or just within the same dataset? What does the finding of overlapping peaks mean within a dataset based on the underlying properties of the peak calling algorithm? Because I know more about the details of CLIPper - in the underlying method, CLIPper does not call overlapping peaks per transcript (although it does call many that neighbor each other); thus any overlapping peaks within a dataset are due to peak calls derived from different overlapping transcripts (that then have slightly different background models). In that context, I'm not sure what the number of overlapping peaks means, other than some rough correlate of how many of the targets have many versus few annotated isoforms (which isn't really a feature of the peak caller).

\begin{answer}
We have removed this feature from both tables (table one and supplementary table four) because it led to some confusion and had no real contribution to the paper.

Our initial motivation for this feature was to investigate how many peaks overlap with other peaks in the same peak set, that is to say, how many peaks probably cover the same transcripts. However, since a peak profile can consist of different local minima, which probably correspond to more than one binding site in a transcript, the feature was a bit unclear and shallow, in addition to the confusing explanations

in the paper.
\end{answer}

2. "We found that roughly only 10\% of the CLIPper peaks were contained in both replicates. This variation between replicates is in agreement with the literature [52]." - I believe this number is again using no fold-enrichment or significance cutoffs in IP versus input, which should be explicitly stated.

\begin{answer}
We changed the statement in the discussion to:

The first replicate encompasses $7,942$ CLIPper peaks and the second replicate $1,530$ peaks, when we filter for entries with a p-value of 0.05 and a log2 fold change of 1, which makes a total difference of $6,412$ peaks. Intersecting the peaks without an irreducible discovery rate (IDR) results in 291 peaks, more than PEAKachu, and with an IDR CLIPper has the aforementioned 161 peaks, so less than PEAKachu. Thus, roughly 20% of the CLIPper peaks in replicate one are contained in replicate two. Consequently, the difference in the peak set of PEAKachu (CLIP-Explorer) might come from a different noise estimation between replicates, thus detecting other binding sites.
\end{answer}

\section{Remark}

We thank the reviewers for their contribution, ideas and critics as it honed the paper even further. Every change that was made to the manuscript is highlighted in red.

Supplementary Material 1 (E-values of PEAKachu for SLBP), 2 (Venn diagram of SLBP), and table 4 changed.

Close
