# Supplementary material for: Galaxy CLIP-Explorer: a web server for CLIP-Seq data analysis
Source: Gigascience. 2020 Nov 11;9(11):giaa108. doi: 10.1093/gigascience/giaa108 (PMC7657819; doi:10.1093/gigascience/giaa108)
Supplement: giaa108_Response_to_Reviewer_Comments_Revision_2 [file giaa108_response_to_reviewer_comments_revision_2.pdf]

Author's Response To Reviewer Comments

Close

Reviewer #2: I support publication and I think this will be a very useful tool!

Minor comment: the Nostrand et al. [13] callouts should be "Van Nostrand et al. [13]"

Answer: We have changed the callouts to the correct format.

We thank all reviewers for their time and input to complete the paper.

Close
