# Supplementary material for: Galaxy CLIP-Explorer: a web server for CLIP-Seq data analysis
Source: Gigascience. 2020 Nov 11;9(11):giaa108. doi: 10.1093/gigascience/giaa108 (PMC7657819; doi:10.1093/gigascience/giaa108)
Supplement: giaa108_Reviewer_1_Report_Original_Submission — Silvia Bottini -- 11/19/2019 Reviewed [file giaa108_reviewer_1_report_original_submission.pdf]

Reviewer Report

Title: Galaxy CLIP-Explorer: a web server for CLIP-Seq data analysis

Version: Original Submission **Date:** 11/19/2019

Reviewer name: Silvia Bottini

Reviewer Comments to Author:

The authors proposed a new web server for CLIP-seq data analysis. Although the work is sound and interesting, I have few suggestions/concerns that might improve the work.
Major revisions:
The authors propose specific tools for each step of the pipeline, however they missed to explain why they made this choice. The authors missed to cite recent CLIP-seq tools benchmark and to put their work in relationship with these previous related papers. Furthermore the benchmark of peak calling tools should be extended, including exact metrics to evaluate the comparisons of the performances of the selected tools.
Another key question that the authors should answer is how the preprocessing affect the peak caller tools? CLIP-explorer use 3 peak caller tools to call peaks, what would change running these stand alone tools compared to running them within CLIP-explorer?
The choice of the multiple datasets used in the manuscript should be clearly stated in the methods or results section. Possibly a summary table would help the reader to keep track of the different analysis and dataset used.
Overall the manuscript require a reorganization of the different sections and it is often redundant and repetitive. For instance the peak callers comparison should be mentioned in the first place in order to select the "best" tool to use in afterwards within the galaxy framework. Then how the preprocessing affect the peak calling step, in order to show (or not) the advantages to use the peak callers within CLIP-explorer respect to the stand alone version. Finally the results of the application of CLIP-explorer on several dataset.
Another concern regards the flexibility of CLIP-explorer: the authors state in the manuscript the CLIP-seq analysis tools change over time and new tools that outrank the chosen ones may happen. How the authors would face this challenge? How flexible is CLIP-explorer to include new tools, change parameters etc?
Minor revision:
Table 1 is not easy readable and takes a lot of space. Perhaps it would be better having the peak callers on the columns instead of the rows, this would allow easier comparisons of motifs.
The paragraph "verifying known facts of RBFOX2" goes beyond the aims of the paper and lead no strong conclusions to support the original question.

Methods

Are the methods appropriate to the aims of the study, are they well described, and are necessary controls included? Choose an item.

Conclusions

Are the conclusions adequately supported by the data shown? Choose an item.

Reporting Standards

Does the manuscript adhere to the journal's guidelines on minimum standards of reporting? Choose an item.

Choose an item.

Statistics

Are you able to assess all statistics in the manuscript, including the appropriateness of statistical tests used? Choose an item.

Quality of Written English

Please indicate the quality of language in the manuscript: Choose an item.

Declaration of Competing Interests

Please complete a declaration of competing interests, considering the following questions:

- Have you in the past five years received reimbursements, fees, funding, or salary from an organisation that may in any way gain or lose financially from the publication of this manuscript, either now or in the future?
- Do you hold any stocks or shares in an organisation that may in any way gain or lose financially from the publication of this manuscript, either now or in the future?
- Do you hold or are you currently applying for any patents relating to the content of the manuscript?
- Have you received reimbursements, fees, funding, or salary from an organization that holds or has applied for patents relating to the content of the manuscript?
- Do you have any other financial competing interests?
- Do you have any non-financial competing interests in relation to this paper?

If you can answer no to all of the above, write 'I declare that I have no competing interests' below. If your reply is yes to any, please give details below.

I declare that I have no competing interests

I agree to the open peer review policy of the journal. I understand that my name will be included on my report to the authors and, if the manuscript is accepted for publication, my named report including any attachments I upload will be posted on the website along with the authors' responses. I agree for my

report to be made available under an Open Access Creative Commons CC-BY license (http://creativecommons.org/licenses/by/4.0/). I understand that any comments which I do not wish to be included in my named report can be included as confidential comments to the editors, which will not be published.

Choose an item.

To further support our reviewers, we have joined with Publons, where you can gain additional credit to further highlight your hard work (see: https://publons.com/journal/530/gigascience). On publication of this paper, your review will be automatically added to Publons, you can then choose whether or not to claim your Publons credit. I understand this statement.

Yes Choose an item.
