# Supplementary material for: Galaxy CLIP-Explorer: a web server for CLIP-Seq data analysis
Source: Gigascience. 2020 Nov 11;9(11):giaa108. doi: 10.1093/gigascience/giaa108 (PMC7657819; doi:10.1093/gigascience/giaa108)
Supplement: giaa108_Reviewer_2_Report_Original_Submission — Eric Van Nostrand -- 11/22/2019 Reviewed [file giaa108_reviewer_2_report_original_submission.pdf]

Reviewer Report

Title: Galaxy CLIP-Explorer: a web server for CLIP-Seq data analysis

Version: Original Submission **Date:** 11/22/2019

Reviewer name: Eric Van Nostrand

Reviewer Comments to Author:

The manuscript by Heyl et al describe a data analysis pipeline for processing CLIP-seq data that enables simplified comparison between peak callers and analysis options using the Galaxy server and structure. The development of improved (and standardized) CLIP-seq peak calling and analysis tools is of great utility to the CLIP field, and CLIP-explorer seems like it has appealing characteristics as a Galaxy implementation that will extend usability to non-experts (and the extensive tutorial and documentation provided in the associated Galaxy pipeline are well-done). As Gigascience has open reviews, I can note here that I am one of the primary contributors to the ENCODE eCLIP resource.

I have two main areas of concern with the current manuscript:

First, with respect to the analysis of CLIPper peaks provided by the ENCODE consortium:

>>In addition, CLIPper sometimes misses important motifs in comparison to CLIP-Explorer. CLIPper does not find the known motif UGCAUG of RBFOX2 [21, 28, 29] and GGAGA of LIN28B [47] (Supplements: Table 1).

With respect to the authors, I think there may be some confusion in the analysis here, as this is in direct contradiction to the original eCLIP paper (PMID 27018577; Supplementary Figures 3F discussing an RBFOX2 293T dataset) and my experience with the ENCODE data (and the RBFOX2 datasets in particular).

I do not see in the Methods section any discussion of significance (p-values and/or fold-enrichment in IP versus input) - the eCLIP paper discussed that (CLIPper-identified) peaks that were not significantly enriched in IP versus input did not contain the RBFOX2 motif and should not be included in downstream analysis, but I believe (in many sections in the manuscript) that the authors use all CLIPper-identified clusters rather than only the subset that are significantly enriched (based on the # of peaks listed in the text). I also am not clear if motif analysis was performed on the CLIPper peaks only, or whether this analysis also included flanking regions (this is not mentioned in the text, though in the associated galaxy tutorial there is a step that adds 20nt to each side of peaks prior to motif analysis, but I am unclear if this was performed for CLIPper peaks as well).

Although I am not as familiar with the MEME-ChIP software the authors use, when I run that software on the CLIPper peaks from the RBFOX2 dataset (specifically ENCFF639MYI.bed , which is the RBFOX2 HepG2 replicate 1 peak bed file):

MEME-ChIP -o /outputdirectory/ -dna -seed 123 -filter-thresh 0.05 -norc -meme-mod zoops -meme-minw 5 -meme-maxw 20 -meme-nmotifs 20 -dreme-e 0.05 -dreme-m 5 ENCFF639MYI.bed.fa

I agree that there is no visible RBFOX2 motif enriched (attached image RBFOX2_01_all_0nt)

However, as the authors note, many peak callers (including CLIPper) often call peaks that terminate

prior to the motif itself (due to reverse transcription termination within the motif, which causes the read density to drop within or 3' of the motif). If the CLIPper peaks are extended by 10nt on the 5' end only, the canonical RBFOX motif then becomes more clearly enriched (RBFOX2_01_all_10nt)
More critically, however, if this is done using IP versus input enrichment cutoffs recommended in the ENCODE papers (log2(foldenrichment) >= 3 and -log10(p-value) >= 3), the RBFOX2 motif is recovered even without this 10nt extension (RBFOX2_01_signif_0nt)
And becomes more clearly enriched with the 10nt extension (RBFOX2_01_signif_10nt)
Particularly as the authors are comparing peak callers that include an input normalization step, it seems more appropriate to use the input-normalized CLIPper peaks for comparative analysis here.
My other concern with the manuscript as written is that a lot of emphasis describes differences (or similarities) in CLIP-explorer output versus other peak callers, but the discovery of additional peaks and novel features (motifs, binding patterns, etc) are often assumed as positive and unique insights rather than as potential evidence of false-positive signal. As the main purpose of this manuscript is not a deep comparison of peak callers but rather the implementation of the CLIP-Explorer pipeline, I think it would be more appropriate to present both of these options except where some independent validation can be described.
For example,
>>"Several of the new motifs detected by CLIP-Explorer are also detected by CLIPper but not covered by the databases [43, 44] or any other literature to the best of our knowledge (Supplements: Table 1). In more detail, we find the new sequence motif CAGGCUGG for PTBP1, whereas LIN28B has the additional sequence motif CAGCCUG or the short motif CUCA. Finally, we find additional binding motifs for U2AF2 (ACAG) and QKI (AGGCU)."
The reliability of these motifs would be increased if the authors can present independent evidence (affected genes from knockdown RNA-seq, weaker enrichment in in vitro binding assays, etc) for at least one of these motifs to validate that these are not an unexplored source of false-positives. This is particularly true for cases where the RBP has a well-characterized binding modality (e.g., SLBP binds a highly conserved hairpin structure, and so the fact that CLIP-Explorer identifies significantly more peaks which enable identification of significant primary sequence motifs raises the question of whether these represent true binding motifs)
Similarly:
>>For example, CLIPper finds 158 peaks and CLIP-Explorer (PEAKachu) 1052 for the protein SLBP. CLIP-Explorer predicts almost seven times more peaks than the CLIPper pipeline. We suspect that this difference is due to the computational model of CLIPper, which calls peaks for every replicate separately. Thus, we check the number of predicted peaks of the CLIPper pipeline for each replicate. The first replicate encompasses 9194 peaks, whereas the second replicate has 11686 peaks, which makes a total difference of 2492 peaks. Intersecting the peaks, without an irreducible discovery rate (IDR), of the two replicates with bedtools (see Methods) results in 1136 peaks.
The authors seem to imply in this section that the increased number of peaks correlates with quality; but as SLBP is well-characterized to exclusively bind to a hairpin structure within histone 3'UTRs (and there are only ~150 histone genes, which may include pseudogenes) it is unclear whether >1000 peaks is reasonable for SLBP. It would be helpful to include further analysis here to query whether these additional peaks represent true peaks that make sense biologically, or rather signal a potential source of

false-positive signal. Are these additional peaks at (or near) histone 3'UTRs, or are they randomly distributed across other genes (or particularly at abundant noncoding RNAs or other locations that are common artifacts in many CLIP experiments)?
(It should be noted here that I believe the '9194 and 11686' numbers reflect CLIPper peaks without including a fold-enrichment or p-value cutoff for IP versus input, whereas the IDR peaks provided by the ENCODE project include these cutoffs as part of the IDR processing pipeline; so the larger numbers (clusters identified by CLIPper prior to input normalization) are not comparable to other peak callers that include input normalization.)
Along these lines, for the comparisons between peak callers, I would be more interested in deeper analysis of the distinct peaks identified rather than all peaks. For Figure 2, for example - I would be particularly interested to see the distribution in Fig 2b shown not for all peaks, but for the 7714/177/10589 peak caller-specific peaks shown in Fig 2a, as I think those would be most informative as to which peak caller is 'best'. Do the authors see fundamental differences in the peak-caller-specific peaks (i.e., does Piranha or PEAKachu tend to over-call peaks in repetitive elements in introns, to split broader peaks into many neighboring short peak calls, etc)?
This is a particularly important question as the methods indicates the authors use a one-step mapping to the human genome, rather than the two-step approach the ENCODE pipeline used for eCLIP analysis (first removing repetitive elements by mapping against a database of RepBase and other elements, then followed by standard genomic mapping with STAR that requires unique mapping). I wonder if this is a major contributor to the large differences in number of identified peaks - are many of these peak caller-specific peaks at rRNA/tRNA/ncRNA pseudogenes throughout the genome?
Minor comments:
Fig. 2b would be more informative with a different color scheme, as it's hard to identify quickly whch color is which
>>However, CLIP-Explorer maps the reads to hg38, except for RBFOX2 (hg19), whereas the pipeline of the study by Nostrand et al. [13] used hg19. We therefore convert the peak coordinates of the CLIPper algorithm from hg19 to hg38 with CrossMap [45].
Although I think this is likely fine for the analysis described here, it should be noted that the ENCODE DCC has available pipeline output for both hg19 and hg38 genome versions (e.g. ENCFF390PJW for replicate 1 peak calls for RBFOX2 HepG2 in hg38)

Methods

Are the methods appropriate to the aims of the study, are they well described, and are necessary controls included? Choose an item.

Conclusions

Are the conclusions adequately supported by the data shown? Choose an item.

Reporting Standards

Does the manuscript adhere to the journal's guidelines on minimum standards of reporting? Choose an item.

Choose an item.

Statistics

Are you able to assess all statistics in the manuscript, including the appropriateness of statistical tests used? Choose an item.

Quality of Written English

Please indicate the quality of language in the manuscript: Choose an item.

Declaration of Competing Interests

Please complete a declaration of competing interests, considering the following questions:

- Have you in the past five years received reimbursements, fees, funding, or salary from an organisation that may in any way gain or lose financially from the publication of this manuscript, either now or in the future?
- Do you hold any stocks or shares in an organisation that may in any way gain or lose financially from the publication of this manuscript, either now or in the future?
- Do you hold or are you currently applying for any patents relating to the content of the manuscript?
- Have you received reimbursements, fees, funding, or salary from an organization that holds or has applied for patents relating to the content of the manuscript?
- Do you have any other financial competing interests?
- Do you have any non-financial competing interests in relation to this paper?

If you can answer no to all of the above, write 'I declare that I have no competing interests' below. If your reply is yes to any, please give details below.

I am co-founder, member of the Board of Directors, on the SAB, equity holder, and paid consultant for Eclipse BioInnovations, a genomics company developing kits and services for eCLIP and related RNA techniques. I do not believe that Eclipse presents a conflict of interest with the manuscript.

I agree to the open peer review policy of the journal. I understand that my name will be included on my report to the authors and, if the manuscript is accepted for publication, my named report including any attachments I upload will be posted on the website along with the authors' responses. I agree for my report to be made available under an Open Access Creative Commons CC-BY license (http://creativecommons.org/licenses/by/4.0/). I understand that any comments which I do not wish to be included in my named report can be included as confidential comments to the editors, which will not be published.

Choose an item.

To further support our reviewers, we have joined with Publons, where you can gain additional credit to further highlight your hard work (see: https://publons.com/journal/530/gigascience). On publication of this paper, your review will be automatically added to Publons, you can then choose whether or not to claim your Publons credit. I understand this statement.

Yes Choose an item.
