# Supplementary material for: Galaxy CLIP-Explorer: a web server for CLIP-Seq data analysis
Source: Gigascience. 2020 Nov 11;9(11):giaa108. doi: 10.1093/gigascience/giaa108 (PMC7657819; doi:10.1093/gigascience/giaa108)
Supplement: giaa108_Reviewer_2_Report_Revision_1 — Eric Van Nostrand -- 3/19/2020 Reviewed [file giaa108_reviewer_2_report_revision_1.pdf]

Reviewer Report

Title: Galaxy CLIP-Explorer: a web server for CLIP-Seq data analysis

Version: Revision 1 **Date:** 3/19/2020

Reviewer name: Eric Van Nostrand

Reviewer Comments to Author:

The manuscript by Heyl describes a software framework to perform analysis of CLIP datasets in the Galaxy platform, incorporating numerous different software tools and peak callers. As CLIP analysis remains an open question that is often challenging for non-expert users, the development of easy to use frameworks is of high interest.

I appreciate the author's edits throughout, particularly with respect to the CLIPper peak calls and motif analysis. With respect to the discussion of comparing peak callers, though, I think there still needs to be more care in separating out what are reasonable comparisons between datasets versus what results are either surprising or concerning and conflict with existing literature.

Major concerns

1) I'm very confused by the 'Plus Strand / Minus Strand' in Table 1 (and related text). Are those just referring to genomic Watson versus Crick strand? As far as I'm aware, every CLIP method has a stranded library preparation method, so those numbers should be nearly 100% plus strand (with plus defined as the sense strand of the annotated gene).

2) In particular, with respect to the # of peaks and SLBP analysis and the authors response:

\begin{answer}

We did not intend to make any implications or quality assessment with this paragraph. It was just the description of the observed differences between peak sets of the two pipelines, that is to say, more importantly between the two peakcallers PEAKachu and CLIPper. Thus, we extended the analysis to find out what might cause the difference in peak numbers without making any quality assessments. Furthermore, we slightly changed the wording from the previous paper to not mislead the reader into thinking that either PEAKachu or CLIPper "performs better".

\end{answer}

In other responses the authors note that "We therefore state now clearly and more than once in the paper, because of a missing benchmarking set (ground truth) it is not guaranteed that each peakcaller works impeccable, that is to say, all peakcallers (PEAKachu, Piranha, PureCLIP, CLIPper) might have false positives and false negatives.", but this is my main point with SLBP - it actually does provide a reasonable ground truth set, as to my knowledge previous genomic and mechanistic studies of SLBP indicate that it exclusively binds to histone 3'UTRs (e.g. PMID 26377992). Thus, SLBP provides a unique opportunity to actually benchmark these pipelines (and conversely, in this context I think it's misleading to simply compare the # of peaks for SLBP from these different pipelines without actually making a quality assessment).

Going through this section:

For example, CLIP- per finds 161 peaks and CLIP-Explorer (PEAKachu) 1052 for the protein SLBP. Using

PEAKachu, CLIP-Explorer predicts almost seven times more peaks than the CLIPper pipeline.
This PEAKachu result suggests a nearly 90% false positive rate without some evidence that the non-histone peaks are real.
We suspect that this difference is due to the computational model of CLIPper, which calls peaks for every replicate separately. Thus, we check the number of predicted peaks of the CLIPper pipeline for each replicate. The first replicate encompasses 9194 peaks, whereas the second replicate has 11686 peaks, which makes a total difference of 2492 peaks.
I believe these numbers are all still reflecting raw CLIPper clusters, and do not include fold-enrichment cutoffs as the eCLIP Nature Methods paper suggests - indeed that paper indicated that CLIPper alone (without including the IP versus input normalization) gives a high fraction of artifact clusters at abundant transcripts. The results here should be compared against the 'state of the art', which (at least to me) requires at least some fold-enrichment and significance cutoffs.
…Both PEAKachu and CLIPper have almost 60% of peaks from the plus strand and less than 1% of peaks from 3' or 5' UTRs.
These numbers would be extremely concerning to me - the known function of SLBP is targeted to histone 3'UTRs. Some bleed-through into the CDS region is reasonable, but the authors need some description here of why this result suggests that the peaks used are of high quality.
However, PEAKachu has some peaks (âˆ¼ 16.25%) in repeats, which do not overlap with intron repeats, and it has some peaks in ncRNA pseudogenes (âˆ¼ 0.022%) and one peak that overlaps with an rRNA pseudogene. PEAKachu also finds more peaks that overlap with ncRNA (321 peaks) than CLIPper (17 peaks).
Again, this suggests that PEAKachu has a much higher false positive rate.
Minor issues:
1. The authors discuss 'peaks from method A overlapping with peaks from method A' multiple times, but I'm not very clear on what the interpretation is intended from this analysis. Are the authors comparing peaks between biological replicates? Or just within the same dataset? What does the finding of overlapping peaks mean within a dataset based on the underlying properties of the peak calling algorithm? Because I know more about the details of CLIPper - in the underlying method, CLIPper does not call overlapping peaks per transcript (although it does call many that neighbor each other); thus any overlapping peaks within a dataset are due to peak calls derived from different overlapping transcripts (that then have slightly different background models). In that context, I'm not sure what the # of overlapping peaks means, other than some rough correlate of how many of the targets have many versus few annotated isoforms (which isn't really a feature of the peak caller).
2. "We found that roughly only 10% of the CLIPper peaks were contained in both repli- cates. This variation between replicates is in agreement with the literature [52]." - I believe this number is again using no fold-enrichment or significance cutoffs in IP versus input, which should be explicitly stated.

Methods

Are the methods appropriate to the aims of the study, are they well described, and are necessary controls included? Choose an item.

Conclusions

Are the conclusions adequately supported by the data shown? Choose an item.

Reporting Standards

Does the manuscript adhere to the journal's guidelines on minimum standards of reporting? Choose an item.

Choose an item.

Statistics

Are you able to assess all statistics in the manuscript, including the appropriateness of statistical tests used? Choose an item.

Quality of Written English

Please indicate the quality of language in the manuscript: Choose an item.

Declaration of Competing Interests

Please complete a declaration of competing interests, considering the following questions:

- Have you in the past five years received reimbursements, fees, funding, or salary from an organisation that may in any way gain or lose financially from the publication of this manuscript, either now or in the future?
- Do you hold any stocks or shares in an organisation that may in any way gain or lose financially from the publication of this manuscript, either now or in the future?
- Do you hold or are you currently applying for any patents relating to the content of the manuscript?
- Have you received reimbursements, fees, funding, or salary from an organization that holds or has applied for patents relating to the content of the manuscript?
- Do you have any other financial competing interests?
- Do you have any non-financial competing interests in relation to this paper?

If you can answer no to all of the above, write 'I declare that I have no competing interests' below. If your reply is yes to any, please give details below.

I am co-founder, member of the Board of Directors, on the SAB, equity holder, and paid consultant for Eclipse BioInnovations. These interests have been reviewed and approved by UCSD in accordance with its conflict of interest policies, and I do not believe they overlap with this manuscript.

I agree to the open peer review policy of the journal. I understand that my name will be included on my report to the authors and, if the manuscript is accepted for publication, my named report including any attachments I upload will be posted on the website along with the authors' responses. I agree for my report to be made available under an Open Access Creative Commons CC-BY license (http://creativecommons.org/licenses/by/4.0/). I understand that any comments which I do not wish to be included in my named report can be included as confidential comments to the editors, which will not be published.

Choose an item.

To further support our reviewers, we have joined with Publons, where you can gain additional credit to further highlight your hard work (see: https://publons.com/journal/530/gigascience). On publication of this paper, your review will be automatically added to Publons, you can then choose whether or not to claim your Publons credit. I understand this statement.

Yes Choose an item.
